# Metabolic stimulation-elicited transcriptional responses and biosynthesis of acylated triterpenoids precursors in the medicinal plant *Helicteres angustifolia*

**DOI:** 10.1186/s12870-022-03429-8

**Published:** 2022-02-25

**Authors:** Yuying Huang, Wenli An, Zerui Yang, Chunzhu Xie, Shanshan Liu, Ting Zhan, Huaigeng Pan, Xiasheng Zheng

**Affiliations:** Institute of Medicinal Plant Physiology and Ecology, School of Pharmaceutical, Guangzhou University of Chinese Medicine, 232 Waihuandong Road, Higher Education Mega Center, Panyu District, Guangzhou, 510405 China

**Keywords:** *Helicteres Angustifolia*, Acylated triterpenoids, Transcriptome, Functional expressions, Oxidosqualene cyclases, Cytochrome P450

## Abstract

**Background:**

*Helicteres angustifolia* has long been used in Chinese traditional medicine. It has multiple pharmacological benefits, including anti-inflammatory, anti-viral and anti-tumor effects. Its main active chemicals include betulinic acid, oleanolic acid, helicteric acid, helicterilic acid, and other triterpenoid saponins. It is worth noting that some acylated triterpenoids, such as helicteric acid and helicterilic acid, are characteristic components of *Helicteres* and are relatively rare among other plants. However, reliance on natural plants as the only sources of these is not enough to meet the market requirement. Therefore, the engineering of its metabolic pathway is of high research value for enhancing the production of secondary metabolites. Unfortunately, there are few studies on the biosynthetic pathways of triterpenoids in *H. angustifolia*, hindering its further investigation.

**Results:**

Here, the RNAs of different groups treated by metabolic stimulation were sequenced with an Illumina high-throughput sequencing platform, resulting in 121 gigabases of data. A total of 424,824 unigenes were obtained after the trimming and assembly of the raw data, and 22,430 unigenes were determined to be differentially expressed. In addition, three oxidosqualene cyclases (OSCs) and four Cytochrome P450 (CYP450s) were screened, of which one OSC (*HaOSC1*) and one CYP450 (*HaCYPi3*) achieved functional verification, suggesting that they could catalyze the production of lupeol and oleanolic acid, respectively.

**Conclusion:**

In general, the transcriptomic data of *H. angustifolia* was first reported and analyzed to study functional genes. Three OSCs, four CYP450s and three acyltransferases were screened out as candidate genes to perform further functional verification, which demonstrated that *HaOSC1* and *HaCYPi3* encode for lupeol synthase and β-amyrin oxidase, which produce corresponding products of lupeol and oleanolic acid, respectively. Their successful identification revealed pivotal steps in the biosynthesis of acylated triterpenoids precursors, which laid a foundation for further study on acylated triterpenoids. Overall, these results shed light on the regulation of acylated triterpenoids biosynthesis.

**Supplementary Information:**

The online version contains supplementary material available at 10.1186/s12870-022-03429-8.

## Introduction

**Fig. 1:**
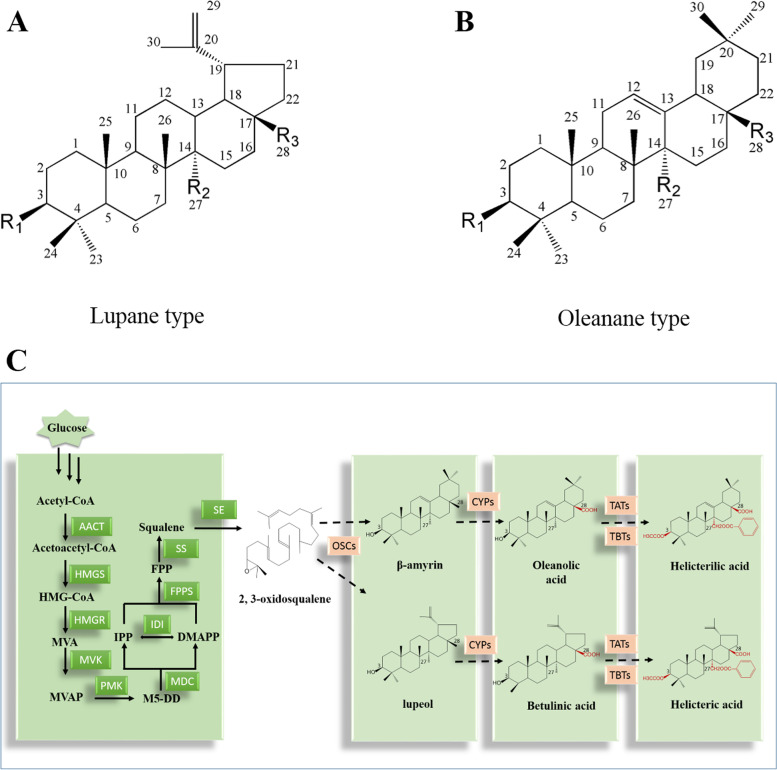
Two parent scaffold types of main triterpenoids and the diagram of a speculated triterpenoids biosynthetic pathway in *H. angustifolia*. **A** Lupane type parent scaffold. **B** Oleanane type parent scaffold. **C** Diagram of the speculated triterpenoids biosynthetic pathway. Enzymes related to the triterpenoids biosynthesis were shown in squares. Solid arrows represent validated steps and dotted arrows represent hypothetical steps. AACT: acetyl-CoA C-acetyltransferase; HMGS: hydroxymethylglutaryl-CoA synthase; HMGR: hydroxymethylglutaryl-CoA reductase; MVK: mevalonate kinase; PMK: phosphomevalonate kinase; MDC: diphosphomevalonate decarboxylase; IDI: isopentenyl-diphosphate Delta-isomerase. FPPS: farnesyl pyrophosphate synthase. SS: squalene synthase; SM: squalene monooxygenase; OSCs: Oxidosqualene cyclases; CYPs: cytochrome P450 enzymes; TATs: triterpenoids acetyl transferases; TBTs: triterpenoids benzoyl transferases

*Helicteres angustifolia*, a conversant plant in *Helicteres* within the family Malvaceae, is a traditional medicinal herb used in Southern China [1]. Ethanolic and aqueous extracts of *H. angustifolia* are often used clinically to treat influenza, headache, carbuncles, hemorrhoids, tonsillitis, pharyngitis, parotitis, inflammatory diseases, and cancer, according to modern pharmacological studies [2–4]. These pharmacological benefits are caused by its chemical components, particularly triterpenoids [5]. So far, 14 triterpenoids have been isolated and identified from *H. angustifolia* (summarized in Table 1), including betulinic acid [6], oleanolic acid [7], helicteric acid and helicterilic acid [6]. These compounds can be grouped into two parent scaffold types, namely, the lupane type (Fig. 1A) and the oleanane type (Fig. 1B). Intriguingly, some of these pentacyclic triterpenoids, like helicteric acid and helicterilic acid, are decorated with acyl groups at the C-3 and/or C-27 positions, which are the characteristic components of the genus *Helicteres* [8, 9].

**Table 1:** Main triterpenoids in *H. angustifolia*

Parent nucleus	R_**1**_	R_**2**_	R_**3**_	Compound name
**A**	OH	CH_3_	COOH	betulonic acid
	OCOCH_3_	CH_2_OCOBz	COOCH_3_	helicteric acid methyl ester
	OCOCH_3_	CH_2_OCO(4-OH)-Bz	COOCH_3_	27-(p-hydroxyl) benzoylhelicteric acid methyl ester
	OCOCH_3_	CH_2_OCOBz	COOH	helicteric acid
	OCOCH_3_	CH_3_	OH	betulin-3-acetate
	OCOCH_3_	CH_3_	COOH	betulonicacid-3-acetate
	OH	CH_2_OCOBz	COOH	27-benzoybetulinic acid
	OH	CH_2_OCOBz	COOCH_3_	27-benzoybetulinic acid methyl ester
**B**	OH	CH_3_	COOH	oleanic acid
	OCOCH_3_	CH_2_OCOBz	COOH	helicterilic acid
	OCOCH_3_	CH_2_OCOBz	COOCH_3_	helicterilic acid methyl ester
	OH	CH_2_OCOBz	COOCH_3_	3β-hydroxyphenyl-27-benzoyoleanic acid
	OCOCHCH(4-OH)Bz	CH_3_	COOH	3-o-p-coumaroyloleanolic acid
	OCOCH_3_	CH_2_OCO(4-OH)Bz	COOCH_3_	27-(p-hydroxyl) benzoylhelicteric acid methyl ester

A and B correspond to parent nucleus of lupane type and the oleanane type, respectively

Modern pharmacological studies indicated that acylated triterpenoids exhibit multiple pharmacological effects, such as easing transaminase [9], promoting apoptosis in cancer cells, and providing anti-tumor [4], anti-hepatitis B virus [10], anti-liver fibrosis [11, 12], and liver-protecting effects [13, 14]. However, in *H. angustifolia*, the content of these compounds is limited. Hence, exclusive reliance on isolating ingredients from herbal materials is impractical. A significant increase in the content of target products in native plants through biosynthesis and gene regulation could be a more effective strategy for addressing this shortage than the traditional extraction approach [15, 16]. However, the complete lack of research on partial key biosynthetic pathways of triterpenoids in *H. angustifolia*, which makes it difficult to improve the content of acylated triterpenoids by means of transcriptional regulation and genetic engineering. Due to the rapid development of high-throughput sequencing, de novo transcriptome analyse provides a powerful tool for screening candidate genes involved in the biosynthesis pathway of target component in plants without genomic reference, which will support further genetic engineering research [17, 18].

**Fig. 2:**
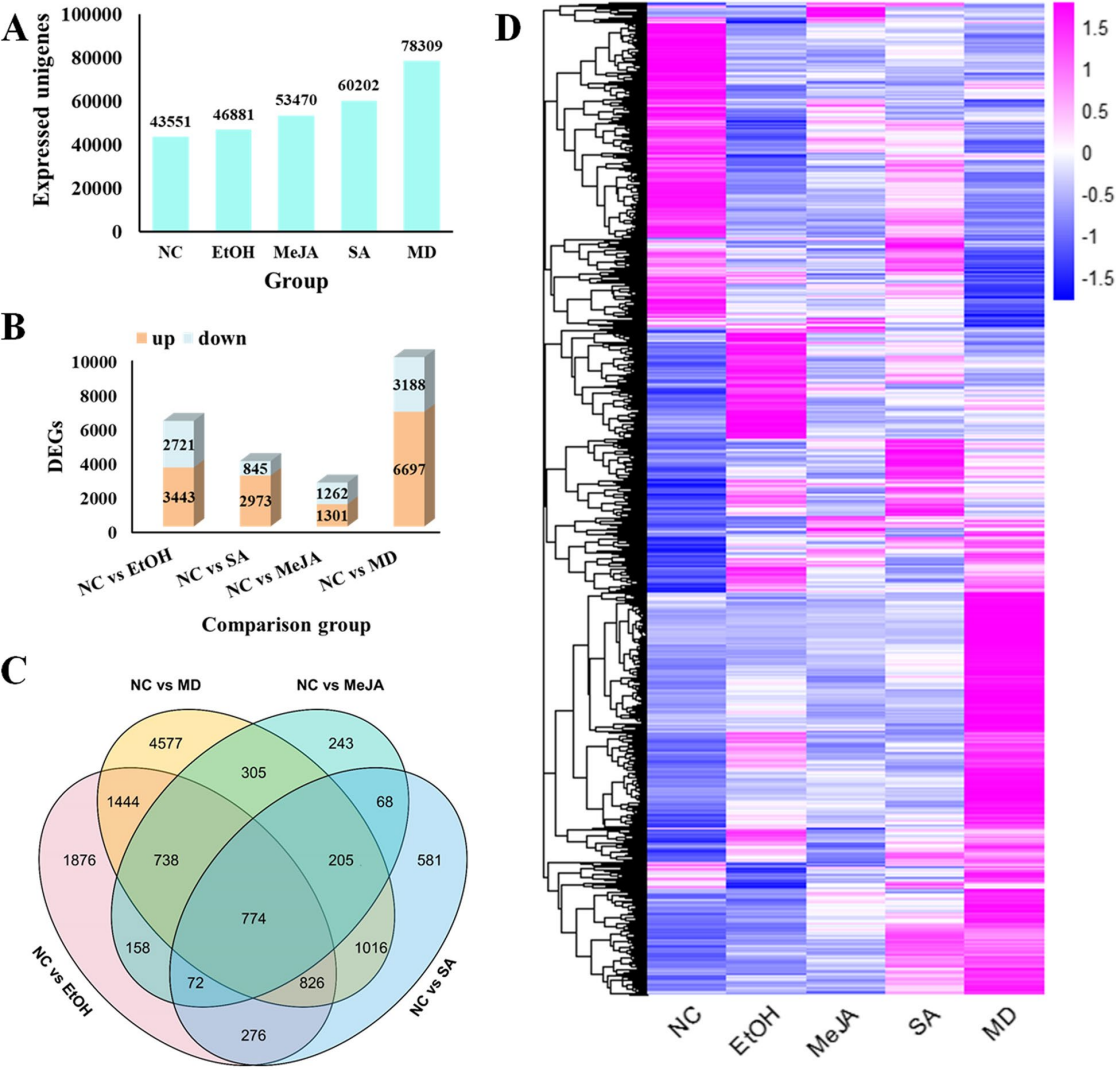
Identification of DEGs among five treatment groups. **A** Numbers of expressed unigenes among five different groups; **B** Numbers of DEGs between treatment groups; **C** Venndiagram showing the DEGs among five treatment groups; **D** Heatmap showing the hierarchical cluster of DEGs among five different treatment groups

Upstream biosynthesis pathways for triterpenoid have been largely clarified [19, 20], mainly through the mevalonate pathway [21, 22] (Fig. 1C). In this pathway, acetyl-CoA is catalyzed by a series of enzymes to produce the C5 unit IPP, which can be transformed into the isomer DMAPP by the IDI (isopentenyl diphosphate isomerase). IPP and DMAPP can be catalyzed into GPP, FPP, and GGPP through a series of prenyl transferases (GPPS, FPPS, and GGPPS). Among them, FPP is an important intermediate for triterpenoid biosynthesis. Two units of FPP can be catalyzed by the squalene synthase (SS) in a tail-to-tail manner to yield the hydrocarbon squalene. Subsequently, another important precursor 2, 3-oxidosqualene, is generated under the catalysis of the squalene monooxygenase (SM) with the presence of O_2_ and the coenzyme NADPH [23, 24]. 2, 3-oxidosqualene can then be derived into various triterpenoid skeletons under the catalysis of oxidosqualene cyclases (OSCs) [25]. Based on those known compounds and intermediates, downstream synthetic routes of triterpenoids in *H. angustifolia* were speculated. As showed in Fig. 2, OSCs involved in the triterpenoid synthesis pathway in *H. angustifolia* are mainly lupeol synthase and β-amyrin synthase, which act on 2, 3-oxidosqualene to produce lupeol and β-amyrin, respectively. Then, cytochrome P450 enzymes (CYP450s) are responsible for modifying lupeol and β-amyrin by C-28 oxidation to form betulinic acid and oleanolic acid. The acylation reaction is presumably as the final step in the modification of those triterpenoids of *H. angustifolia*. Those unique triterpenoids, such as helicteric acid and helicterilic acid, are formed by acetylation at C-3 and benzoylation at C-27 on precursors like betulinic acid and oleanolic acid (Fig. 1C).

Triterpenoids are important metabolic substances [26, 27], and their biosynthesis can be affected by the metabolic stimulation of their source plants, including exogenous phytohormone treatment and mechanical damage [28, 29]. In this study, transcriptomes of *H. angustifolia* samples under different treatments of metabolic stimulation were sequenced and analyzed to obtain a genetic basis for the biosynthesis of the metabolic regulation of triterpenoids in vivo. Moreover, cloning and functional characterization of two key enzymes encode genes revealed their involvement in the biosynthesis of the direct precursors of medicinally acylated triterpenoids. In general, results presented here support our understanding of the regulation and synthesis of triterpenoids in *H. angustifolia*, laying the groundwork for further studies of the potential manipulation of this pharmaceutical resource.

## Materials and methods

### Plant material and metabolic stimulation treatment

*H. angustifolia* was collected in Guanzhu Town, Maoming City, Guangdong Province in 2015, and then planted in the medicinal botanical garden of Guangzhou University of Chinese Medicine (GZUCM). *H. angustifolia* used in this experiment were all collected from the medicinal botanical garden of GZUCM. Permission was not necessary for collecting these species, which have not been included in the list of national key protected plants. The formal identification of the plant material was undertaken by Prof. Chaomei Pan (GZUCM). A voucher specimen of *H. angustifolia* was deposited at the Chinese medicine herbarium of GZUCM (voucher number 440923LY0127)*.* Healthy leaves of cultivated *H. angustifolia* with a length of 5 cm and a width of 1.5 cm were selected as experimental samples, which were rinsed with water, removing sundries on the surface of leaves, and excess water was sucked up by absorbent papers. Methyl jasmonate (MeJA) and salicylic acid (SA) with concentrations of 100–600 μM were prepared and sprayed on the surface of plant leaves. Taking the surface of the leaves as the degree of basic wetting, after 24 h of treatment, the content of total triterpenoids in *H. angustifolia* was analyzed, and the optimal treatment concentration was determined. Fifteen individual plants with the same growth stage were separated into 5 groups. The mechanical damage (MD) group was scratched with a scissor for one-third of the blade area of its leaves. The MeJA and SA groups were sprayed with the optimal concentration of MeJA and SA on their leaves, respectively. The solvent control group was treated the same as MeJA group, only with a different solvent of EtOH. The negative control group (NC) contains three plants without any treatment. Leaves from these five groups were sampled 24 h after treatment and placed in liquid nitrogen for total triterpenoids content determination and RNA extraction.

### Chemical analyses of total triterpenoids

Procedures of total triterpenoids extraction and measurement were adapted from Cai et al. [30] and Oludemi et al. [31]. Leaves were sampled and extracted with 80% ethanol at a material-to-liquid ratio of 1:30 and an ultrasonic extraction time of 2 h at 70 °C. Then the dry extract was redissolved in an n-butanol solution. With 5% vanillin-glacial acetic acid solution and perchloric acid as the chromogenic agents, the mixture’s absorbance was measured with ultraviolet spectrophotometry at 548 nm. Betulinic acid served as a reference chemical marker and was utilized to draw the standard curve. The curve showed a good linear relationship, with an equation of y = 4.2893x + 0.0136 (r2 = 0.9995). Total triterpenoids extraction and measurement were performed in independent triplicates.

### RNA extraction and transcriptome sequencing

Total RNA of 15 samples (Fig. S1) was extracted using a plant total RNA purification kit, following the manufacturer’s instructions (TIANGEN, China). The integrity and quantity of the RNA samples were analyzed by 1% agarose gel electrophoresis and with a NanoDrop 2000C Spectrophotometer (Thermo Scientific, USA). To analyze the transcriptome, qualified RNA samples were sent to Majorbio (Shanghai, China) to construct the cDNA library and then be sequenced on an Illumina HiSeq4000 (Illumina Inc., San Diego, CA, USA). Paired-end reads were generated and examined with the fastx_toolkit_0.0.14 software (http://hannonlab.cshl.edu/fastx_toolkit/) to assess the quality of those reads.

### Differential expressed genes (DEGs) analysis

Fragments per kilo bases per million reads (FPKM) was measured to gauge the relative expression levels, and the FPKM value of the transcripts was determined using the software RSEM v1.2.15 (http://deweylab.github.io/RSEM/) with default parameters. The DESeq2 version 1.10.1 software was used to analyze the raw counts based on the negative binomial distribution. Expressions of transcripts in different groups were calculated using the FPKM method. The absolute value of the log2 multiple of the FPKM expression ratio between pairwise groups was greater than 1 and the false discovery ratio was less than 0.05 as the standard. DEGs screening was carried out. KEGG enrichment analysis of the DEGs was performed with the KOBAS version 2.1.1 (http://www.genome.jp/kegg/). Nine differentially expressed unigenes related to triterpenoid biosynthesis were selected for qPCR verification. The gene and primer information was presented in Table S1.

### Mining of candidate genes involved in triterpenoid biosynthesis

Three approaches were employed to screen genes related to the biosynthesis of triterpenoids. Firstly, BLASTx was performed using the resulting unigenes mentioned above against public protein databases, including NR, Swiss-Prot, KEGG, GO, PFAM, and COG (E value< 0.00001). Secondly, BLAST-p was performed using the protein sequences of a set of characterized genes against the Transdecoder-predicted peptide sequences of the *H. angustifolia* transcriptome assembly (e-value <1e-5, identify > 40%, score > 200). Lastly, using the Pfam profiles (PF13243.6, PF13249.6, PF00067.22, and PF02458.15) as queries, pfamscan based on the HMMER suite (http://hmmer.janelia.org/) was conducted (e-value < 10^− 5). Positive hits were manually inspected by confirming the presence of the corresponding domains, which were considered as the putative genes.

### Sequence analysis of candidate enzyme proteins and weighted gene co-expression network analysis (WGCNA)

Those candidate gene sequences verified by sequencing were analyzed for the open reading frame (ORF) through the CLC software and were translated into amino acid sequences. The online ExPASy-ProtParam tool (https://web.expasy.org/protparam/) was used to predict physical and chemical properties of those proteins. SOPMA (https://npsa-prabi.ibcp.fr/cgi-bin/npsa_automat.pl?page=npsa_sopma.html) and SWISS-MODEL (https://swissmodel.expasy.org) online tools were employed to predict secondary and tertiary structure of target proteins. Conserved motif and domain of candidate enzyme proteins were analyzed using the online analysis software MEME (http://meme-suite.org/tools/meme) and Pfamscan (https://www.ebi.ac.uk/Tools/pfa/pfamscan/). Phylogenetic analysis of protein sequences was carried out by the MEGA 7.0 software. All selected protein sequences were firstly aligned and exported in the MEGA format. Then the phylogenetic tree was constructed by the Neighbor-joining (NJ) method with the Poisson model using the MEGA alignment output. The bootstrap value was set as 1000 with other parameters as default. The online tool Evolview (https://www.evolgenius.info/evolview/#login) was used to edit the evolutionary tree. Expression data was normalized by square root transformation, then be used to infer co-expression gene network modules employing the WGCNA R package according to step-by-step network construction and the module detection method, soft-thresholding method was used to select a proper power-law coefficient β, and a dynamic hierarchical tree cut algorithm was used to detect the co-expression modules [32, 33].

### Recombinant expressions

The open reading frame (ORF) of candidate genes was amplified from cDNA, using primers summarized in Table S2. The resulting amplicon was conducted to the construction of recombinant vector, which was subsequently transferred into *Escherichia coli* for preservation after sequencing verification. ORFs of candidate OSCs and CYP450s were amplified with gene-specific primers that contained *SalI* and restriction sites, respectively. The PCR products were subcloned into an expression vector pESC-TRP to create pESC-TRP-OSCs and pESC-TRP-CYP450s. After verifying the integrity of those cloned genes through Sanger sequencing, recombinant plasmids were transformed into *S. cerevisiae* WAT11 by lithium acetate (LiAc) transfer method, adapting specific operation referred to the previous literature [34]. After being induced by 2% D-galactose, the recombinant yeast activates the GAL10 and GAL1 promoter on the pESC-TRP vector, thus inducing the expression of the target (6×His)-tagged proteins. The western-blot method was used to detect the expression level of the fusion protein at different time points to determine the optimal induction time, through specific methods referred to in the literature reported previously [35]. And ORFs of TATs and TBT were subcloned into an N-terminal 6×His tag fusion expression vector pCOLD-TF. Upon sequence confirmation, recombinant plasmids were then transferred into *E. coli Rossetta* (DE3) for heterologous expression. All the gene-specific forward and reverse primers were displayed in Table S3.

### Subcellular location of HaCYPi3 proteins

Full-length putative *HaCYPi3* gene sequences without stop codons were fused with an enhanced green fluorescent protein (EGFP) and ligated into the pAN580 vector. The recombinant vectors were then transferred into Arabidopsis protoplasts using the polyethylene glycol (PEG). Protocols used for protoplast isolation and recombinant vector transfer protocols were as described in the previous study [36]. EGFP fluorescent signals were observed using a Zeiss laser scanning microscope (LSM) 800 (Zeiss, Germany).

### GC-MS analysis of yeast extracts and enzymatic assays

The recombinant yeast cells were harvested by centrifugation after 7 days of Gal induction, then added by 20 mL lysis solution (20% KOH, 50% EtOH) for condensation reflux maintained 30 min at a slight boil. After the temperature dropped, the pH value was adjusted to 2.0 with concentrated hydrochloric acid. Yeast lysate was extracted three times with an equal volume of n-hexane. After the hexane phase was dried, the residue was derivatized using bis-N, O-(trimethylsilyl) trifluoroacetamide (Sigma-Aldrich) at 80 °C for 60 min before GC-MS analysis. The GC system was equipped with an HP-5MS (30 m× 0.25 mm× 0.25 μm) column. The sample was injected with a flow rate of 1.2 mL/min and a temperature of 250 °C. The GC oven temperature was programmed from 80 °C (held for 1 min) to 300 °C at 40 °C/min and kept for 10 min. For the identification of metabolites, complete mass spectra was generated by scanning within the mass-to-charge ratio range of 50 to 600. Triterpenoid products in yeast were monitored according to the base peak. Empty pESC-TRP vector was used as the negative control, and standard substance was used as the positive control. Triterpenoid was determined by comparing both the retention time and mass spectra with the authentic standards.

For the characterization of the acyltransferase, an enzymatic assay containing 50 mM Tris-HCl buffer (pH 7.0), 300 mM NaCl, 1 mM dithiothreitol, 30–50 μg protein, 200 μM acyl donor (250 μM acetyl CoA/benzoyl CoA) and substrates (500 μM oleanolic acid/betulinic acid) was carried out in a final volume of 200 μL. The reaction system was placed at 30 °C for 2 h. After the reaction, 500 μL ethyl acetate was added to extract twice and the extracted solution was combined. The residue was redissolved with 200 μL acetonitrile and centrifuged at 16,000 rpm for 5 min, with the supernatant isolated for subsequent HPLC analysis. The HPLC equipment was equipped with a Hypersil Gold AQ-C18 column (250× 4.5 mm × 5 μm). Samples (20 μL) were injected at a column temperature of 25 °C. The liquid phase consisted of solvent A (0.2% phosphoric acid aqueous solution) and solvent B (acetonitrile). The elution procedure was set as followed: 0–60 min, 80% B, 60–75 min, 80–100% B, with a flow rate of 1.2 mL/min. The detection wavelength was set at 210 nm.

### Statistical analysis

Three biological replicates were carried out for each experiment, and statistical analysis was performed using the IBM SPSS Statistics 23 statistical software. A Shapiro-Wilk test was used to test whether the data was normally distributed. If yes, the t-test of independent samples was used for pairwise comparison. One-way Analysis of Variance was used for comparison between groups, in which homogeneity of Variance was tested for the data, assuming that the Variance was not homogenous. A significance *P*> 0.05 was used to indicate homogeneity of variance. The Bonferroni comparison test was used. The Dunnett’s T3 multiple comparison test was used when in heterogeneity variance, and the data obtained were stated as (mean ± standard) deviation, with a *P*< 0.05 indicated statistically significant differences. If the single-factor data did not follow normal distribution, Kruskal Wallis single-factor ANOVA (K sample) and Mann-Whitney U (two samples) rank sum test were used. A *P< 0.05* indicated that the difference was statistically significant. Data statistics were performed using the Graph Pad Prism 8.0 software.

## Results

### Transcriptome analysis induced by metabolic stimulation

The optimal concentration of 500 μM methyl jasmonate (MeJA) and 400 μM salicylic acid (SA) was determined by the single factor experiment (Fig. S2, Table S4). Consequently, the total triterpenoids content of samples from five groups was measured. The results showed that elevated contents of total triterpenoids in the MD-, MeJA-, and SA-induced groups were observed, compared to the NC group (Fig. S3, Table S5). This result was consistent with other research, suggesting that the synthesis of triterpenoids could be affected by metabolic stimulation [37]. Then the RNAs of different groups treated by metabolic stimulation were sequenced with an Illumina high-throughput sequencing platform to obtain an overview of the transcriptome profile of *H. angustifolia* among those five groups. In all, 121.62 Gb high-quality data (at least 6.91 Gb for each sample and 23.46 Gb for each group) with 43.67%, GC contents were generated from 15 samples. The raw data and quality control data were characterized in Table S6. Then 581,934 transcripts and 424,824 unigenes were obtained after trimming and assembly of the clean data. The mean size of the unigenes was 655 bp, with a length N_50_ of 1300 bp (Table 2).

**Table 2:** Analysis of the assembly transcriptome

Items	Number
**Total sequence base**	381,166,620
**Total transcripts**	581,934
**Total unigenes**	424,824
**Largest (bp)**	16,851
**Smallest (bp)**	201
**Length N**_**50**_ **(bp)**	1294
**Mean Length(bp)**	655

Sequence annotation was performed using the BLAST algorithm against six public databases. In all, 196,252, 179,036, 167,974, 25,390, 110,706, and 138,066 unigenes, respectively, were annotated in these databases (Fig. S4, Table S7). In total, among the 424,824 unigenes generated by transcriptional sequencing, 245,709 (57.84%) received at least one hit in those databases, with 13,320 unigenes sharing common annotation (Fig. S5).

### Analysis of DEGs under different treatments

The statistics of expressed genes showed that the control group had the least number of genes, while the MD group had the most (Fig. 2A). Then transcriptome statistics of those five groups were compared pairwise to establish potential DEGs among different groups. Compared to the NC group, there was the greatest amount of DEGs in the MD group (Fig. 2A), and the number of up-regulated DEGs in each metabolic stimulation treatment group and EtOH group were higher than that of the down-regulated genes (Fig. 2B) However, there is a very interesting phenomenon that EtOH group appears to be more influential on gene expression than either of the hormone treatments. Ethanol was selected as the solvent because ethanol treatment had no significant effect on the content of total triterpenoids. This phenomenon revealed that although ethanol has no significant effect on the expression of genes related to triterpenoids pathway, it may affect the expression of other genes. The Venn diagram presented in Fig. 2C indicates that 774 DEGs were shared among the four aforementioned pairwise comparisons, and a total of 22,430 DEGs were obtained among the four comparison groups. Heat maps were produced based on the expression patterns of all DEGs, and these could be divided into eight clusters (Fig. 2D). KEGG enrichment analyses of DEGs in four pairwise groups (Fig. S6, Tables S8, S9, S10, S11) suggested that pathways with the most enrichment were mostly associated with triterpenoid synthesis, revealing that the expression of genes related to triterpenoid synthesis was up-regulated after metabolic stimulation, leading to the enhancement of triterpenoid accumulation for resistance, which is consistent with the results of previous research [29, 38].

**Fig. 3:**
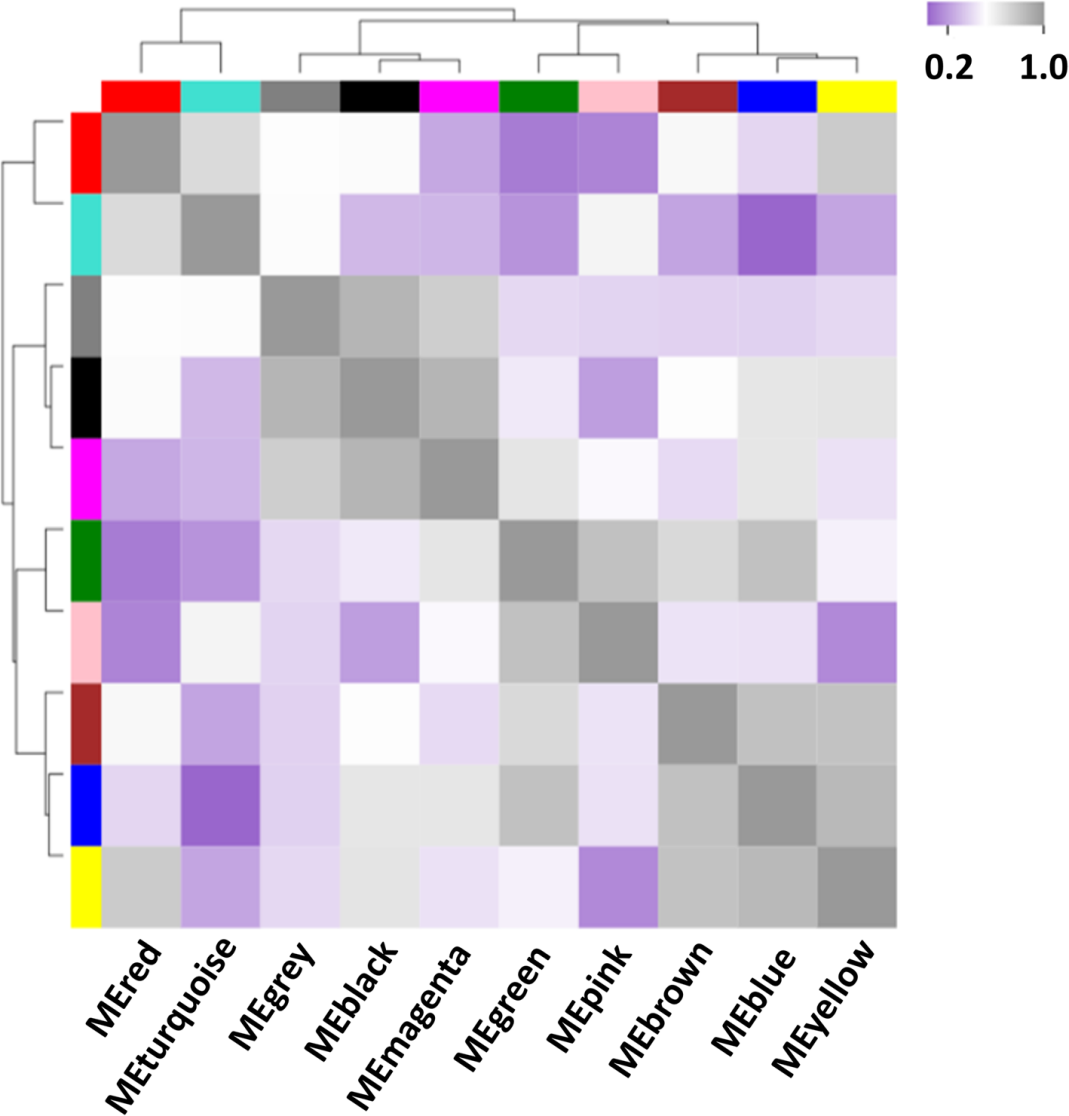
Gene co-expression modules in hormone and mechanical damage-treated transcriptome indicated the cluster hierarchical tree constructed by the eigengenes of the modules and the correlation coefficient between modules with a heatmap

In addition, weighted gene co-expression network analysis (WGCNA) was conducted to further investigate potential genes involved in the triterpenoid biosynthesis of *H. angustifolia*. All DEGs were submitted to construct a scale-free co-expression network. The dynamic hierarchical tree algorithm was employed to divide cluster trees constructed with those DEGs, resulting in 10 co-expression modules. These modules were named according to their colors, namely the blue (1619 genes), brown (891 genes), turquoise (2505 genes), grey (17 genes), yellow (759 genes), pink (55 genes), green (673 genes), black (87 genes), magenta (52 genes), and red (115 genes) module (Fig. 3). Genes within each module were selected for enrichment in the KEGG pathway. Statistically significantly enriched genes (*P* < 0.05) were filtrated for further analyses. Results showed that five modules (Blue, turquoise, black, green and magenta), consisting of 4936 genes in total, were related to triterpenoid biosynthesis.

### Quantitative real-time PCR (qRT-PCR) to verify the DEGs

**Fig. 4:**
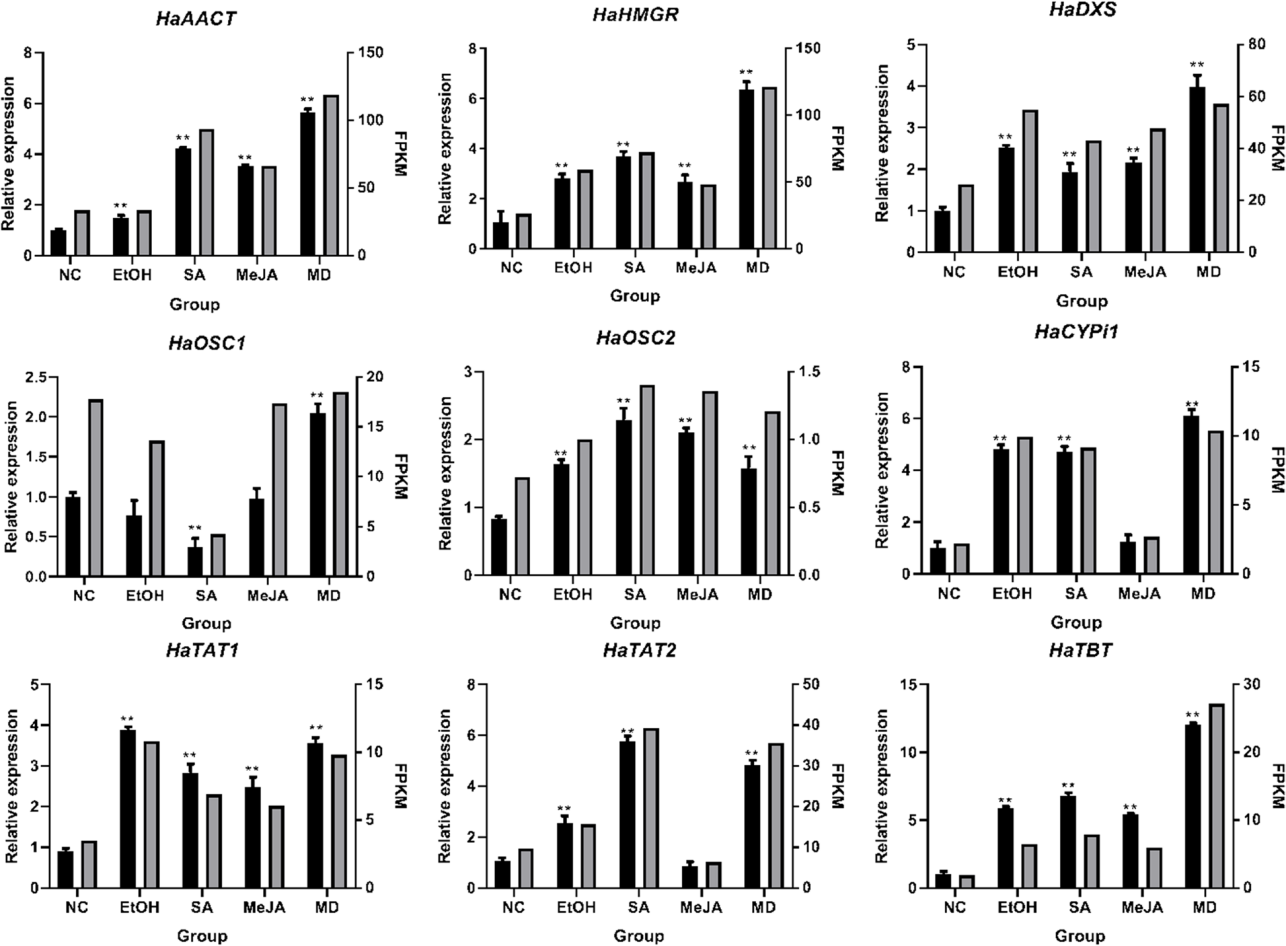
Validation of genes involved in triterpenoid biosynthesis using qRT-PCR. The left Y-axis and black bars represents the relative expression of qPCR, while the right Y-axis and grey bars exhibits the FPKM value of the RNA-Seq data. ** indicates *P*< 0.01

Based on transcriptome annotation results, we screened 41 genes, whose annotations were related to triterpenoid skeleton synthesis according to KEGG pathway, and their expression patterns (Fig. S7, Table S12) were analyzed thereafter. Among them, 18 genes presented significantly different expression levels among five treatment groups, nine of which were chosen to validation on their expression level using qRT-PCR, aiming to further verify the reliability of our RNA-Seq data. As shown, almost all of these genes were significantly increased after treatment with hormones and mechanical damage, which is consistent with the RNA-Seq data in expression (Fig. 4, Table S13), confirming the accuracy of our transcriptome data.

### Cloning and sequence analysis of candidate genes

In order to elucidate the biosynthetic pathway of acylated triterpenoid precursors and shed light on acylated triterpenoids biosynthesis in *H. angustifolia*, putative key enzymes encoding genes, including three OSCs (named *HaOSC1*, *HaOSC2* and *HaOSC3*) and four CYP450s (named *HaCYPi1*, *HaCYPi2*, *HaCYPi3* and *HaCYPi4*), were targeted for functional analysis. Their corresponding protein sequences were summarized and exhibited in Table S14 and physicochemical properties of these proteins were documented in the Table S15, two-dimensional structure information of these proteins was classified in the Table S16, while three-dimensional structures were displayed in Fig. S8.

**Fig. 5:**
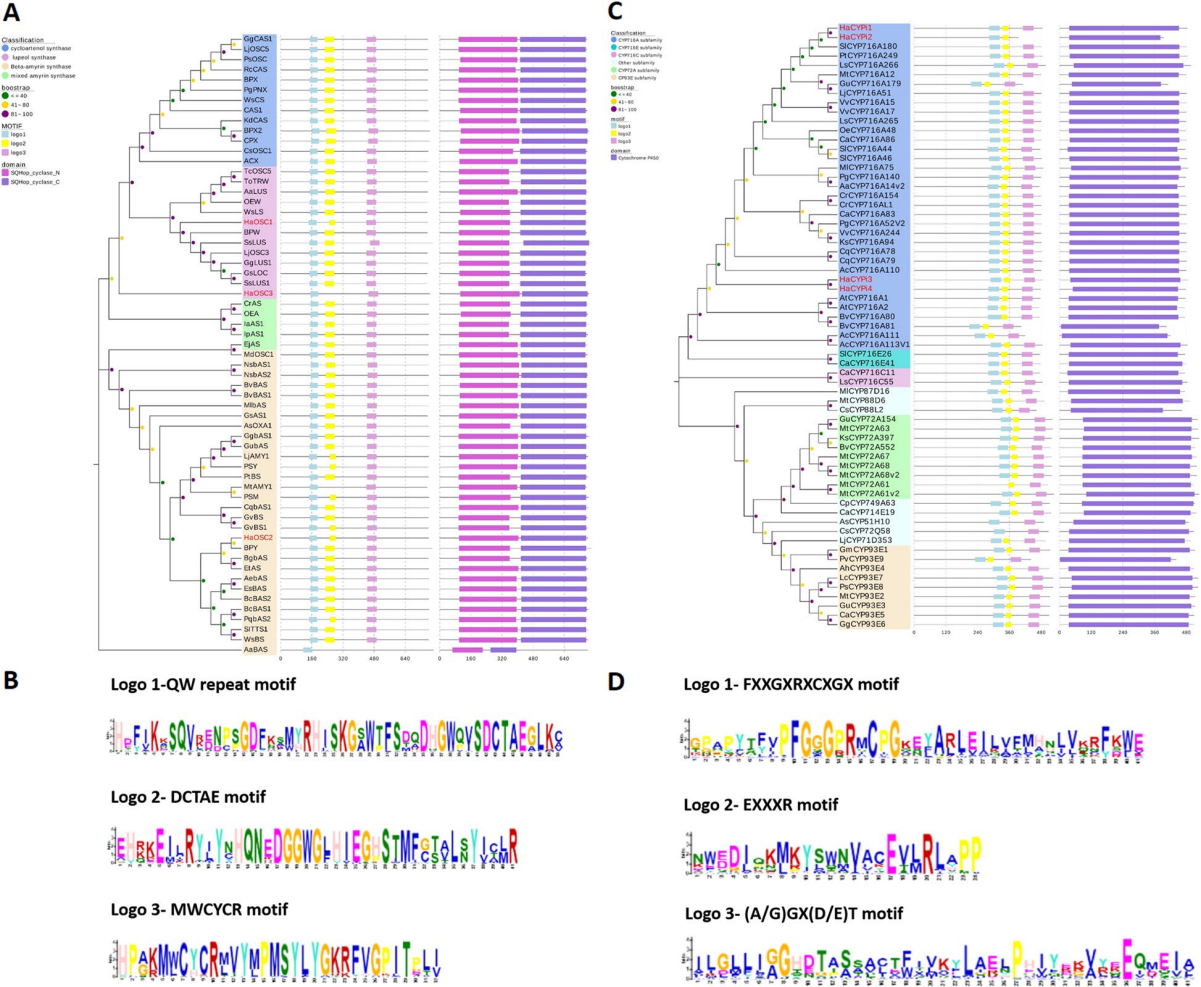
Analysis of amino acid sequences of OSCs and CYP450s. **A** Phylogenetic tree, conserved domain and conserved motif analysis of OSCs; **B** Conservative motifs of OSCs corresponding sequences; **C** Phylogenetic tree, conserved domain and conserved motif analysis of CYP450s; **D** Conservative motifs of CYP450s corresponding sequences

Phylogenetic analyses of three OSCs with a set of well characterized OSCs revealed that HaOSC1 and HaOSC3 were grouped into the lupeol synthase clade, while HaOSC2 was classified as a β-amyrin synthase (Fig. 5A, Table S17). In addition, the conserved domain and motif analysis of candidate HaOSCs showed that they had high similarity to the plant oxidosqualene cyclase (OSC) family. They all contained conserved SQHOP-cyclase-N and SQHOP-cyclase-C domains with three conserved motifs, including DCTAE, MWCYCR and QW repeat (Fig. 5B). Among them, the DCTAE motif might be crucial for the interaction between the protein and its substrate [38]. The QW repeat is a negatively charged aromatic region that equalizes carbocation in the cyclization reaction, which may play a role in the structural stabilization of proteins [39].

The results of phylogenetic analyses also implied that four CYP450 candidate genes were well documented into the CYP716A subfamily (Fig. 5C, Table S18), which primarily includes CYP450 enzymes with a C-28 oxidation function [40, 41]. Conserved cytochrome P450 domain (heme active sites) shared by candidate CYP450 genes were found except for *HaCYPi2*, including the oxygen-binding domain (A/G)GX(D/E)T, the K helix (EXXXR), and the C-terminal heme binding domain (FXXGXRXCXGX) (Fig. 5D). Among them, the heme binding domain is the main characteristic structure for the identification of P450 protein [42].

### Heterologous expression and functional characterization of three OSCs

**Fig. 6:**
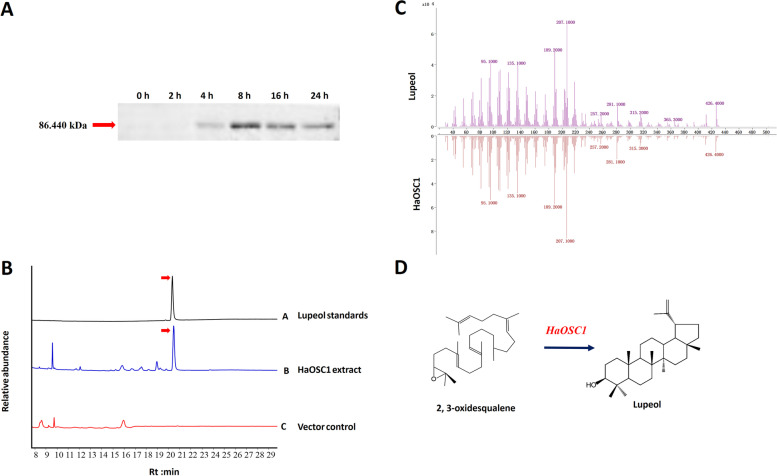
Functional analysis of *HaOSC1*. **A** Westenrn blot analysis of pESC-TRP-*HaOSC1* transformants; **B** Extracted ion chromatograms of lupeol standards (**A**), yeast cell transformed with expression constructs pESC-TRP- *HaOSC1* (**B**), negative control using an empty vector pESC-TRP (**C**). **C** The MS data of lupeol and pESC-TRP- *HaOSC1.* **D** Schematic diagram of catalytic function of *HaOSC1*

Three candidate OSCs were functionally validated by expressing their ORFs in the expression vector pESC-TRP. Recombinant plasmids were transferred into yeast (*S. cerevisiae* strain WAT11) by the lithium acetate conversion method. Protein expression of the recombinant yeast within 24 h was investigated, which implied that the recombinant yeast pESC-TRP-HaOSC1 and pESC-TRP-HaOSC2 successfully expressed the protein with molecular mass of about 86 kDa (Fig. 6A) and 87 kDa (Fig. S9) except for the recombinant yeast pESC-TRP-HaOSC3, and the protein expression reached the highest level 8 h after induction. Gal-induced transgenic yeast cells were also analyzed for the accumulation of triterpenoids metabolites. Yeast cells were extracted and metabolites were identified through gas chromatography-mass spectrometry (GC-MS) analysis. By comparing the retention time and mass spectra of those enzymatic products with authentic standards (α-amyrin, β-amyrin and lupeol), we found that HaOSC1-expressing transgenic yeast accumulated lupeol, which was not present in the control yeast cells transformed with empty vector (Fig. 6B-C). The yeast expression experiments were repeated three times, always with very similar results. These results clearly manifested that *HaOSC1* encods a lupeol synthase, which could use 2, 3-oxidosqualene in yeast as the substrate to synthesize the target product lupeol (Fig. 6D).

### Functional validation of four CYP450s

**Fig. 7:**
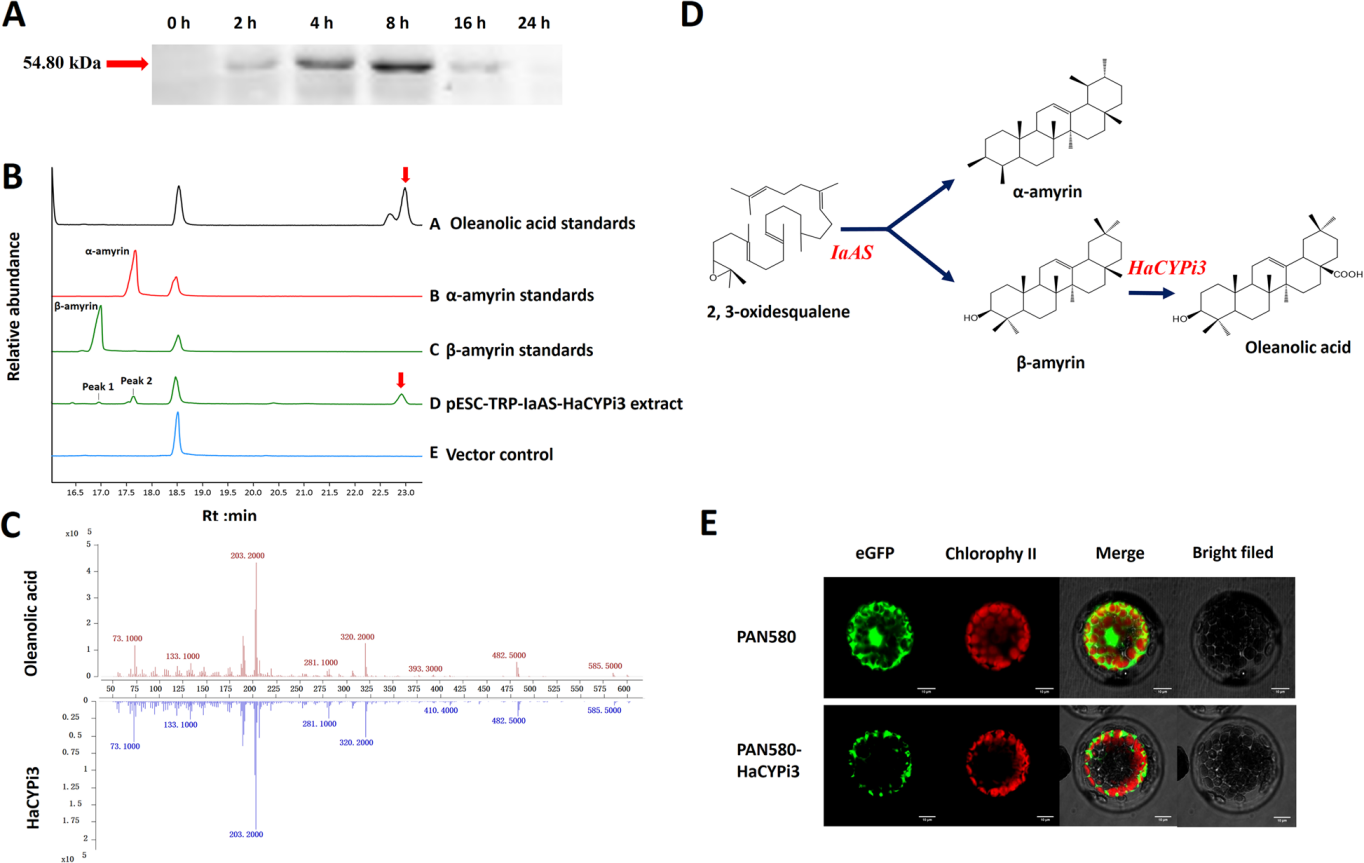
Functional analysis of *HaCYPi3*. **A** Protein expression analysis of *HaCYPi3*; **B** Extracted ion chromatograms of oleanolic acid standards (**A**), α-amyrin standards (**B**), β-amyrin standards (**C**), yeast cell transformed with expression constructs pESC-TRP-*IaAS* and pESC-TRP-*HaCYPi3* (**D**), netagive control using an empty vector pESC-TRP (**E**). **C** The MS data of oleanolic acid standards and *IaAS-HaCYPi3* co-expression yeast cell extracts*.* **D** Schematic diagram of catalytic function of *HaCYPi3*

To determine the product specificities of HaCYP450s, ORF of four candidates was expressed in yeast under the control of the gal-inducible GAL10 promoter. Induced cells were further used for Western blot analysis, the fusion protein bands of HaCYPi3 of about 54 kDa was observed. The induction time was also investigated for HaCYPi3, with a result that the protein expression reached the highest level at about 8 h after induction (Fig. 7A).

Based on the previous sequence analysis, HaCYPi3 was predicted to be a potential C-28 oxidase, which might use α-amyrin, β-amyrin and lupeol as substrates. Therefore, we firstly transferred pESC-HaCYPi3 and pESC-HaOSC1 recombinant plasmids into the same yeast for co-expression. The metabolites after induction were analyzed by GC-MS. Unfortunately, the target product betulinic acid was not detected, which suggested that HaCYPi3 failed to encode for a lupeol oxidase. Then, we co-expressed HaCYPi3 with α-/β-amyrin synthase IaAS from *llex asprell* that is identified in our previous work. It is surprising that IaAS and HaCYPi3 co-expressing transgenic yeast accumulated new products compared with empty vector. Except for the corresponding substrate peaks, the retention time and mass spectra of the new product were consistent with that of the oleanolic acid authentic standards (Fig. 7B-C). Consistene results were gained by three repeated experiments, which clearly demonstrated that HaCYPi3 encoded a β-amyrin oxidase, which could use the β-amyrin catalyzed by IaAS in yeast as a substrate to synthesize the target product oleanolic acid (Fig. 7D). Furthermore, the subcellular localization of HaCYPi3 was observed, and results displayed that it was localized in the cytoplasm (Fig. 7E), which is consistent with reports that triterpenoids were synthesized in the cytoplasm [43].

## Discussions

Triterpenoids, as a class of natural products with extensive biological activities, have attracted the attention of many researchers. Currently, the biosynthetic pathways of many triterpenoids have been successfully elucidated, such as ginsenosides [44], ganoderic acid [45] and glycyrrhizic acid [46]. However, acylated triterpenoids, especially benzoylated triterpenoids, are uncommonly seen and only present in plants belonging to the *Helicteres* genus. Hence, *H. angustifolia* can be used as an ideal research material to elucidate the biosynthetic pathway of acylated triterpenoids. The biosynthesis of triterpenoids has been reported to be affected by plant metabolic stimulation, including exogenous plant hormone treatment and mechanical damage [47–49]. Exogenous methyl jasmonate (MeJA) and salicylic acid (SA) can act as signal transduction molecules in plant cells and induce the production of terpenoids, phenols and other secondary metabolites [50, 51]. As a means of abiotic stress, mechanical damage can effectively regulate the growth, development, stress response and metabolites of plants [27]. In this study, the content of total triterpenoids in *H. angustifolia* was analyzed after different concentration hormone stimulation treatments, with the optimal treatment conditions investigated. Furthermore, transcriptome sequencing of *H. angustifolia* under the optimized treatment conditions was carried out to screen genes and assisted in revealing the biosynthetic pathway at molecular level, which provided theoretical basis for the effective development and utilization of triterpenoids in *H. angustifolia*.

One lupeol cyclase and one β-amyrin oxidase were successfully screened and identified by means of metabolic stimulation combined with transcriptomics, and corresponding products lupeol and oleanolic acid were successfully synthesized in *S. cerevisiae*, which clearly reveal the biosynthesis pathways of some important precursors of acylated triterpenoids. Furthermore, acylation is the final and most critical step in the biosynthesis of acylated triterpenoids. Functional identification of the corresponding acyltransferases including acetyl transferases and benzoyl transferases is the target of our research group in the future.

So far, we have successfully spotted three candidate acyltransferases that belong to the BAHD acyltransferase family through phylogenetic analysis (Fig. S10A, Table S16). This family is named after the initials of four representative enzymes: benzylalcohol acetyltransferase, anthocyanin ohydroxycinnamoyltransferase, anthranilate N-hydroxycinnamoyl/benzoyltransferase, and deacetylvindoline acetyltransferase [52, 53]. Domain analysis showed that the transferase family domain existed in these three candidates. And they all possess three amino acid motifs, HXXXD, YFGNC, and DFGWG (Fig. S10B), which are highly conserved in BAHD enzymes. The HXXXD motif is located in the center of the reaction channel, which is considered to facilitate catalytic reactions to form amide or acylated esters [54]. The DFGWG motif is proposed to play a structural role and has a significant impact on the integrity of the CoA binding pocket [55].

Then, prokaryotic expression vectors carrying three candidate acyltransferases coding genes were constructed, and the resulting fusion proteins were induced to express in *Escherichia coli* Rosetta (DE3) strain. Three purified target proteins were successfully obtained by purifying poly (His) (6×His)-tagged proteins using a nickel-nitrilotriacetic acid agarose column (Fig. S11). Unfortunately, functions of the three candidate enzymes failed to be characterized via in vitro enzyme activity test. We hypothesized that prokaryotic expression could not provide appropriate post-translational modifications for these proteins, leading to failure of enzyme activity responses in vitro. Therefore, further investigation on their function shall be carried out through the combination of eukaryotic expression and substrate feeding in the future.

## Conclusion

*Helicteres angustifolia*, a shrub distributed in Southern China and be used in traditional Chinese medicine, is rich in triterpenoids such as betulinic acid, oleanolic acid, helicteric acid, helicterilic acid, and similar derivatives. The biosynthetic pathway of these compounds is of great value due to their valuable medicinal activity. Here, we first reported the transcriptomic study of *H. angustifolia* for the exploration of functional genes. Three OSCs, four CYP450s and three acyltransferases were screened out as candidate genes. Their functional verification demonstrated that HaOSC1 and HaCYPi3 encode for a lupeol synthase and β-amyrin oxidase, respectively. Corresponding products of lupeol and oleanolic acid, the important precursors of acylated triterpenoids, were synthesized successfully. These results in this study built a basis for future analyses of the biosynthetic pathway of acylated triterpenoids, and laid a foundation for future genetic engineering to increase the yield of medicinal triterpenoids in *H. angustifolia*.

## Supplementary Information

**Additional file 1: Figure S1.** The electrophoresis diagrams of RNA extracted from different treatment groups of *H. angustifolia* tree leaves.

**Additional file 2: Figure S2.** Investigation of hormone concentration.

**Additional file 3: Figure S3.** Comparisons of the total triterpenoid content among different treatment groups.

**Additional file 4: Figure S4.** Functional annotation of the transcripts and unigenes against six databases.

**Additional file 5: Figure S5.** Venn diagram indicating the numbers of common and specific expressed unigenes among six databases.

**Additional file 6: Figure S6.** KEGG enrichment results of the DEGs among negative control group and different treatment groups.

**Additional file 7: Figure S7.** The expression pattern of key related genes involved with triterpenoids biosynthesis were shown using a heatmap.

**Additional file 8: Figure S8.** Three dimention structure of target gene coding proteins.

**Additional file 9: Figure S9.** Protein expression analysis of *HaOSC2*.

**Additional file 10: Figure S10.** Analysis of amino acid sequences of TATs (Triterpenoid acetyltransferase) and TBT (Triterpenoid benzoyl transferase).

**Additional file 11: Figure S11.** SDS-PAGE analysis.

**Additional file 12: Table S1.** Primers for qRT-PCR.

**Additional file 13: Table S2.** Primers for amplification of ten putative genes.

**Additional file 14: Table S3.** Gene-specific forward and reverse primers.

**Additional file 15: Table S4.** Effect of different methyl jasmonate and salicylic acid concentrations and on total triterpenoids content.

**Additional file 16: Table S5.** Effects of different metabolic stimulation treatments on total triterpenoids content.

**Additional file 17: Table S6.** Transcriptome sequencing data of the *H. angustifolia.*

**Additional file 18: Table S7.** The overview statistics of annotation.

**Additional file 19: Table S8.** KEGG enrichment of DEGs from the NC vs EtOH comparison.

**Additional file 20: Table S9.** KEGG enrichment of DEGs from the NC vs SA comparison.

**Additional file 21: Table S10.** KEGG enrichment of DEGs from the NC vs MeJA comparison.

**Additional file 22: Table S11.** KEGG enrichment of DEGs from the NC vs MD comparison.

**Additional file 23: Table S12.** Relative expression of genes involved in triterpenoids biosynthesis.

**Additional file 24: Table S13.** The relative expression levels of nine related genes in different treatment groups.

**Additional file 25: Table S14.** Nucleotide sequence of ten candidate genes.

**Additional file 26: Table S15.** Analysis of physicochemical properties of target gene coding proteins.

**Additional file 27: Table S16.** Two-dimensional structure information of of target gene coding proteins.

**Additional file 28: Table S17.** Summary table of OSC genes.

**Additional file 29: Table S18.** Summary table of CYP450 genes.

**Additional file 30: Table S19.** Summary table of BAHD genes.

## Data Availability

The sequencing datasets generated during the current study are available at CNGBdb: CNP0001265 (https://db.cngb.org/search/project/CNP0001265/).

## References

[1] Li K, Lei Z, Hu X, Sun S, Li S, Zhang Z (2015). In vitro and in vivo bioactivities of aqueous and ethanol extracts from Helicteres angustifolia L. root. J Ethnopharmacol.

[2] Li K, Yu Y, Sun S, Liu Y, Garg S, Kaul SC, Lei Z, Gao R, Wadhwa R, Zhang Z (2016). Functional characterisation of anticancer activity in the aqueous extract of Helicteres angustifolia L. Roots. PLoS One.

[3] Yin X, Lu Y, Cheng ZH, Chen DF. Anti-complementary components of Helicteres angustifolia. Molecules (Basel, Switzerland). 2016;21(11). 10.3390/molecules21111506 PMC6273495 27834928

[4] Sun S, Li K, Xiao L, Lei Z, Zhang Z (2019). Characterization of polysaccharide from Helicteres angustifolia L. and its immunomodulatory activities on macrophages RAW264.7. Biomed Pharmacother.

[5] Pan MH, Chen CM, Lee SW, Chen ZT (2008). Cytotoxic triterpenoids from the root bark of Helicteres angustifolia. Chem Biodivers.

[6] Su D, Gao YQ (2017). Helicteric acid, Oleanic acid, and Betulinic acid, three Triterpenes from Helicteres angustifolia L., inhibit proliferation and induce apoptosis in HT-29 colorectal Cancer cells via suppressing NF-κB and STAT3. Signaling..

[7] Wang GC, Li T, Wei YR, Zhang YB, Li YL, Sze SC, Ye WC (2012). Two pregnane derivatives and a quinolone alkaloid from Helicteres angustifolia. Fitoterapia.

[8] Chang YS, Ku YR, Lin JH, Lu KL, Ho LK (2001). Analysis of three Lupane type triterpenoids in Helicteres angustifolia by high-performance liquid chromatography. J Pharm Biomed Anal.

[9] Chen L, Liu J, Ge X, Xu W, Chen Y, Li F, Cheng D, Shao R (2019). Simulated digestion and fermentation in vitro by human gut microbiota of polysaccharides from Helicteres angustifolia L. Int J Biol Macromol.

[10] Huang Q, Huang R, Wei L, Chen Y, Lv S, Liang C, Zhang X, Yin F, Li H, Zhuo L (2013). Antiviral activity of methyl helicterate isolated from Helicteres angustifolia (Sterculiaceae) against hepatitis B virus. Antivir Res.

[11] Biggs BW, Lim CG, Sagliani K, Shankar S, Stephanopoulos G, De Mey M, Ajikumar PK (2016). Overcoming heterologous protein interdependency to optimize P450-mediated Taxol precursor synthesis in Escherichia coli. Proc Natl Acad Sci U S A.

[12] Zhang XL, Chen ZN, Huang QF, Bai FC, Nie JL, Lu SJ, Wei JB, Lin X (2018). Methyl Helicterate inhibits hepatic stellate cell activation through modulation of apoptosis and autophagy. Cell Physiol Biochem.

[13] Lin X, Huang R, Zhang S, Zheng L, Wei L, He M, Zhou Y, Zhuo L, Huang Q (2012). Methyl helicterate protects against CCl4-induced liver injury in rats by inhibiting oxidative stress, NF-κB activation, Fas/FasL pathway and cytochrome P4502E1 level. Food Chem Toxicol.

[14] Wen S, Wei Y, Zhang X, Bai F, Tan S, Nie J, Wei J, Lin X (2019). Methyl helicterilate ameliorates alcohol-induced hepatic fibrosis by modulating TGF-β1/Smads pathway and mitochondria-dependent pathway. Int Immunopharmacol.

[15] Erb TJ, Jones PR, Bar-Even A (2017). Synthetic metabolism: metabolic engineering meets enzyme design. Curr Opin Chem Biol.

[16] Choi KR, Jang WD, Yang D, Cho JS, Park D, Lee SY (2019). Systems metabolic engineering strategies: integrating systems and synthetic biology with metabolic engineering. Trends Biotechnol.

[17] Heather JM, Chain B (2016). The sequence of sequencers: the history of sequencing DNA. Genomics.

[18] Zhang X, Li T, Liu F, Chen Y, Yao J, Li Z, Huang Y, Wang J (2019). Comparative analysis of droplet-based ultra-high-throughput single-cell RNA-Seq systems. Mol Cell.

[19] Haralampidis K, Trojanowska M, Osbourn AE (2002). Biosynthesis of triterpenoid saponins in plants. Adv Biochem Eng Biotechnol.

[20] Biswas T, Dwivedi UN (2019). Plant triterpenoid saponins: biosynthesis, in vitro production, and pharmacological relevance. Protoplasma.

[21] Yang C, Gao X, Jiang Y, Sun B, Gao F, Yang S (2016). Synergy between methylerythritol phosphate pathway and mevalonate pathway for isoprene production in Escherichia coli. Metab Eng.

[22] Kobayashi K, Suzuki M, Muranaka T, Nagata N (2018). The mevalonate pathway but not the methylerythritol phosphate pathway is critical for elaioplast and pollen coat development in Arabidopsis. Plant Biotechnol (Tokyo, Japan).

[23] Wölwer-Rieck U, May B, Lankes C, Wüst M (2014). Methylerythritol and mevalonate pathway contributions to biosynthesis of mono-, sesqui-, and diterpenes in glandular trichomes and leaves of Stevia rebaudiana Bertoni. J Agric Food Chem.

[24] Gastaldo C, Lipko A, Motsch E, Adam P, Schaeffer P, Rohmer M. Biosynthesis of isoprene units in *Euphorbia lathyris* Laticifers vs. Other Tissues: MVA and MEP Pathways, Compartmentation and Putative Endophytic Fungi Contribution. Molecules (Basel, Switzerland). 2019;24(23). 10.3390/molecules24234322 PMC6930671 31779240

[25] Xue Z, Duan L, Liu D, Guo J, Ge S, Dicks J, ÓMáille P, Osbourn A, Qi X (2012). Divergent evolution of oxidosqualene cyclases in plants. N Phytol.

[26] Cao J, Zhang X, Qu F, Guo Z, Zhao Y (2015). Dammarane triterpenoids for pharmaceutical use: a patent review (2005–2014). Expert Opin Ther Pat.

[27] Yin J, Yang J, Ma H, Liang T, Li Y, Xiao J, Tian H, Xu Z, Zhan Y (2020). Expression characteristics and function of CAS and a new beta-amyrin synthase in triterpenoid synthesis in birch (*Betula platyphylla* Suk.). Plant Sci.

[28] Jin ML, Lee WM, Kim OT. Two Cycloartenol Synthases for Phytosterol Biosynthesis in Polygala tenuifolia Willd. Int J Mol Sci. 2017;18(11). 10.3390/ijms18112426 PMC5713394 29140303

[29] Sharma A, Rather GA, Misra P, Dhar MK, Lattoo SK (2019). Jasmonate responsive transcription factor WsMYC2 regulates the biosynthesis of triterpenoid withanolides and phytosterol via key pathway genes in Withania somnifera (L.). Dunal..

[30] Cai C, Ma J, Han C, Jin Y, Zhao G, He X (2019). Extraction and antioxidant activity of total triterpenoids in the mycelium of a medicinal fungus, Sanghuangporus sanghuang. Sci Rep.

[31] Oludemi T, Barros L, Prieto MA, Heleno SA, Barreiro MF, Ferreira I (2018). Extraction of triterpenoids and phenolic compounds from Ganoderma lucidum: optimization study using the response surface methodology. Food Funct.

[32] Wan Q, Tang J, Han Y, Wang D (2018). Co-expression modules construction by WGCNA and identify potential prognostic markers of uveal melanoma. Exp Eye Res.

[33] Yin L, Cai Z, Zhu B, Xu C. Identification of key pathways and genes in the dynamic progression of HCC based on WGCNA. Genes. 2018;9(2). 10.3390/genes9020092 PMC5852588 29443924

[34] Gietz RD, Schiestl RH (2007). High-efficiency yeast transformation using the LiAc/SS carrier DNA/PEG method. Nat Protoc.

[35] Hnasko TS, Hnasko RM (2015). The Western blot. Methods Mol Biol (Clifton, NJ).

[36] Shen Y, Meng D, McGrouther K, Zhang J, Cheng L (2017). Efficient isolation of Magnolia protoplasts and the application to subcellular localization of MdeHSF1. Plant Methods.

[37] de Costa F, Yendo AC, Fleck JD, Gosmann G, Fett-Neto AG (2013). Accumulation of a bioactive triterpene saponin fraction of Quillaja brasiliensis leaves is associated with abiotic and biotic stresses. Plant Physiol Biochem.

[38] Ito R, Masukawa Y, Hoshino T (2013). Purification, kinetics, inhibitors and CD for recombinant β-amyrin synthase from Euphorbia tirucalli L and functional analysis of the DCTA motif, which is highly conserved among oxidosqualene cyclases. FEBS J.

[39] Vishwakarma RK, Sonawane P, Singh S, Kumari U, Ruby, Khan BM (2013). Molecular characterization and differential expression studies of an oxidosqualene cyclase (OSC) gene of Brahmi (Bacopa monniera). Physiol Mol Biol Plants.

[40] Fukushima EO, Seki H, Ohyama K, Ono E, Umemoto N, Mizutani M, Saito K, Muranaka T (2011). CYP716A subfamily members are multifunctional oxidases in triterpenoid biosynthesis. Plant Cell Physiol.

[41] Suzuki H, Fukushima EO, Umemoto N, Ohyama K, Seki H, Muranaka T (2018). Comparative analysis of CYP716A subfamily enzymes for the heterologous production of C-28 oxidized triterpenoids in transgenic yeast. Plant Biotechnol (Tokyo, Japan).

[42] Liu N, Zhang L (2002). Identification of two new cytochrome P450 genes and their 5′-flanking regions from the housefly, Musca domestica. Insect Biochem Mol Biol.

[43] Xue L, He Z, Bi X, Xu W, Wei T, Wu S, Hu S (2019). Transcriptomic profiling reveals MEP pathway contributing to ginsenoside biosynthesis in Panax ginseng. BMC Genomics.

[44] Kim YJ, Zhang D, Yang DC (2015). Biosynthesis and biotechnological production of ginsenosides. Biotechnol Adv.

[45] Wang WF, Xiao H, Zhong JJ (2018). Biosynthesis of a ganoderic acid in Saccharomyces cerevisiae by expressing a cytochrome P450 gene from Ganoderma lucidum. Biotechnol Bioeng.

[46] Wang J, Li J, Li J, Li J, Liu S, Gao W (2017). LSP1, a responsive protein from Meyerozyma guilliermondii, elicits defence response and improves glycyrrhizic acid biosynthesis in Glycyrrhiza uralensis Fisch adventitious roots. J Cell Physiol.

[47] Nguyen KV, Pongkitwitoon B, Pathomwichaiwat T, Viboonjun U, Prathanturarug S (2019). Effects of methyl jasmonate on the growth and triterpenoid production of diploid and tetraploid Centella asiatica (L.) Urb. Hairy root cultures. Sci Rep.

[48] Fu J, Liu G, Yang M, Wang X, Chen X, Chen F, Yang Y (2019). Isolation and functional analysis of squalene synthase gene in tea plant Camellia sinensis. Plant Physiol Biochem.

[49] Vergara Martínez VM, Estrada-Soto SE, Arellano-García JJ, Rivera-Leyva JC, Castillo-España P, Flores AF, Cardoso-Taketa AT, Perea-Arango I (2018). Methyl Jasmonate and salicylic acid enhanced the production of Ursolic and Oleanolic acid in callus cultures of Lepechinia Caulescens. Pharmacogn Mag.

[50] Yi GE, Robin AH, Yang K, Park JI, Hwang BH, Nou IS. Exogenous methyl Jasmonate and salicylic acid induce subspecies-specific patterns of Glucosinolate accumulation and gene expression in *Brassica oleracea* L. Molecules (Basel, Switzerland). 2016;21(10). 10.3390/molecules21101417 PMC6273115 27783045

[51] Cappellari LDR, Santoro MV, Schmidt A, Gershenzon J, Banchio E. Improving Phenolic Total Content and Monoterpene in *Mentha x piperita* by Using Salicylic Acid or Methyl Jasmonate Combined with Rhizobacteria Inoculation. Int J Mol Sci. 2019;21(1). 10.3390/ijms21010050 PMC6981552 31861733

[52] Bontpart T, Cheynier V, Ageorges A, Terrier N (2015). BAHD or SCPL acyltransferase? What a dilemma for acylation in the world of plant phenolic compounds. N Phytol.

[53] Liu YY, Mo T, Wang XH, Shi SP, Liu X, Tu PF (2016). Research progress of plant BAHD acyltransferase family. Zhongguo Zhong Yao Za Zhi.

[54] Molina I, Kosma D (2015). Role of HXXXD-motif/BAHD acyltransferases in the biosynthesis of extracellular lipids. Plant Cell Rep.

[55] Morales-Quintana L, Moya-León MA, Herrera R (2015). Computational study enlightens the structural role of the alcohol acyltransferase DFGWG motif. J Mol Model.

