# Supplementary material for: Metabolic stimulation-elicited transcriptional responses and biosynthesis of acylated triterpenoids precursors in the medicinal plant Helicteres angustifolia
Source: BMC Plant Biol. 2022 Feb 25;22:86. doi: 10.1186/s12870-022-03429-8 (PMC8876399; doi:10.1186/s12870-022-03429-8)
Supplement: Supplementary file 1 — Additional file 1: Figure S1. The electrophoresis diagrams of RNA extracted from different treatment groups of H. angustifolia tree leaves. [file 12870_2022_3429_MOESM1_ESM.doc]

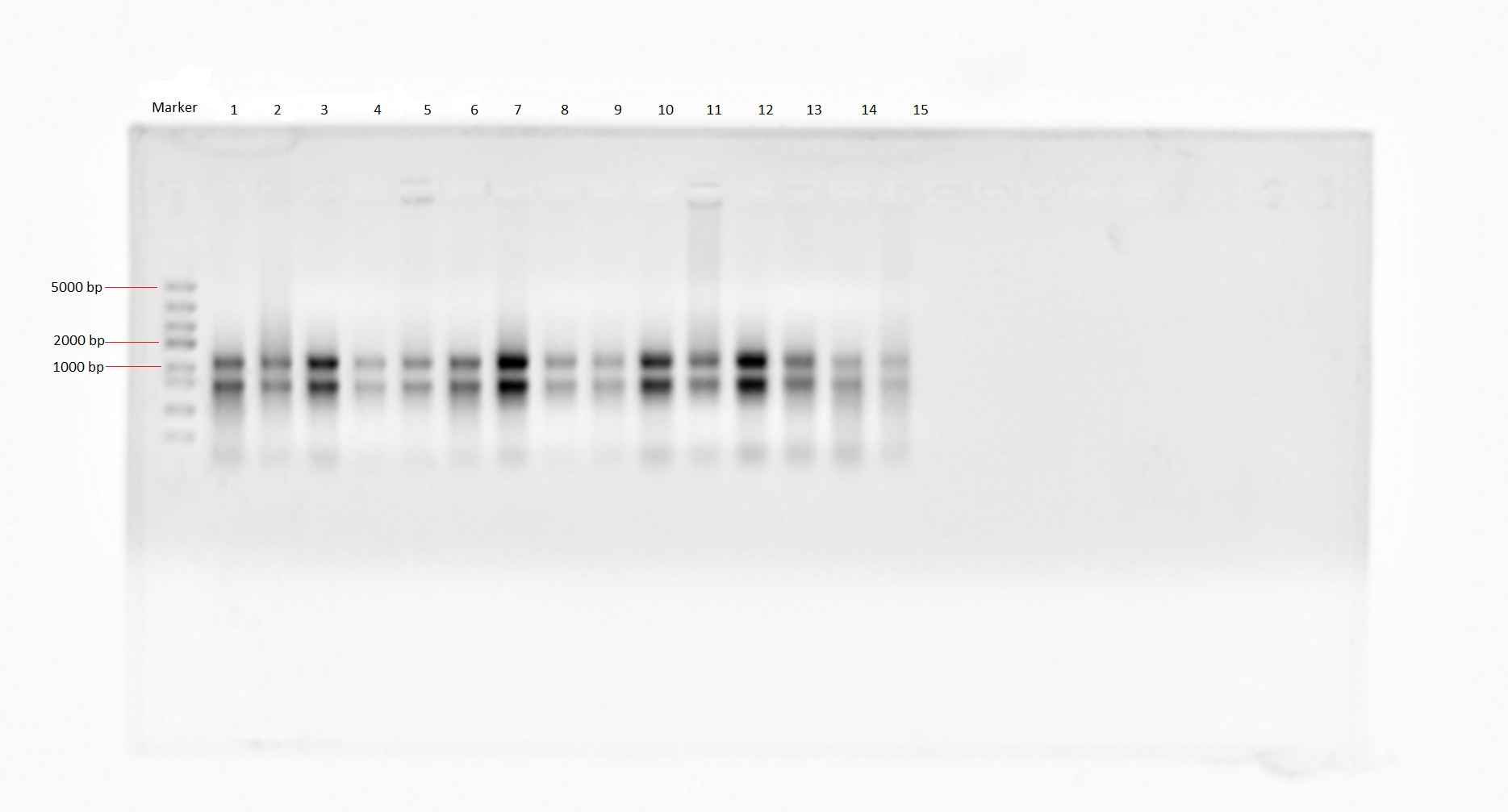


Figure.**S1** The electrophoresis diagrams of RNA extracted from different treatment groups of *H. Angustifolia* tree leaves. **1-3** Methyl jasmonate treatment group (MeJA); **4-6** Ethanol solvent treatment group (EtOH); **7-9** Salicylic acid treatment group (SA); **10-12** Mechanical damage group (MD); **13-15** Negative control group (NC).
