# Supplementary material for: Metabolic stimulation-elicited transcriptional responses and biosynthesis of acylated triterpenoids precursors in the medicinal plant Helicteres angustifolia
Source: BMC Plant Biol. 2022 Feb 25;22:86. doi: 10.1186/s12870-022-03429-8 (PMC8876399; doi:10.1186/s12870-022-03429-8)
Supplement: Supplementary file 2 — Additional file 2: Figure S2. Investigation of hormone concentration. [file 12870_2022_3429_MOESM2_ESM.doc]

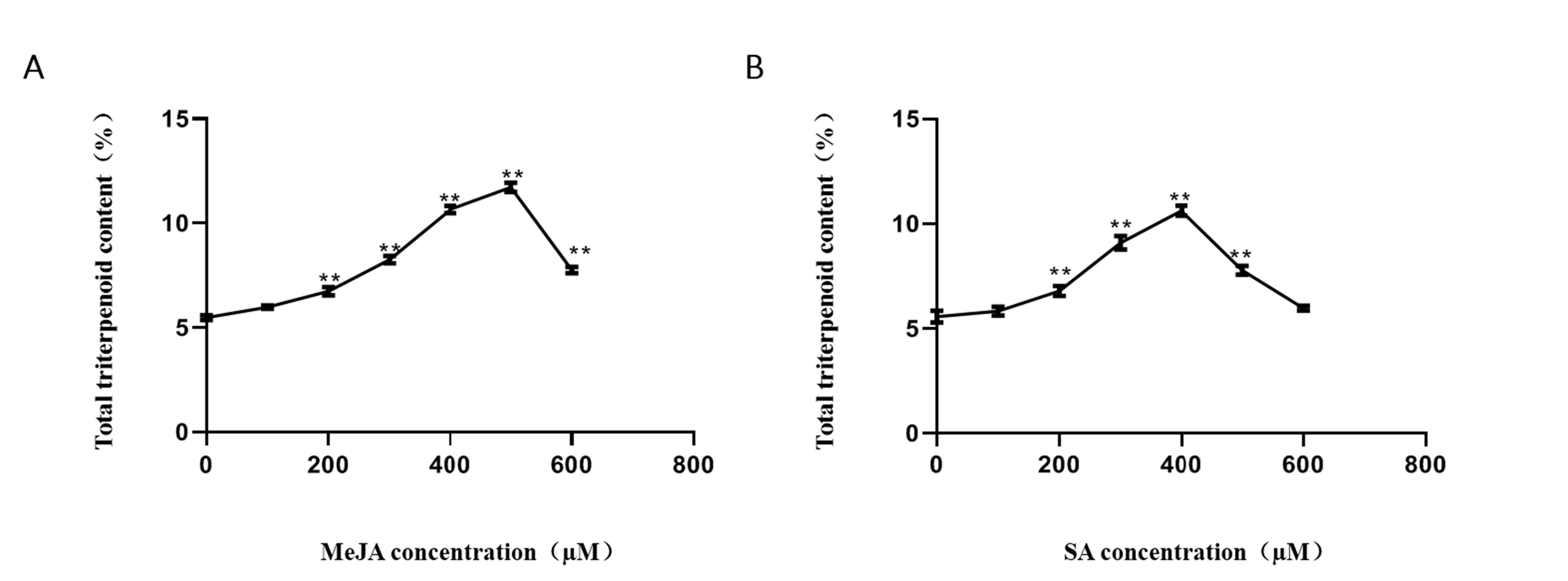


**Figure.S2** Investigation of hormone concentration. **A** Total triterpenoids content under treatments of methyl jasmonate with different concentrations; **B** Total triterpenoids content under treatments of salicylic acid with different concentrations. 0 µM hormone treatment group as control, ** indicates P< 0.01.
