# Supplementary material for: Metabolic stimulation-elicited transcriptional responses and biosynthesis of acylated triterpenoids precursors in the medicinal plant Helicteres angustifolia
Source: BMC Plant Biol. 2022 Feb 25;22:86. doi: 10.1186/s12870-022-03429-8 (PMC8876399; doi:10.1186/s12870-022-03429-8)
Supplement: Supplementary file 3 — Additional file 3: Figure S3. Comparisons of the total triterpenoid content among different treatment groups. [file 12870_2022_3429_MOESM3_ESM.doc]

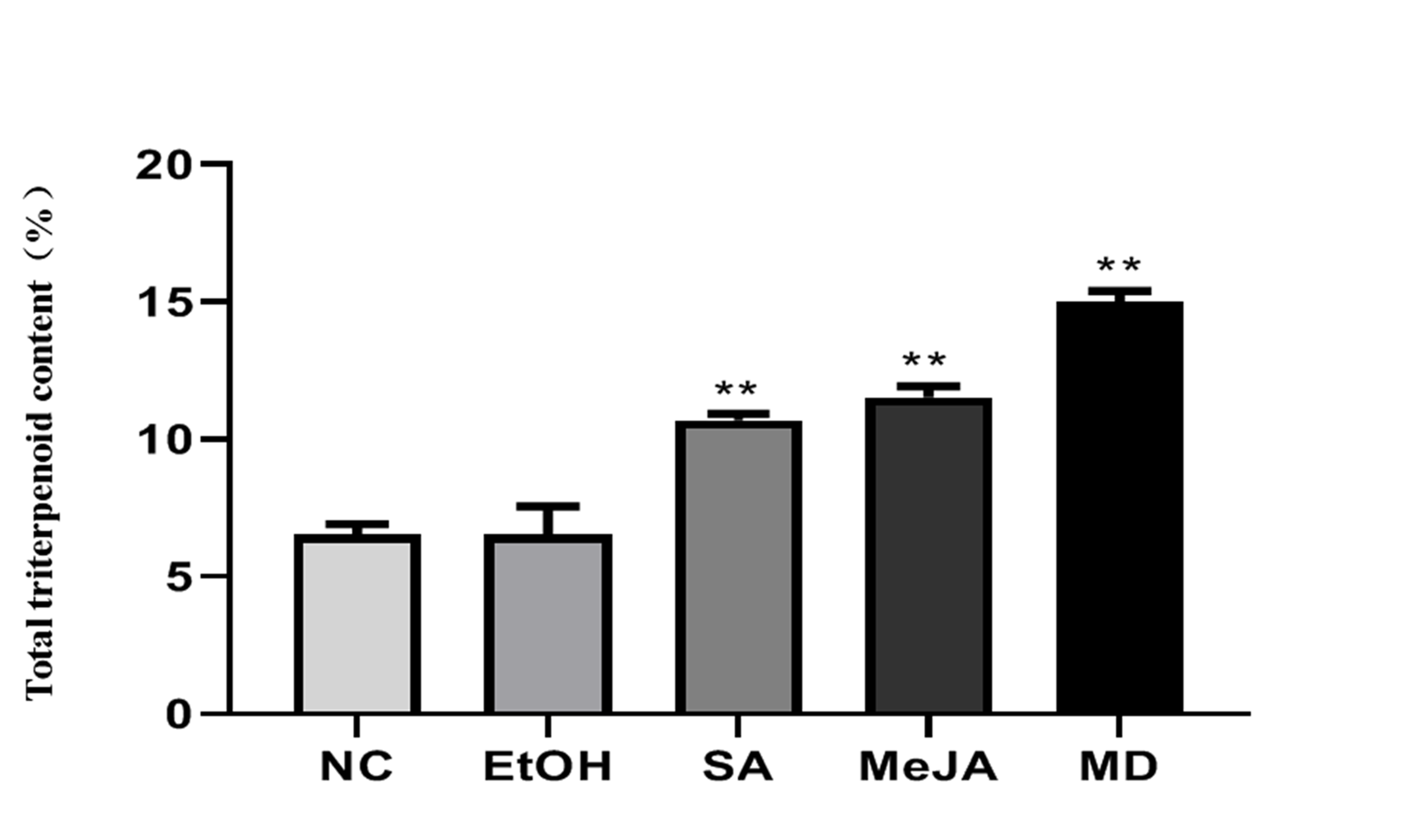


**Figure.S3** Comparisons of the total triterpenoid content among different treatment groups. **indicated p<0.01 when compared with the NC group.
