# Supplementary figures and images for: Metabolic stimulation-elicited transcriptional responses and biosynthesis of acylated triterpenoids precursors in the medicinal plant Helicteres angustifolia
Source: BMC Plant Biol. 2022 Feb 25;22:86. doi: 10.1186/s12870-022-03429-8 (PMC8876399; doi:10.1186/s12870-022-03429-8)

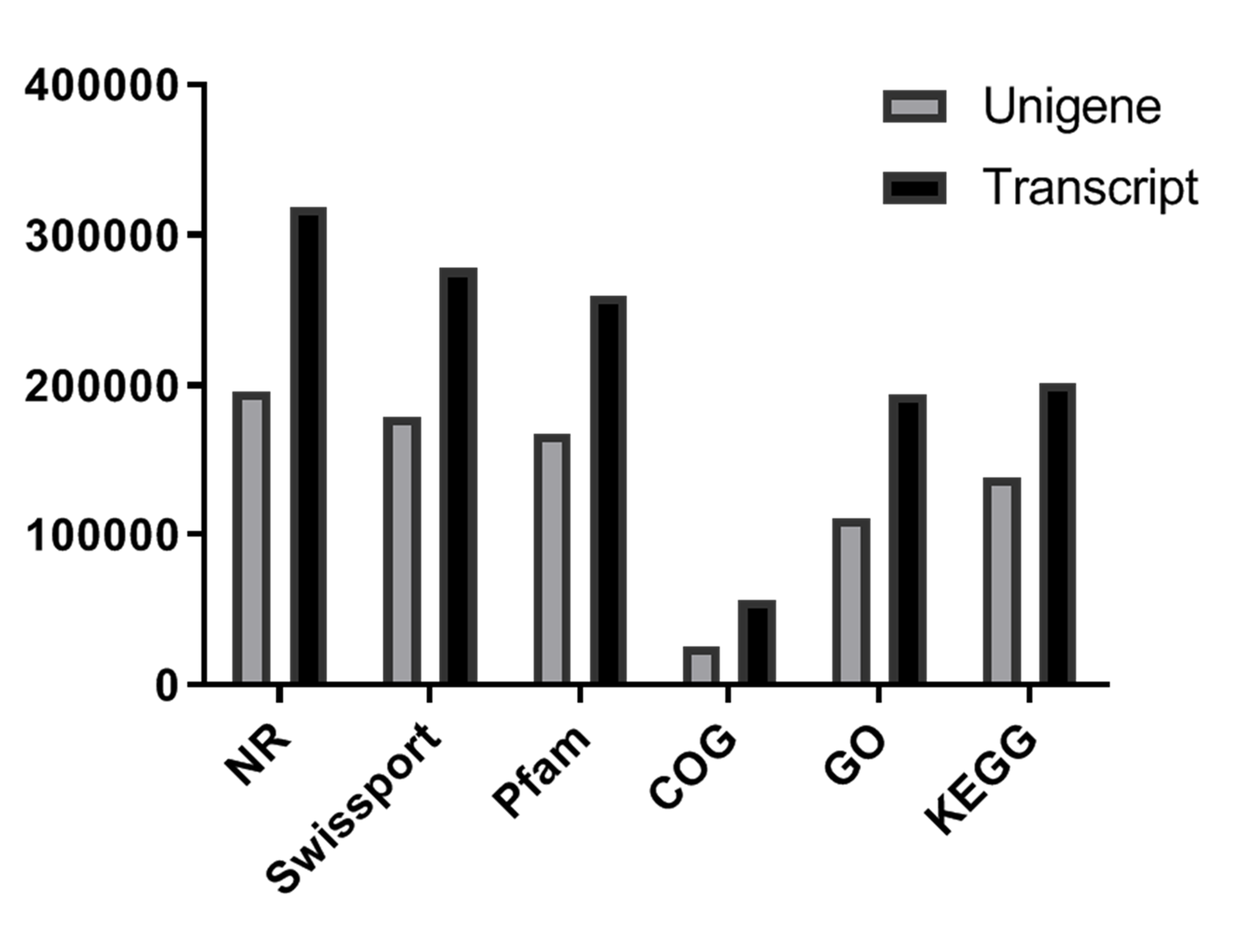


**Figure.S4** Functional annotation of the transcripts and unigenes against six databases.

Supplement: Supplementary file 4 — Additional file 4: Figure S4. Functional annotation of the transcripts and unigenes against six databases. [file 12870_2022_3429_MOESM4_ESM.doc]

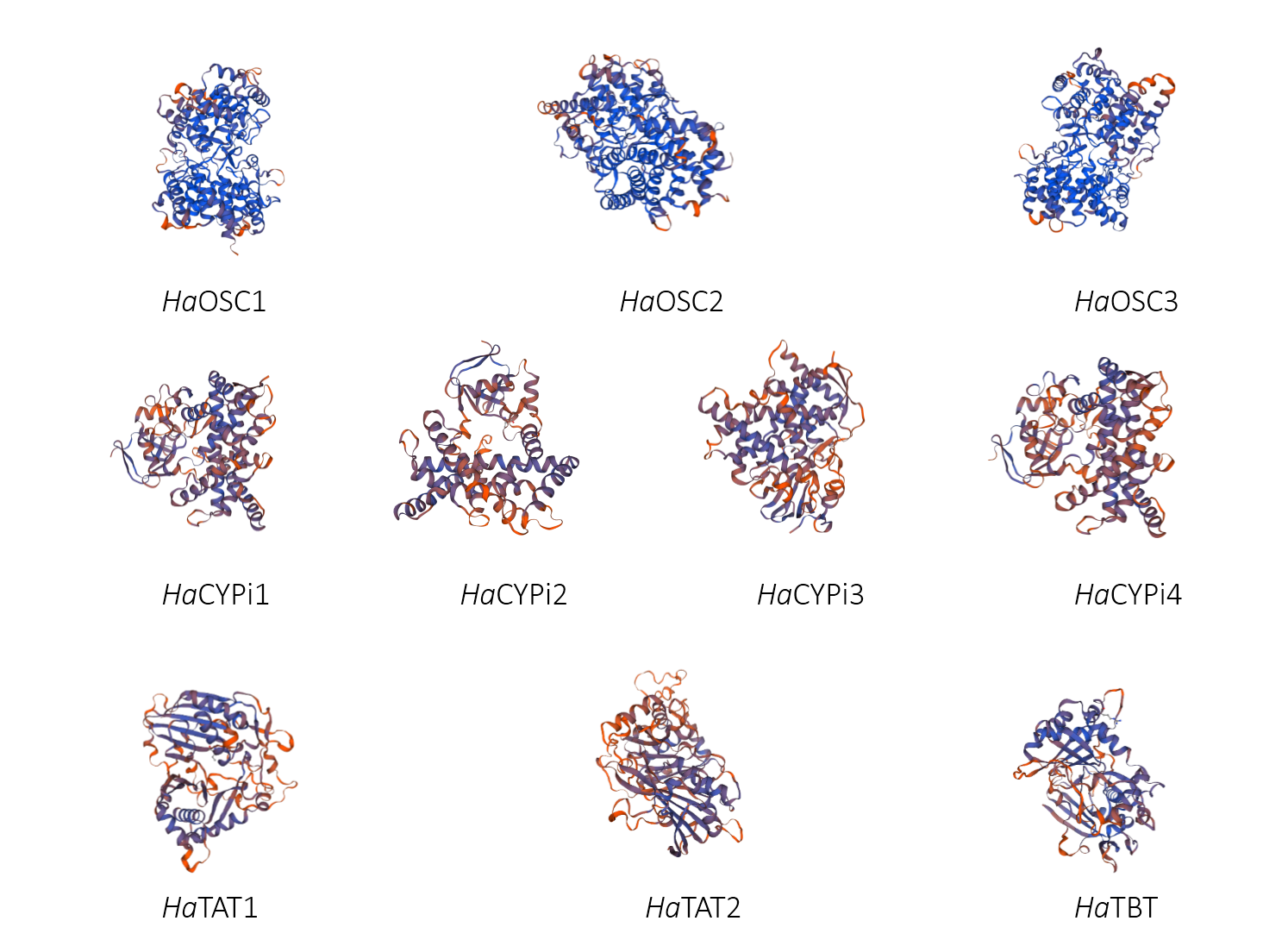


Figure.**S8** Three dimention structure of target gene coding proteins.

Supplement: Supplementary file 8 — Additional file 8: Figure S8. Three dimention structure of target gene coding proteins. [file 12870_2022_3429_MOESM8_ESM.doc]

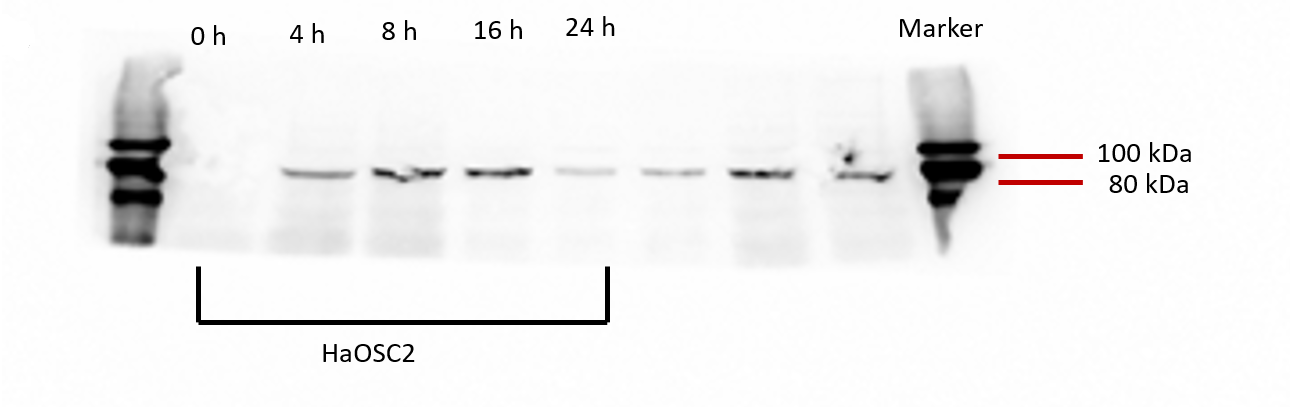


Figure.**S9** Protein expression analysis ofHaOSC2

Supplement: Supplementary file 9 — Additional file 9: Figure S9. Protein expression analysis of HaOSC2. [file 12870_2022_3429_MOESM9_ESM.doc]
