# Supplementary material for: Metabolic stimulation-elicited transcriptional responses and biosynthesis of acylated triterpenoids precursors in the medicinal plant Helicteres angustifolia
Source: BMC Plant Biol. 2022 Feb 25;22:86. doi: 10.1186/s12870-022-03429-8 (PMC8876399; doi:10.1186/s12870-022-03429-8)
Supplement: Supplementary file 5 — Additional file 5: Figure S5. Venn diagram indicating the numbers of common and specific expressed unigenes among six databases. [file 12870_2022_3429_MOESM5_ESM.doc]

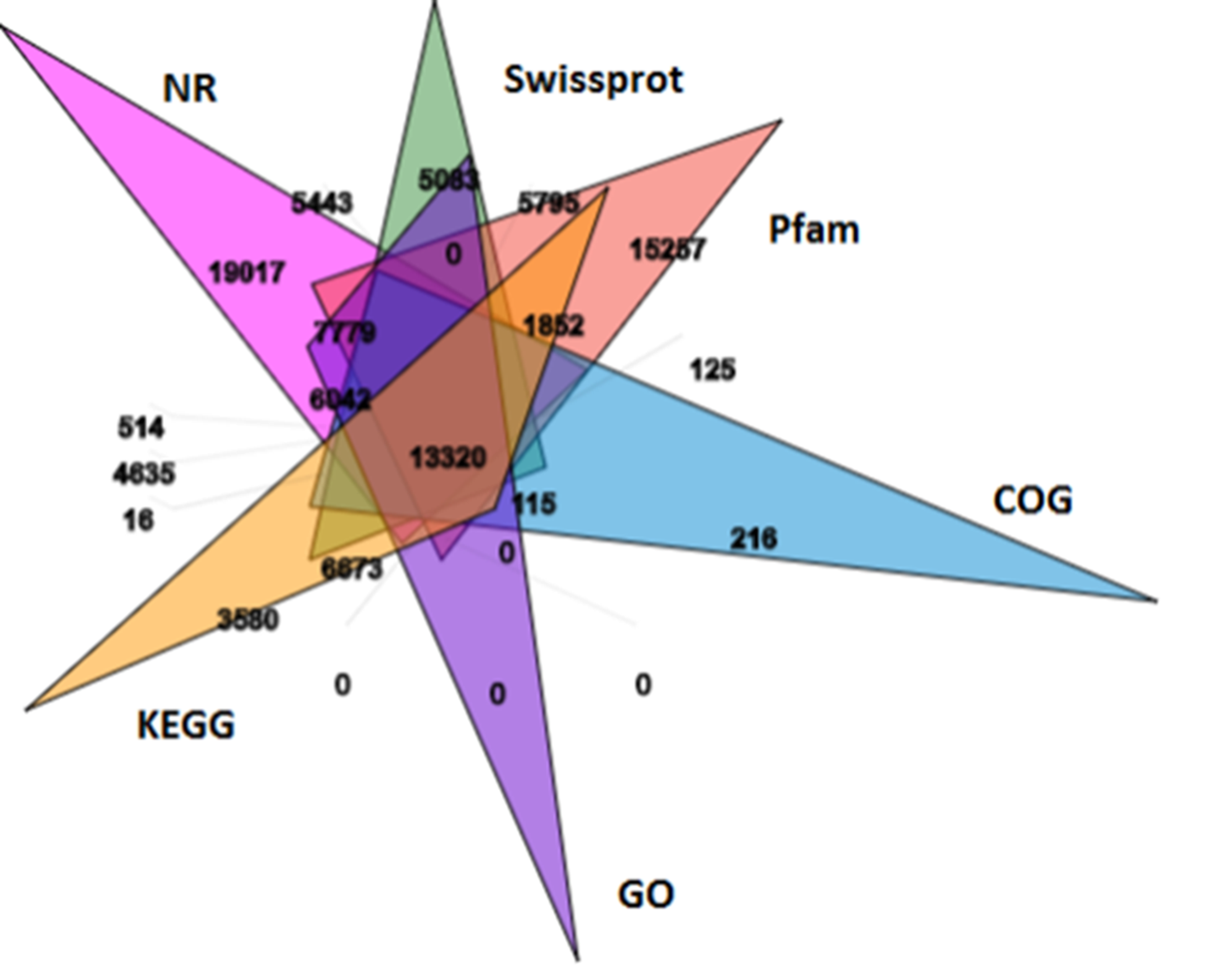


**Figure.S5** Venn diagram indicating the numbers of common and specific expressed unigenes among six databases.
