# Supplementary material for: Metabolic stimulation-elicited transcriptional responses and biosynthesis of acylated triterpenoids precursors in the medicinal plant Helicteres angustifolia
Source: BMC Plant Biol. 2022 Feb 25;22:86. doi: 10.1186/s12870-022-03429-8 (PMC8876399; doi:10.1186/s12870-022-03429-8)
Supplement: Supplementary file 6 — Additional file 6: Figure S6. KEGG enrichment results of the DEGs among negative control group and different treatment groups. [file 12870_2022_3429_MOESM6_ESM.doc]

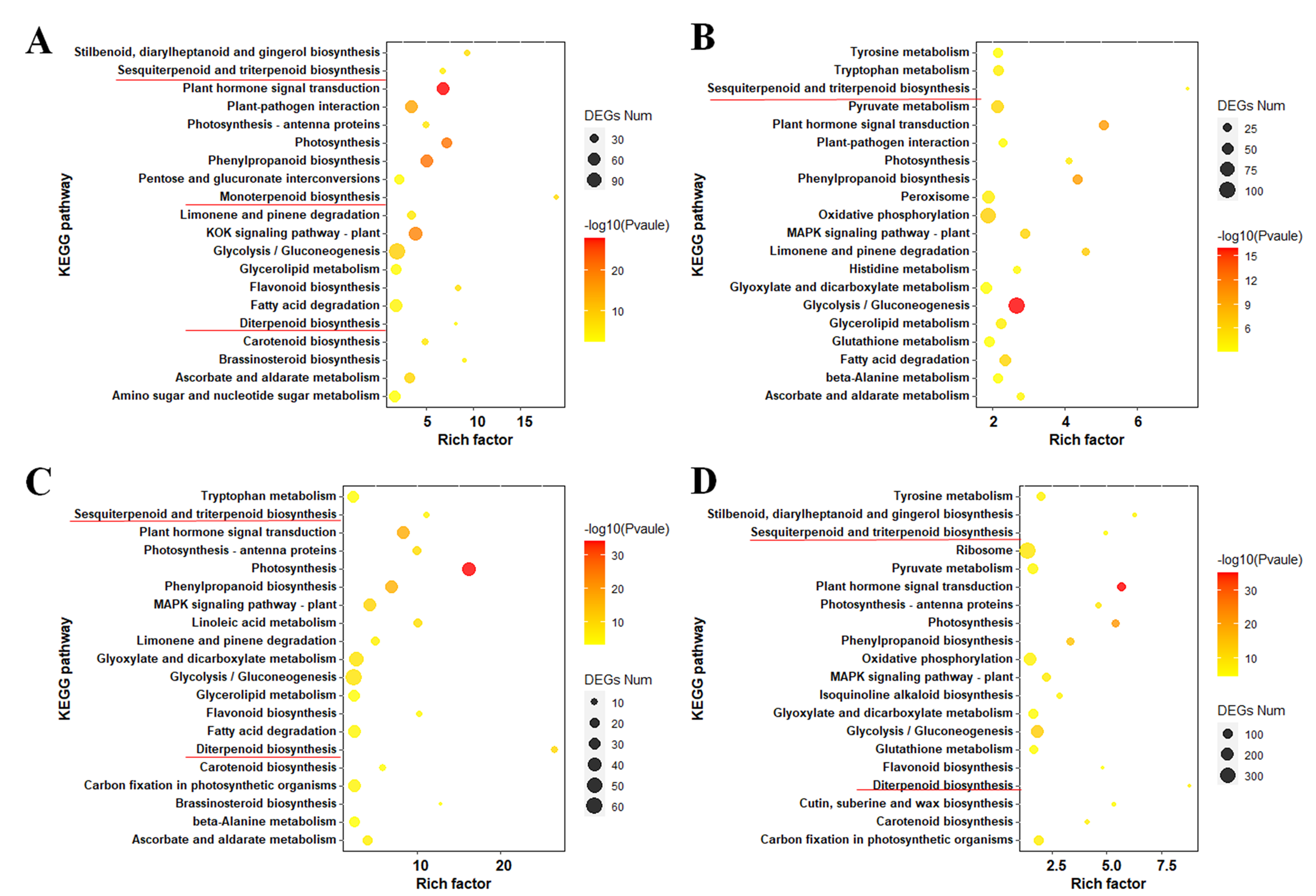


**Figure.S6** KEGG enrichment results of the DEGs among negative control group and different treatment groups. A KEGG enrichment situation of DEGs under the comparisons of NC with EtOH; B KEGG enrichment situation of DEGs under the comparisons of NC with SA; C KEGG enrichment situation of DEGs under the comparisons of NC with MeJA; D KEGG enrichment situation of DEGs under the comparisons of NC with MD.
