# Supplementary material for: Metabolic stimulation-elicited transcriptional responses and biosynthesis of acylated triterpenoids precursors in the medicinal plant Helicteres angustifolia
Source: BMC Plant Biol. 2022 Feb 25;22:86. doi: 10.1186/s12870-022-03429-8 (PMC8876399; doi:10.1186/s12870-022-03429-8)
Supplement: Supplementary file 7 — Additional file 7: Figure S7. The expression pattern of key related genes involved with triterpenoids biosynthesis were shown using a heatmap. [file 12870_2022_3429_MOESM7_ESM.doc]

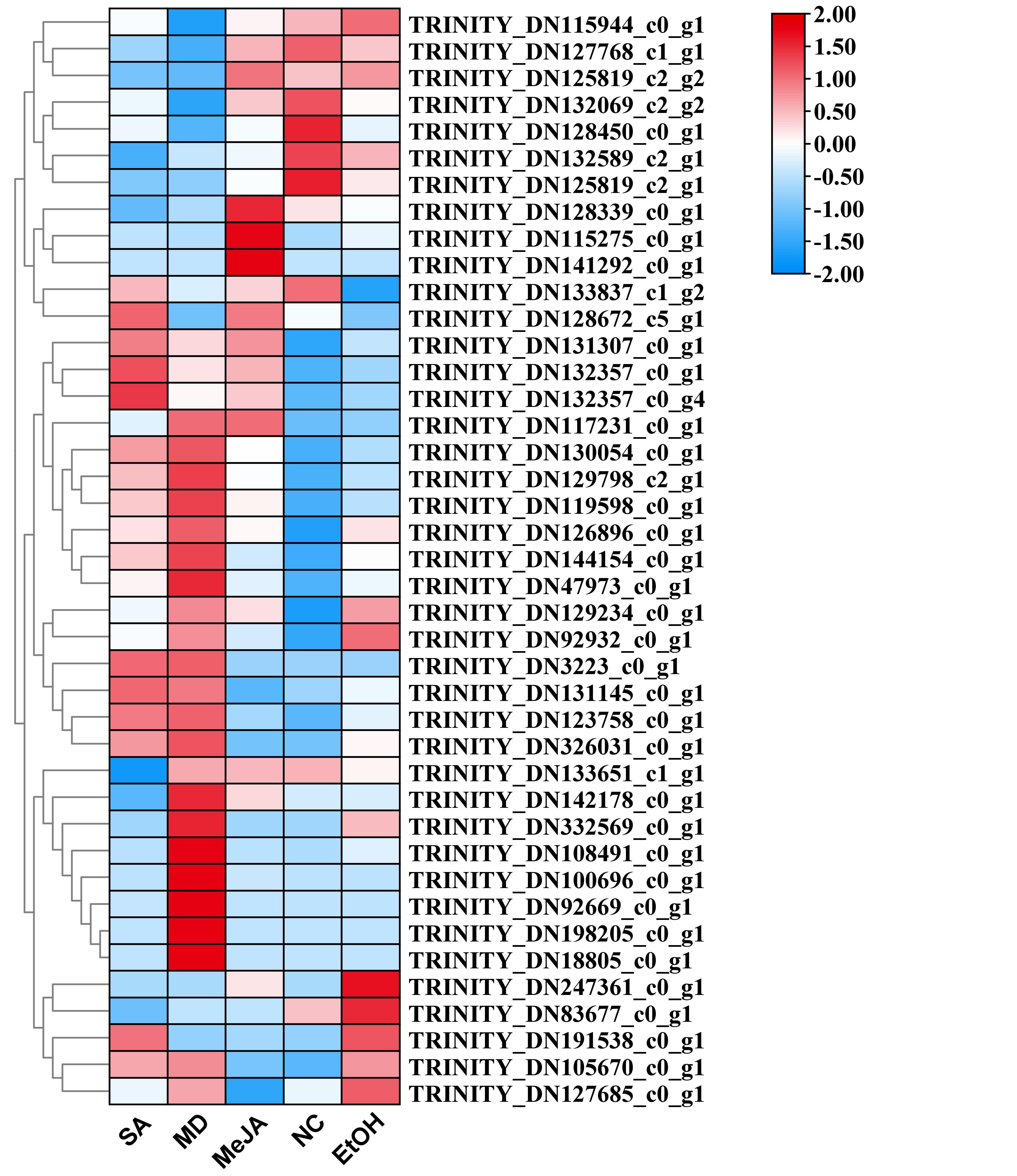


Figure.**S7** The expression pattern of key related genes involved with triterpenoids biosynthesis were shown using a heatmap.
