# Supplementary material for: Metabolic stimulation-elicited transcriptional responses and biosynthesis of acylated triterpenoids precursors in the medicinal plant Helicteres angustifolia
Source: BMC Plant Biol. 2022 Feb 25;22:86. doi: 10.1186/s12870-022-03429-8 (PMC8876399; doi:10.1186/s12870-022-03429-8)
Supplement: Supplementary file 10 — Additional file 10: Figure S10. Analysis of amino acid sequences of TATs (Triterpenoid acetyltransferase) and TBT (Triterpenoid benzoyl transferase). [file 12870_2022_3429_MOESM10_ESM.doc]

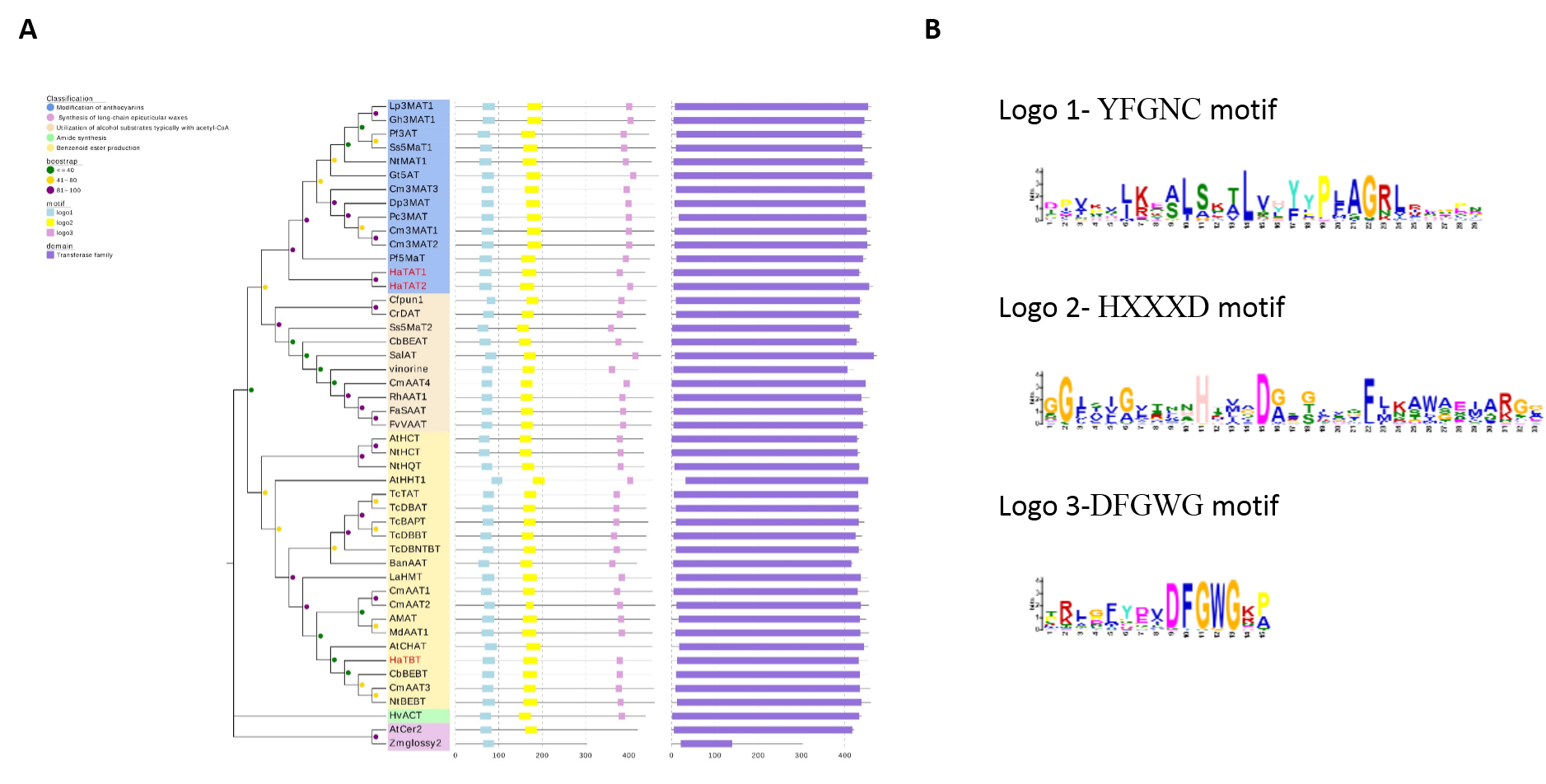


Figure.**S10** Analysis of amino acid sequences of TATs (Triterpenoid acetyltransferase) and TBT (Triterpenoid benzoyl transferase). A, Phylogenetic tree, conserved domain and conserved motif analysis. B, Conservative motifs corresponding sequences.
