# Supplementary material for: Metabolic stimulation-elicited transcriptional responses and biosynthesis of acylated triterpenoids precursors in the medicinal plant Helicteres angustifolia
Source: BMC Plant Biol. 2022 Feb 25;22:86. doi: 10.1186/s12870-022-03429-8 (PMC8876399; doi:10.1186/s12870-022-03429-8)
Supplement: Supplementary file 11 — Additional file 11: Figure S11. SDS-PAGE analysis. [file 12870_2022_3429_MOESM11_ESM.doc]

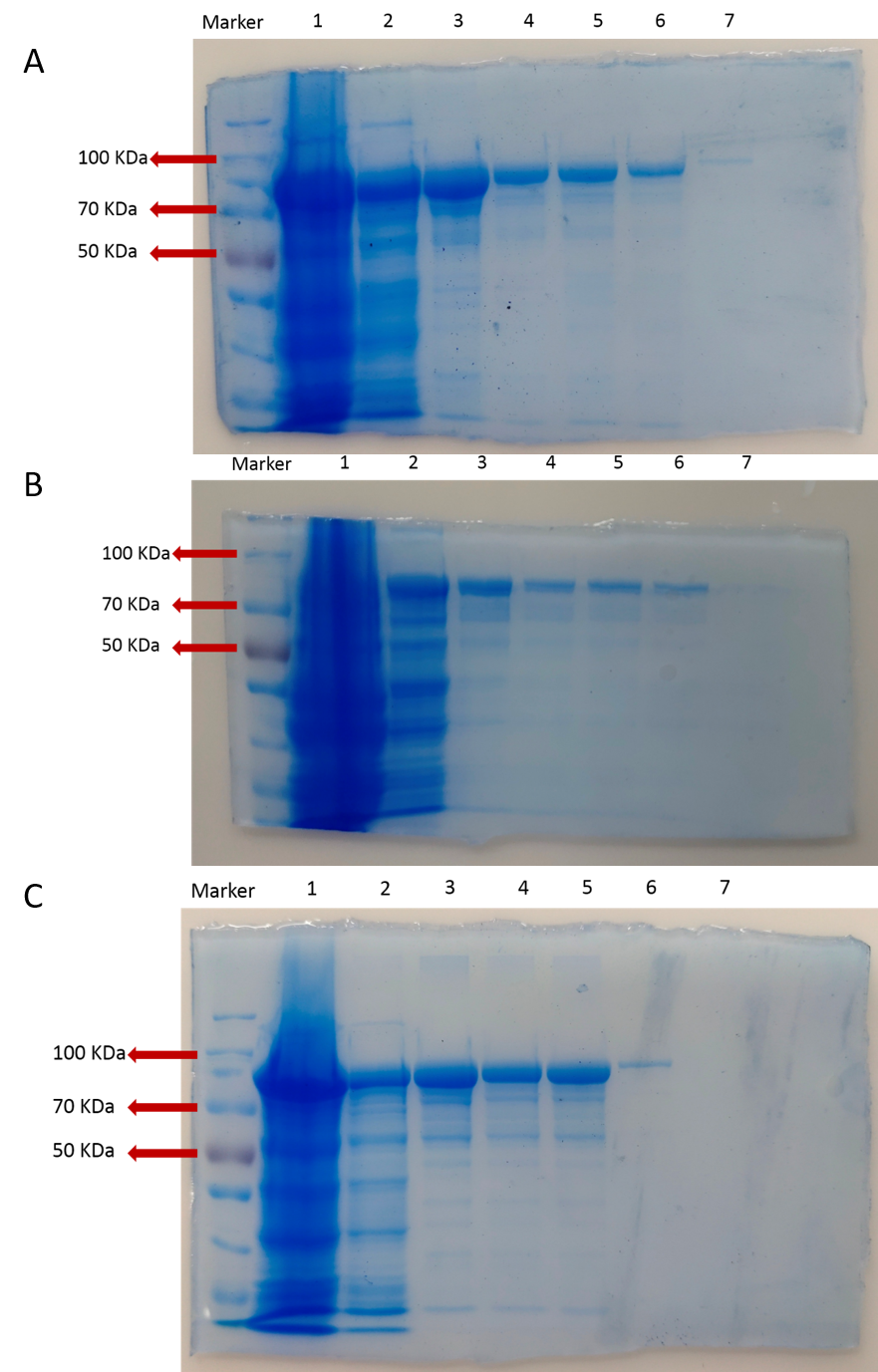


Figure.**S11** SDS-PAGE analysis. A: SDS-PAGE analysis of HaTAT1 protein; B: SDS-PAGE analysis of HaTAT2 protein; C: SDS-PAGE analysis of HaTBT protein. 1 Total protein after induction; 2 Soluble protein; 3-7 Purified recombinant protein from first collection tube to fifth collection tube.
