# Supplementary material for: Metabolic stimulation-elicited transcriptional responses and biosynthesis of acylated triterpenoids precursors in the medicinal plant Helicteres angustifolia
Source: BMC Plant Biol. 2022 Feb 25;22:86. doi: 10.1186/s12870-022-03429-8 (PMC8876399; doi:10.1186/s12870-022-03429-8)
Supplement: Supplementary file 12 — Additional file 12: Table S1. Primers for qRT-PCR. [file 12870_2022_3429_MOESM12_ESM.doc]

Table S1 Primers for qRT-PCR

| Primer | Sequence |
| --- | --- |
| qHaα-TUB-F | CCGTGCTCTTGGACAATGAAG |
| qHaα-TUB-R | TGGTTTGGAACTCGGTGATATC |
| qHaβ-TUB-F | TTCTCCCGCATTGACCAT |
| qHaβ-TUB-R | CCGACTTCCTCATAATCTTTCT |
| qHaActin-F | GGTGAAGTTGATGCCGTAGC |
| qHaActin-R | GGATAGAACAAACCCATAGGAGG |
| qHaAACT-F | TAGTTGACAAGGATGAAGGTTTAGG |
| qHaAACT-R | CTCCACTCACTAAAACTAAAGCAGCAG |
| qHaHMGR -F | CCTTGGATGGATTTGATTACGAG |
| qHaHMGR -R | GAATAGCTTTACAGCCCCTATTAG |
| qHaDXS -F | CATCGGTGGTTTTGGTTCCC |
| qHaDXS -R | GCAGCAATGTGAGATGGTGTAAG |
| qHaOSC1 -F | GAGAGCTACAAATCTTGCCCTGAC |
| qHaOSC1-R | TGTCCAGCGTGAATCAAACCTAT |
| qHaOSC2-F | TTGGGAGCCAGTAAGAGGACC |
| qHaOSC2-R | ATTGAACCGCTTTAGCCACAG |
| qHaCYP-F | TGTGGCATTTGGTGGAGGTC |
| qHaCYP-R | TAGGCATGGGGTCAACGATT |
| qHaTAT1 -F | GTCATCAGTAGTTCGCCTCGGT |
| qHaTAT1 -R | CAATACTTCCTTCTTCTGCTCCAC |
| qHaTAT2 -F | CCGACTTACGCTCAAGCCAGT |
| qHaTAT1 -R | GAACAAACGAGGTTTCATCTCCC |
| qHaTBT-F | GATGTTCGCCTTATCTGTACCTTAAC |
| qHaTBT -R | GGATTCTTGCATAGTTCTCCTGC |
