# Supplementary material for: Metabolic stimulation-elicited transcriptional responses and biosynthesis of acylated triterpenoids precursors in the medicinal plant Helicteres angustifolia
Source: BMC Plant Biol. 2022 Feb 25;22:86. doi: 10.1186/s12870-022-03429-8 (PMC8876399; doi:10.1186/s12870-022-03429-8)
Supplement: Supplementary file 13 — Additional file 13: Table S2. Primers for amplification of ten putative genes. [file 12870_2022_3429_MOESM13_ESM.doc]

Table S2 Primers for amplification of ten putative genes

| Primer | Sequence |
| --- | --- |
| *HaOSC1*-F | ATGTGGAAGCTGAAGATAGCAAG |
| *HaOSC1*-R | TTAAGCAATAAGGAGGATACGCCGT |
| *Ha*OSC2-F | TCTTATAATCCTCCTGGGATCACCA |
| *Ha*OSC2-R | CACATACTTCAGTGACCACACAACC |
| *Ha*OSC3-F | CTAAGTCAAAGATGTGGAAACTGG |
| *Ha*OSC3-R | GGAAACTAAGGGAGCTGAGATAATC |
| *HaCYPi1*-F | ATGGAGCATTTCTACCTTCCCCTTC |
| *HaCYPi1*-R | TTATTTATATATGTTGGGTGAGTGGTGAGG |
| *HaCYPi2*-F | ATGGAGCATTTCTACCTTCCCCTTC |
| *HaCYPi2*-R | TCAAGATAATTGAAAAAGTAAAGTT |
| *HaCYPi3*-F | ATGGAGTTGTCTTCCCTATGTGGTG |
| *HaCYPi3*-R | CTACGCAACTAAATGAGGAAAGAGG |
| *HaCYPi4*-F | ATGGAGTTGTTTTTTCTTTGTGGTC |
| *HaCYPi4*-R | CTATGCAGTTGTTTGAGGAATGAGG |
| *HaTAT1*-F | ATGATGAAAGGTGTTCGATGTATCT |
| *HaTAT1*-R | TTAAACCGTGACTGTATCCATG |
| *HaTAT2*-F | ATGCCTTCTTCTTCAGTTACTCTTG |
| *HaTAT2*-R | TCACACCATTTCTGATACGTACTGC |
| *HaTBT*-F | ATGGCATTGCTACCAACCAATACTC |
| *HaTBT*-R | TTAAAGTGAATTTCTAATGAAC |
