# Supplementary material for: Metabolic stimulation-elicited transcriptional responses and biosynthesis of acylated triterpenoids precursors in the medicinal plant Helicteres angustifolia
Source: BMC Plant Biol. 2022 Feb 25;22:86. doi: 10.1186/s12870-022-03429-8 (PMC8876399; doi:10.1186/s12870-022-03429-8)
Supplement: Supplementary file 14 — Additional file 14: Table S3. Gene-specific forward and reverse primers. [file 12870_2022_3429_MOESM14_ESM.doc]

Table S3 Gene-specific forward and reverse primers

| Primer | Sequence |
| --- | --- |
| pESC-TRP-*HaOSC1*F | aatttttgaaaattcgaattcATGTGGAAGCTGAAGATAGCAGAA |
| pESC-TRP-Ha*OSC1*R | agaattgttaattaagagctcTCAATGATGATGATGATGATGTATGGATTGGACCAAATTGGC |
| pESC-TRP-*HaOSC2*F | gaatttttgaaaattcgaattcATGTGGAAGCTTAAGATTGCAGAG |
| pESC-TRP-*HaOSC2*R | ggcgaagaattgttaattaagagctcTCAATGATGATGATGATGATGAACCATTTTTGCA |
| pESC-TRP-*HaOSC3*F | gaatttttgaaaattcgaattcATGTGGAAACTGGAGATAGCAGAA |
| pESC-TRP-*HaOSC3*R | ggcgaagaattgttaattaagagctcTTAATGATGATGATGATGATGTGAAGGTGAGAGCACATGTTTATA |
| pESC-TRP-*HaCYPi1*F | ctatagggcccgggcgtcgacATGGCGTCAACCTTGGAGACGG |
| pESC-TRP-HaCYPi1R | ttagagcggatcttagctagcTCAATGATGATGATGATGATGAATGACGCTTCCCATATTG |
| pESC-TRP-*HaCYPi2*F | ctatagggcccgggcgtcgacATGTGTGTCCTCACAACTACACCCTT |
| pESC-TRP-*HaCYPi2*R | ttagagcggatcttagctagcTTAATGATGATGATGATGATGTACATTATTAATTGGTATGGGCTCCACCACCA |
| pESC-TRP-*HaCYPi3*F | ctatagggcccgggcgtcgacATGGCTACAAAAGTAGTCAGGCCC |
| pESC-TRP-*HaCYPi3*R | ttagagcggatcttagctagcTTAATGATGATGATGATGATGTTTGACAATCAGTGGAATGGGTCGAAC |
| pESC-TRP-*HaCYPi4*F | ctatagggcccgggcgtcgacATGGAGCCTGAGGCTTCGAGGAG |
| pESC-TRP-*HaCYPi4*R | ttagagcggatcttagctagcTTAATGATGATGATGATGATGTTTCAACCACGGGTGGAATGGG |
| pCOLD-TF -*HaTAT1*F | gaaggtaggcatatggagctcATGATGAAAGGTGTTCGATGTATCTC |
| pCOLD-TF-*HaTAT1*R | caggtcgacaagcttgaattcTTAAACCGTGACTGTATCCATGAAT |
| pCOLD-TF–*HaTAT2*F | gaaggtaggcatatggagctcATGCCTTCTTCTTCAGTTACTCTTGTT |
| pCOLD-TF-*HaTAT2*R | caggtcgacaagcttgaattcTCACACCATTTCTGATACGTACTGC |
| pCOLD-TF-*HaTBT*F | gaaggtaggcatatggagctcATGGCATTGCTACCAACCAATAC |
| pCOLD-TF-*HaTBT*R | caggtcgacaagcttgaattcTTAAAGTGAATTTCTAATGAACAACTTCTT |
