# Supplementary material for: Metabolic stimulation-elicited transcriptional responses and biosynthesis of acylated triterpenoids precursors in the medicinal plant Helicteres angustifolia
Source: BMC Plant Biol. 2022 Feb 25;22:86. doi: 10.1186/s12870-022-03429-8 (PMC8876399; doi:10.1186/s12870-022-03429-8)
Supplement: Supplementary file 15 — Additional file 15: Table S4. Effect of different methyl jasmonate and salicylic acid concentrations and on total triterpenoids content. [file 12870_2022_3429_MOESM15_ESM.doc]

Table S4 Effect of different methyl jasmonate and salicylic acid concentrations and on total triterpenoids content

| groups | number | total triterpenoids content（%） |
| --- | --- | --- |
| 0 µM MeJA | 3 | 5.487±0.118 |
| 100 µM MeJA | 3 | 5.980±0.079 |
| 200 µM MeJA | 3 | 6.747±0.197** |
| 300 µM MeJA | 3 | 8.257±0.180** |
| 400 µM MeJA | 3 | 10.660±0.173** |
| 500 µM MeJA | 3 | 11.713±0.229** |
| 600 µM MeJA | 3 | 7.756±0.153** |
| P | - | 0.000 |
| 0 µM SA | 3 | 5.565±0.283 |
| 100 µM SA | 3 | 5.830±0.215 |
| 200 µM SA | 3 | 6.799±0.247** |
| 300 µM SA | 3 | 9.093±0.328** |
| 400 µM SA | 3 | 10.635±0.236** |
| 500 µM SA | 3 | 7.777±0.216** |
| 600 µM SA | 3 | 5.965±0.122 |
| P | - | 0.000 |

Bonferroni test：**P<0.01。
