# Supplementary material for: Metabolic stimulation-elicited transcriptional responses and biosynthesis of acylated triterpenoids precursors in the medicinal plant Helicteres angustifolia
Source: BMC Plant Biol. 2022 Feb 25;22:86. doi: 10.1186/s12870-022-03429-8 (PMC8876399; doi:10.1186/s12870-022-03429-8)
Supplement: Supplementary file 16 — Additional file 16: Table S5. Effects of different metabolic stimulation treatments on total triterpenoids content. [file 12870_2022_3429_MOESM16_ESM.docx]

Table S5 Effects of different metabolic stimulation treatments on total triterpenoids content

| groups | number | total triterpenoids content（%） |
| --- | --- | --- |
| NC | 3 | 6.521±0.385 |
| EtOH | 3 | 6.524±1.035 |
| SA | 3 | 10.691±0.235** |
| MeJA | 3 | 11.522±0.403** |
| MD | 3 | 15.015±0.380** |
| P | - | 0.000 |

Bonferroni test：**P<0.01。
