# Supplementary material for: Metabolic stimulation-elicited transcriptional responses and biosynthesis of acylated triterpenoids precursors in the medicinal plant Helicteres angustifolia
Source: BMC Plant Biol. 2022 Feb 25;22:86. doi: 10.1186/s12870-022-03429-8 (PMC8876399; doi:10.1186/s12870-022-03429-8)
Supplement: Supplementary file 17 — Additional file 17: Table S6. Transcriptome sequencing data of the H. angustifolia. [file 12870_2022_3429_MOESM17_ESM.doc]

Table S6 Transcriptome sequencing data of the *H. Angustifolia*

| Sample | Raw reads | Clean reads | Clean bases (Gb) | Q20 (%)^a^ | Q30 (%)^b^ | GC content (%) | Mapping ratio (%) |
| --- | --- | --- | --- | --- | --- | --- | --- |
| **SA_1** | 52,344,492 | 51,938,954 | 7.75 | 98.39 | 94.81 | 43.26 | 83.23 |
| **SA_2** | 56,735,686 | 56,280,986 | 8.38 | 98.50 | 95.09 | 43.65 | 85.26 |
| **SA_3** | 51,784,926 | 51,413,238 | 7.67 | 98.37 | 94.49 | 43.49 | 82.23 |
| **MD_1** | 56,595,726 | 56,236,366 | 8.39 | 98.55 | 95.24 | 43.61 | 83.98 |
| **MD_2** | 56,600,778 | 56,245,822 | 8.41 | 98.31 | 94.35 | 43.77 | 82.33 |
| **MD_3** | 62,467,350 | 62,079,418 | 9.27 | 98.35 | 94.47 | 43.84 | 81.96 |
| **MeJA_1** | 59,776,352 | 59,289,266 | 8.86 | 98.07 | 93.78 | 43.64 | 81.69 |
| **MeJA_2** | 52,029,606 | 51,593,104 | 7.68 | 98.42 | 94.91 | 43.66 | 83.70 |
| **MeJA_3** | 59,078,834 | 58,717,834 | 8.76 | 98.64 | 95.47 | 43.55 | 83.93 |
| **EtOH_1** | 55,635,250 | 55,229,936 | 8.23 | 98.56 | 95.27 | 43.55 | 83.79 |
| **EtOH_2** | 48,315,140 | 47,879,626 | 7.14 | 98.27 | 94.50 | 43.52 | 84.24 |
| **EtOH_3** | 51,310,980 | 51,003,308 | 7.61 | 98.57 | 95.29 | 43.65 | 85.05 |
| **NC_1** | 51,482,508 | 51,200,600 | 7.63 | 98.48 | 95.02 | 44.03 | 84.86 |
| **NC_2** | 60,175,340 | 59,808,842 | 8.92 | 98.56 | 95.25 | 44.01 | 85.73 |
| **NC_3** | 46,624,122 | 46,336,760 | 6.91 | 98.57 | 95.29 | 43.85 | 84.67 |
| **Summary** | 820,957,090 | 815,254,060 | 121.62 |  |  | 43.67 | 83.78 |

^a^Q20 indicates the percentage of bases with a Phred value >20，while ^b^Q30 indicates the percentage of bases with a Phred value > 30
