# Supplementary material for: Metabolic stimulation-elicited transcriptional responses and biosynthesis of acylated triterpenoids precursors in the medicinal plant Helicteres angustifolia
Source: BMC Plant Biol. 2022 Feb 25;22:86. doi: 10.1186/s12870-022-03429-8 (PMC8876399; doi:10.1186/s12870-022-03429-8)
Supplement: Supplementary file 18 — Additional file 18: Table S7. The overview statistics of annotation. [file 12870_2022_3429_MOESM18_ESM.doc]

Table S7 The overview statistics of annotation

| **Databases** | **Transcript number(percent)** | **Unigene number(percent)** |
| --- | --- | --- |
| **NR** | 318932(0.5481) | 196252(0.462) |
| **Swiss-Prot** | 277875(0.4775) | 179036(0.4214) |
| **Pfam** | 259796(0.4464) | 167947(0.3953) |
| **COG** | 56602(0.0973) | 25390(0.0598) |
| **GO** | 194591(0.3344) | 110706(0.2606) |
| **KEGG** | 201343(0.346) | 138066(0.325) |
| **Total annotation** | 373052(0.6411) | 245709(0.5784) |
| **Total number** | 581934(1) | 424824(1) |
