# Supplementary material for: Metabolic stimulation-elicited transcriptional responses and biosynthesis of acylated triterpenoids precursors in the medicinal plant Helicteres angustifolia
Source: BMC Plant Biol. 2022 Feb 25;22:86. doi: 10.1186/s12870-022-03429-8 (PMC8876399; doi:10.1186/s12870-022-03429-8)
Supplement: Supplementary file 19 — Additional file 19: Table S8. KEGG enrichment of DEGs from the NC vs EtOH comparison. [file 12870_2022_3429_MOESM19_ESM.doc]

Table S8 KEGG enrichment of DEGs from the NC vs EtOH comparison

| **Pathway id** | **Description** | **rich_factor** | **Pvalue_corrected** | **Num** |
| --- | --- | --- | --- | --- |
| **KO04075** | Plant hormone signal transduction | 6.830027694 | 1.79731E-28 | 59 |
| **KO00940** | Phenylpropanoid biosynthesis | 5.057611781 | 1.31184E-21 | 58 |
| **KO00195** | Photosynthesis | 7.174948682 | 2.14483E-21 | 42 |
| **KO04016** | KOK signaling pathway - plant | 3.851462019 | 1.05641E-19 | 70 |
| **KO04626** | Plant-pathogen interaction | 3.50958648 | 1.26347E-15 | 62 |
| **KO00010** | Glycolysis / Gluconeogenesis | 1.97337901 | 3.92035E-10 | 112 |
| **KO00053** | Ascorbate and aldarate metabolism | 3.256273585 | 4.55888E-09 | 39 |
| **KO00902** | Monoterpenoid biosynthesis | 18.78619376 | 1.15535E-08 | 9 |
| **KO00945** | Stilbenoid, diarylheptanoid and gingerol biosynthesis | 9.320282175 | 8.18867E-08 | 12 |
| **KO00941** | Flavonoid biosynthesis | 8.349419448 | 8.51347E-08 | 13 |
| **KO00196** | Photosynthesis - antenna proteins | 5.024429403 | 8.25868E-07 | 17 |
| **KO00906** | Carotenoid biosynthesis | 4.87348088 | 1.17503E-06 | 17 |
| **KO00903** | Limonene and pinene degradation | 3.450173326 | 1.26137E-06 | 25 |
| **KO00909** | Sesquiterpenoid and triterpenoid biosynthesis | 6.740815702 | 7.1576E-06 | 11 |
| **KO00905** | Brassinosteroid biosynthesis | 9.056997368 | 2.2617E-05 | 8 |
| **KO00071** | Fatty acid degradation | 1.849227028 | 7.05305E-05 | 58 |
| **KO00904** | Diterpenoid biosynthesis | 8.202938406 | 0.000163348 | 7 |
| **KO00561** | Glycerolipid metabolism | 1.863302824 | 0.000419808 | 46 |
| **KO00040** | Pentose and glucuronate interconversions | 2.132498479 | 0.00059963 | 31 |
| **KO00520** | Amino sugar and nucleotide sugar metabolism | 1.644755678 | 0.002112499 | 54 |
| **KO00380** | Tryptophan metabolism | 1.695130514 | 0.002497222 | 47 |
| **KO00630** | Glyoxylate and dicarboxylate metabolism | 1.580331757 | 0.002544907 | 60 |
| **KO00350** | Tyrosine metabolism | 1.760276465 | 0.004363101 | 37 |
| **KO00073** | Cutin, suberine and wax biosynthesis | 4.62938108 | 0.004446319 | 7 |
| **KO00908** | Zeatin biosynthesis | 8.096406738 | 0.007017438 | 4 |
| **KO00710** | Carbon fixation in photosynthetic organisms | 1.574743157 | 0.008036822 | 48 |
| **KO00340** | Histidine metabolism | 1.848728253 | 0.018947176 | 23 |
| **KO00130** | Ubiquinone and other terpenoid-quinone biosynthesis | 2.378469635 | 0.023309051 | 12 |
| **KO00030** | Pentose phosphate pathway | 1.514633914 | 0.030745809 | 40 |
| **KO00910** | Nitrogen metabolism | 1.766627793 | 0.039540747 | 21 |
| **KO00410** | beta-Alanine metabolism | 1.525907165 | 0.059231715 | 31 |
| **KO00620** | Pyruvate metabolism | 1.352630544 | 0.063394809 | 55 |
| **KO00480** | Glutathione metabolism | 1.411169484 | 0.066998771 | 42 |
| **KO00592** | alpha-Linolenic acid metabolism | 1.866536883 | 0.072968573 | 14 |
| **KO00460** | Cyanoamino acid metabolism | 1.770194936 | 0.088374167 | 15 |
| **KO00750** | Vitamin B6 metabolism | 2.199024052 | 0.104845905 | 8 |
| **KO00500** | Starch and sucrose metabolism | 1.313753619 | 0.105464292 | 51 |
| **KO00360** | Phenylalanine metabolism | 1.536366339 | 0.119124314 | 21 |
| **KO00944** | Flavone and flavonol biosynthesis | 5.80829179 | 0.142860374 | 2 |
| **KO00310** | Lysine degradation | 1.375756614 | 0.16981013 | 29 |
| **KO04146** | Peroxisome | 1.238589375 | 0.186250678 | 56 |
| **KO00591** | Linoleic acid metabolism | 1.917570017 | 0.275912447 | 6 |
| **KO00052** | Galactose metabolism | 1.294483635 | 0.333177104 | 25 |
| **KO00603** | Glycosphingolipid biosynthesis - globo and isoglobo series | 1.815091184 | 0.395356586 | 5 |
| **KO02010** | ABC transporters | 1.437864797 | 0.400591855 | 11 |
| **KO00943** | Isoflavonoid biosynthesis | 5.566279632 | 0.426281385 | 1 |
| **KO00330** | Arginine and proline metabolism | 1.216040284 | 0.43141641 | 28 |
| **KO00900** | Terpenoid backbone biosynthesis | 1.384359701 | 0.431449814 | 12 |
| **KO00960** | Tropane, piperidine and pyridine alkaloid biosynthesis | 1.468029793 | 0.451007389 | 8 |
| **KO04712** | Circadian rhythm - plant | 1.629155014 | 0.454073527 | 5 |
| **KO00400** | Phenylalanine, tyrosine and tryptophan biosynthesis | 1.309712855 | 0.456756684 | 14 |
| **KO00261** | Monobactam biosynthesis | 1.757772515 | 0.461505415 | 4 |
| **KO00051** | Fructose and mannose metabolism | 1.14040851 | 0.608364606 | 28 |
| **KO00100** | Steroid biosynthesis | 1.202535043 | 0.695988891 | 11 |
| **KO00740** | Riboflavin metabolism | 1.341272201 | 0.698942856 | 5 |
| **KO00966** | Glucosinolate biosynthesis | 2.385548414 | 0.744429821 | 1 |
| **KO00604** | Glycosphingolipid biosynthesis - ganglio series | 1.590365609 | 0.748514923 | 2 |
| **KO00860** | Porphyrin and chlorophyll metabolism | 1.149991775 | 0.749184142 | 12 |
| **KO00280** | Valine, leucine and isoleucine degradation | 1.043151649 | 0.814542803 | 31 |
| **KO00531** | Glycosaminoglycan degradation | 1.268266245 | 0.823600749 | 3 |
| **KO00730** | Thiamine metabolism | 1.113255926 | 0.824429629 | 8 |
| **KO00901** | Indole alkaloid biosynthesis | 1.855426544 | 0.831245932 | 1 |
| **KO00650** | Butanoate metabolism | 1.086692336 | 0.837760892 | 15 |
| **KO00260** | Glycine, serine and threonine metabolism | 1.052487314 | 0.843597503 | 28 |
| **KO00950** | Isoquinoline alkaloid biosynthesis | 1.07301776 | 0.873938759 | 8 |
| **KO00920** | Sulfur metabolism | 1.042197038 | 0.892268139 | 11 |
| **KO00562** | Inositol phosphate metabolism | 1.017983145 | 0.925292298 | 13 |
| **KO03013** | RNA transport | 0.228230144 | 1 | 12 |
| **KO00450** | Selenocompound metabolism | 0.984093637 | 1 | 8 |
| **KO00250** | Alanine, aspartate and glutamate metabolism | 0.974521905 | 1 | 24 |
| **KO00590** | Arachidonic acid metabolism | 0.942993255 | 1 | 6 |
| **KO01040** | Biosynthesis of unsaturated fatty acids | 0.931694874 | 1 | 13 |
| **KO00670** | One carbon pool by folate | 0.910845758 | 1 | 6 |
| **KO04070** | Phosphatidylinositol signaling system | 0.918149218 | 1 | 12 |
| **KO04933** | AGE-RAGE signaling pathway in diabetic complications | 0.885358174 | 1 | 9 |
| **KO00780** | Biotin metabolism | 0.834941945 | 1 | 3 |
| **KO00965** | Betalain biosynthesis | 0.795182805 | 1 | 2 |
| **KO03450** | Non-homologous end-joining | 0.759038132 | 1 | 2 |
| **KO03060** | Protein export | 0.830329006 | 1 | 9 |
| **KO00514** | Other types of O-glycan biosynthesis | 0.722111952 | 1 | 2 |
| **KO00511** | Other glycan degradation | 0.71822963 | 1 | 4 |
| **KO00062** | Fatty acid elongation | 0.679850947 | 1 | 4 |
| **KO03010** | Ribosome | 0.925601285 | 1 | 140 |
| **KO00640** | Propanoate metabolism | 0.784673781 | 1 | 16 |
| **KO00430** | Taurine and hypotaurine metabolism | 0.594617409 | 1 | 3 |
| **KO03430** | Mismatch repair | 0.587642425 | 1 | 3 |
| **KO00072** | Synthesis and degradation of ketone bodies | 0.534362845 | 1 | 2 |
| **KO00563** | Glycosylphosphatidylinositol (GPI)-anchor biosynthesis | 0.525947682 | 1 | 2 |
| **KO00270** | Cysteine and methionine metabolism | 0.783140466 | 1 | 24 |
| **KO03410** | Base excision repair | 0.541583964 | 1 | 3 |
| **KO03440** | Homologous recombination | 0.537228061 | 1 | 3 |
| **KO00220** | Arginine biosynthesis | 0.666472506 | 1 | 9 |
| **KO00565** | Ether lipid metabolism | 0.53650888 | 1 | 4 |
| **KO03022** | Basal transcription factors | 0.565104531 | 1 | 5 |
| **KO04130** | SNARE interactions in vesicular transport | 0.488746504 | 1 | 3 |
| **KO04141** | Protein processing in endoplasmic reticulum | 0.822009468 | 1 | 58 |
| **KO00300** | Lysine biosynthesis | 0.478248369 | 1 | 3 |
| **KO00660** | C5-Branched dibasic acid metabolism | 0.322682877 | 1 | 1 |
| **KO04122** | Sulfur relay system | 0.289157383 | 1 | 1 |
| **KO00600** | Sphingolipid metabolism | 0.477109683 | 1 | 5 |
| **KO03020** | RNA polymerase | 0.398381842 | 1 | 3 |
| **KO00230** | Purine metabolism | 0.690986437 | 1 | 27 |
| **KO00510** | N-Glycan biosynthesis | 0.474848499 | 1 | 6 |
| **KO00290** | Valine, leucine and isoleucine biosynthesis | 0.361707702 | 1 | 3 |
| **KO00770** | Pantothenate and CoA biosynthesis | 0.357832262 | 1 | 3 |
| **KO00760** | Nicotinate and nicotinamide metabolism | 0.350938821 | 1 | 3 |
| **KO03030** | DNA replication | 0.283031168 | 1 | 2 |
| **KO04145** | Phagosome | 0.627077399 | 1 | 27 |
| **KO03018** | RNA degradation | 0.514419208 | 1 | 13 |
| **KO00061** | Fatty acid biosynthesis | 0.331902388 | 1 | 4 |
| **KO00564** | Glycerophospholipid metabolism | 0.449113027 | 1 | 11 |
| **KO03420** | Nucleotide excision repair | 0.184772768 | 1 | 2 |
| **KO00020** | Citrate cycle (TCA cycle) | 0.476709087 | 1 | 17 |
| **KO00240** | Pyrimidine metabolism | 0.370627744 | 1 | 9 |
| **KO03008** | Ribosome biogenesis in eukaryotes | 0.314436778 | 1 | 7 |
| **KO00190** | Oxidative phosphorylation | 0.578314767 | 1 | 42 |
| **KO00970** | Aminoacyl-tRNA biosynthesis | 0.23404119 | 1 | 5 |
| **KO03040** | Spliceosome | 0.411301451 | 1 | 20 |
| **KO04144** | Endocytosis | 0.421059548 | 1 | 22 |
| **KO03015** | mRNA surveillance pathway | 0.19692027 | 1 | 5 |
| **KO04120** | Ubiquitin mediated proteolysis | 0.160797678 | 1 | 5 |
