# Supplementary material for: Metabolic stimulation-elicited transcriptional responses and biosynthesis of acylated triterpenoids precursors in the medicinal plant Helicteres angustifolia
Source: BMC Plant Biol. 2022 Feb 25;22:86. doi: 10.1186/s12870-022-03429-8 (PMC8876399; doi:10.1186/s12870-022-03429-8)
Supplement: Supplementary file 20 — Additional file 20: Table S9. KEGG enrichment of DEGs from the NC vs SA comparison. [file 12870_2022_3429_MOESM20_ESM.doc]

Table S9 KEGG enrichment of DEGs from the NC vs SA comparison

| **Pathway id** | **Kegg_pathway** | **rich_factor** | **P_value** | **DEGs** |
| --- | --- | --- | --- | --- |
| **map00010** | Glycolysis / Gluconeogenesis | 2.688860274 | 1.29448E-16 | 101 |
| **map04075** | Plant hormone signal transduction | 5.072509046 | 1.21651E-10 | 29 |
| **map00940** | Phenylpropanoid biosynthesis | 4.347962759 | 1.63715E-10 | 33 |
| **map00903** | Limonene and pinene degradation | 4.587519936 | 1.6637E-07 | 22 |
| **map00190** | Oxidative phosphorylation | 1.872457117 | 4.1505E-07 | 90 |
| **map04016** | MAPK signaling pathway - plant | 2.909711986 | 6.92989E-07 | 35 |
| **map00071** | Fatty acid degradation | 2.360547251 | 9.28696E-07 | 49 |
| **map00620** | Pyruvate metabolism | 2.155256053 | 1.1257E-06 | 58 |
| **map00195** | Photosynthesis | 4.129941221 | 3.28401E-05 | 16 |
| **map04146** | Peroxisome | 1.904884106 | 5.1976E-05 | 57 |
| **map00380** | Tryptophan metabolism | 2.179815088 | 5.74789E-05 | 40 |
| **map00561** | Glycerolipid metabolism | 2.264548956 | 6.02583E-05 | 37 |
| **map00909** | Sesquiterpenoid and triterpenoid biosynthesis | 7.40737164 | 0.00012426 | 8 |
| **map00053** | Ascorbate and aldarate metabolism | 2.775449561 | 0.000194686 | 22 |
| **map00340** | Histidine metabolism | 2.671912935 | 0.000318446 | 22 |
| **map04626** | Plant-pathogen interaction | 2.309310883 | 0.000519146 | 27 |
| **map00630** | Glyoxylate and dicarboxylate metabolism | 1.830666473 | 0.000614791 | 46 |
| **map00350** | Tyrosine metabolism | 2.156526466 | 0.000635581 | 30 |
| **map00480** | Glutathione metabolism | 1.929158283 | 0.000743302 | 38 |
| **map00410** | beta-Alanine metabolism | 2.156844303 | 0.000776827 | 29 |
| **map00906** | Carotenoid biosynthesis | 3.898407499 | 0.00320184 | 9 |
| **map00710** | Carbon fixation in photosynthetic organisms | 1.784536242 | 0.004087062 | 36 |
| **map00280** | Valine, leucine and isoleucine degradation | 1.728697689 | 0.008975657 | 34 |
| **map00310** | Lysine degradation | 1.863679974 | 0.010558186 | 26 |
| **map00904** | Diterpenoid biosynthesis | 7.082486919 | 0.011293031 | 4 |
| **map00902** | Monoterpenoid biosynthesis | 9.461759868 | 0.016887368 | 3 |
| **map00910** | Nitrogen metabolism | 2.033761987 | 0.02687806 | 16 |
| **map00030** | Pentose phosphate pathway | 1.659205056 | 0.027326216 | 29 |
| **map03010** | Ribosome | 1.238716657 | 0.034094464 | 124 |
| **map00945** | Stilbenoid, diarylheptanoid and gingerol biosynthesis | 4.694206446 | 0.04040817 | 4 |
| **map00051** | Fructose and mannose metabolism | 1.600037441 | 0.053182695 | 26 |
| **map00640** | Propanoate metabolism | 1.630220007 | 0.068161499 | 22 |
| **map00330** | Arginine and proline metabolism | 1.574909315 | 0.074066099 | 24 |
| **map00196** | Photosynthesis - antenna proteins | 2.679436423 | 0.086511199 | 6 |
| **map00908** | Zeatin biosynthesis | 6.116693248 | 0.136568873 | 2 |
| **map00650** | Butanoate metabolism | 1.64195399 | 0.14004477 | 15 |
| **map00020** | Citrate cycle (TCA cycle) | 1.355841325 | 0.162711792 | 32 |
| **map04712** | Circadian rhythm - plant | 2.461596063 | 0.166337492 | 5 |
| **map00965** | Betalain biosynthesis | 2.402986633 | 0.251720077 | 4 |
| **map00052** | Galactose metabolism | 1.408261934 | 0.270771479 | 18 |
| **map00040** | Pentose and glucuronate interconversions | 1.455155654 | 0.296668262 | 14 |
| **map00905** | Brassinosteroid biosynthesis | 3.421201308 | 0.312482428 | 2 |
| **map00430** | Taurine and hypotaurine metabolism | 1.796892082 | 0.316941875 | 6 |
| **map00500** | Starch and sucrose metabolism | 1.245512547 | 0.319105407 | 32 |
| **map00944** | Flavone and flavonol biosynthesis | 4.388062548 | 0.514044399 | 1 |
| **map00780** | Biotin metabolism | 1.682090643 | 0.530432235 | 4 |
| **map03450** | Non-homologous end-joining | 1.720319976 | 0.610704647 | 3 |
| **map00520** | Amino sugar and nucleotide sugar metabolism | 1.150540796 | 0.613996826 | 25 |
| **map00941** | Flavonoid biosynthesis | 1.940873819 | 0.622634937 | 2 |
| **map00073** | Cutin, suberine and wax biosynthesis | 1.998523537 | 0.622700532 | 2 |
| **map00250** | Alanine, aspartate and glutamate metabolism | 1.165704154 | 0.623293637 | 19 |
| **map00460** | Cyanoamino acid metabolism | 1.24819447 | 0.718381183 | 7 |
| **map00730** | Thiamine metabolism | 1.261567982 | 0.727076582 | 6 |
| **map00950** | Isoquinoline alkaloid biosynthesis | 1.21596914 | 0.779356496 | 6 |
| **map00740** | Riboflavin metabolism | 1.21596914 | 0.921350303 | 3 |
| **map00770** | Pantothenate and CoA biosynthesis | 1.081343985 | 0.968681766 | 6 |
| **map00270** | Cysteine and methionine metabolism | 0.986081471 | 0.997313158 | 20 |
| **map03040** | Spliceosome | 0.155365515 | 0.999999999 | 5 |
| **map00440** | Phosphonate and phosphinate metabolism | 1.127658532 | 1 | 2 |
| **map00061** | Fatty acid biosynthesis | 1.002985725 | 1 | 8 |
| **map00062** | Fatty acid elongation | 1.027230927 | 1 | 4 |
| **map00920** | Sulfur metabolism | 1.002096553 | 1 | 7 |
| **map00360** | Phenylalanine metabolism | 0.994883842 | 1 | 9 |
| **map00592** | alpha-Linolenic acid metabolism | 1.007239906 | 1 | 5 |
| **map01040** | Biosynthesis of unsaturated fatty acids | 0.974601875 | 1 | 9 |
| **map00591** | Linoleic acid metabolism | 0.965793671 | 1 | 2 |
| **map00450** | Selenocompound metabolism | 0.929331847 | 1 | 5 |
| **map02010** | ABC transporters | 0.790023003 | 1 | 4 |
| **map00261** | Monobactam biosynthesis | 0.663983149 | 1 | 1 |
| **map00531** | Glycosaminoglycan degradation | 0.638768599 | 1 | 1 |
| **map00290** | Valine, leucine and isoleucine biosynthesis | 0.728703528 | 1 | 4 |
| **map00670** | One carbon pool by folate | 0.68812799 | 1 | 3 |
| **map00600** | Sphingolipid metabolism | 0.72089599 | 1 | 5 |
| **map00900** | Terpenoid backbone biosynthesis | 0.697239645 | 1 | 4 |
| **map03022** | Basal transcription factors | 0.683082495 | 1 | 4 |
| **map00260** | Glycine, serine and threonine metabolism | 0.795135701 | 1 | 14 |
| **map00603** | Glycosphingolipid biosynthesis - globo and isoglobo series | 0.548507818 | 1 | 1 |
| **map00130** | Ubiquinone and other terpenoid-quinone biosynthesis | 0.598964027 | 1 | 2 |
| **map03430** | Mismatch repair | 0.591938056 | 1 | 2 |
| **map00660** | C5-Branched dibasic acid metabolism | 0.487562505 | 1 | 1 |
| **map03410** | Base excision repair | 0.545542911 | 1 | 2 |
| **map00750** | Vitamin B6 metabolism | 0.415331023 | 1 | 1 |
| **map00072** | Synthesis and degradation of ketone bodies | 0.403701754 | 1 | 1 |
| **map00760** | Nicotinate and nicotinamide metabolism | 0.530256245 | 1 | 3 |
| **map00590** | Arachidonic acid metabolism | 0.47494324 | 1 | 2 |
| **map00562** | Inositol phosphate metabolism | 0.591591082 | 1 | 5 |
| **map04145** | Phagosome | 0.736938182 | 1 | 21 |
| **map00565** | Ether lipid metabolism | 0.405323047 | 1 | 2 |
| **map03020** | RNA polymerase | 0.40129399 | 1 | 2 |
| **map04933** | AGE-RAGE signaling pathway in diabetic complications | 0.445915045 | 1 | 3 |
| **map00860** | Porphyrin and chlorophyll metabolism | 0.434399305 | 1 | 3 |
| **map00400** | Phenylalanine, tyrosine and tryptophan biosynthesis | 0.424056465 | 1 | 3 |
| **map00960** | Tropane, piperidine and pyridine alkaloid biosynthesis | 0.277267688 | 1 | 1 |
| **map04070** | Phosphatidylinositol signaling system | 0.462430417 | 1 | 4 |
| **map03420** | Nucleotide excision repair | 0.418777754 | 1 | 3 |
| **map00220** | Arginine biosynthesis | 0.447562921 | 1 | 4 |
| **map00564** | Glycerophospholipid metabolism | 0.555213293 | 1 | 9 |
| **map04130** | SNARE interactions in vesicular transport | 0.246159606 | 1 | 1 |
| **map00300** | Lysine biosynthesis | 0.240872168 | 1 | 1 |
| **map03030** | DNA replication | 0.213825082 | 1 | 1 |
| **map03008** | Ribosome biogenesis in eukaryotes | 0.475102939 | 1 | 7 |
| **map00230** | Purine metabolism | 0.541362506 | 1 | 14 |
| **map03018** | RNA degradation | 0.418529662 | 1 | 7 |
| **map00100** | Steroid biosynthesis | 0.165180751 | 1 | 1 |
| **map00970** | Aminoacyl-tRNA biosynthesis | 0.353628026 | 1 | 5 |
| **map00240** | Pyrimidine metabolism | 0.373337011 | 1 | 6 |
| **map00510** | N-Glycan biosynthesis | 0.119579904 | 1 | 1 |
| **map04120** | Ubiquitin mediated proteolysis | 0.29155158 | 1 | 6 |
| **map03050** | Proteasome | 0.225783979 | 1 | 4 |
| **map03015** | mRNA surveillance pathway | 0.178523771 | 1 | 3 |
| **map03013** | RNA transport | 0.373585052 | 1 | 13 |
| **map04141** | Protein processing in endoplasmic reticulum | 0.428285332 | 1 | 20 |
| **map04144** | Endocytosis | 0.289184638 | 1 | 10 |
