# Supplementary material for: Metabolic stimulation-elicited transcriptional responses and biosynthesis of acylated triterpenoids precursors in the medicinal plant Helicteres angustifolia
Source: BMC Plant Biol. 2022 Feb 25;22:86. doi: 10.1186/s12870-022-03429-8 (PMC8876399; doi:10.1186/s12870-022-03429-8)
Supplement: Supplementary file 21 — Additional file 21: Table S10. KEGG enrichment of DEGs from the NC vs MeJA comparison. [file 12870_2022_3429_MOESM21_ESM.doc]

Table S10 KEGG enrichment of DEGs from the NC vs MeJA comparison

| **Pathway id** | **Kegg_pathway** | **rich_factor** | **P_value** | **DEGs** |
| --- | --- | --- | --- | --- |
| **map00195** | Photosynthesis | 16.26164356 | 6.63469E-35 | 42 |
| **map04075** | Plant hormone signal transduction | 8.395877041 | 6.19375E-18 | 32 |
| **map00940** | Phenylpropanoid biosynthesis | 6.917213481 | 3.64483E-17 | 35 |
| **map04016** | MAPK signaling pathway - plant | 4.364567979 | 2.2747E-11 | 35 |
| **map00904** | Diterpenoid biosynthesis | 26.55932595 | 9.92901E-11 | 10 |
| **map00196** | Photosynthesis - antenna proteins | 10.04788659 | 8.03385E-10 | 15 |
| **map00591** | Linoleic acid metabolism | 10.14083354 | 2.68155E-09 | 14 |
| **map00010** | Glycolysis / Gluconeogenesis | 2.396014105 | 1.20161E-08 | 60 |
| **map00630** | Glyoxylate and dicarboxylate metabolism | 2.745999709 | 1.77592E-08 | 46 |
| **map00053** | Ascorbate and aldarate metabolism | 3.973939145 | 1.51179E-06 | 21 |
| **map00903** | Limonene and pinene degradation | 5.004567203 | 2.05097E-06 | 16 |
| **map00909** | Sesquiterpenoid and triterpenoid biosynthesis | 11.11105746 | 6.24651E-06 | 8 |
| **map00710** | Carbon fixation in photosynthetic organisms | 2.52809301 | 1.03574E-05 | 34 |
| **map00071** | Fatty acid degradation | 2.456896119 | 1.77704E-05 | 34 |
| **map00941** | Flavonoid biosynthesis | 10.18958755 | 4.40105E-05 | 7 |
| **map00906** | Carotenoid biosynthesis | 5.847611249 | 0.000186265 | 9 |
| **map00905** | Brassinosteroid biosynthesis | 12.82950491 | 0.000275412 | 5 |
| **map00561** | Glycerolipid metabolism | 2.386957007 | 0.000325534 | 26 |
| **map00380** | Tryptophan metabolism | 2.288805843 | 0.000333397 | 28 |
| **map00410** | beta-Alanine metabolism | 2.454340069 | 0.000717711 | 22 |
| **map00592** | alpha-Linolenic acid metabolism | 3.626063662 | 0.000756245 | 12 |
| **map00340** | Histidine metabolism | 2.914814111 | 0.000796559 | 16 |
| **map00945** | Stilbenoid, diarylheptanoid and gingerol biosynthesis | 8.801637087 | 0.001222912 | 5 |
| **map00130** | Ubiquinone and other terpenoid-quinone biosynthesis | 4.043007184 | 0.001974784 | 9 |
| **map00350** | Tyrosine metabolism | 2.264352789 | 0.002286377 | 21 |
| **map00966** | Glucosinolate biosynthesis | 16.22015977 | 0.003231928 | 3 |
| **map00260** | Glycine, serine and threonine metabolism | 2.044634659 | 0.003656576 | 24 |
| **map00360** | Phenylalanine metabolism | 2.487209604 | 0.004876051 | 15 |
| **map00620** | Pyruvate metabolism | 1.783660181 | 0.005180733 | 32 |
| **map00460** | Cyanoamino acid metabolism | 2.94217268 | 0.005348983 | 11 |
| **map04626** | Plant-pathogen interaction | 2.181015834 | 0.008665781 | 17 |
| **map00960** | Tropane, piperidine and pyridine alkaloid biosynthesis | 3.327212261 | 0.009797323 | 8 |
| **map00310** | Lysine degradation | 2.042879972 | 0.00997959 | 19 |
| **map00073** | Cutin, suberine and wax biosynthesis | 5.99557061 | 0.013827857 | 4 |
| **map00750** | Vitamin B6 metabolism | 3.737979207 | 0.016969968 | 6 |
| **map00910** | Nitrogen metabolism | 2.287982235 | 0.020774383 | 12 |
| **map00280** | Valine, leucine and isoleucine degradation | 1.754119714 | 0.021120934 | 23 |
| **map00330** | Arginine and proline metabolism | 1.771772979 | 0.041390732 | 18 |
| **map00900** | Terpenoid backbone biosynthesis | 2.353183801 | 0.041516466 | 9 |
| **map00950** | Isoquinoline alkaloid biosynthesis | 2.431938279 | 0.047652399 | 8 |
| **map00908** | Zeatin biosynthesis | 9.175039872 | 0.049512719 | 2 |
| **map00901** | Indole alkaloid biosynthesis | 8.410453216 | 0.056927521 | 2 |
| **map00030** | Pentose phosphate pathway | 1.630598073 | 0.065712912 | 19 |
| **map00040** | Pentose and glucuronate interconversions | 1.870914413 | 0.069234792 | 12 |
| **map00500** | Starch and sucrose metabolism | 1.459585016 | 0.093258403 | 25 |
| **map04712** | Circadian rhythm - plant | 2.953915276 | 0.105337427 | 4 |
| **map00520** | Amino sugar and nucleotide sugar metabolism | 1.380648955 | 0.205537769 | 20 |
| **map04146** | Peroxisome | 1.303341757 | 0.224556122 | 26 |
| **map00562** | Inositol phosphate metabolism | 1.597295922 | 0.239184285 | 9 |
| **map00400** | Phenylalanine, tyrosine and tryptophan biosynthesis | 1.484197626 | 0.397100913 | 7 |
| **map00051** | Fructose and mannose metabolism | 1.292337933 | 0.399401546 | 14 |
| **map00100** | Steroid biosynthesis | 1.486626755 | 0.426672069 | 6 |
| **map02010** | ABC transporters | 1.48129313 | 0.477674369 | 5 |
| **map03450** | Non-homologous end-joining | 1.720319976 | 0.606069412 | 2 |
| **map00670** | One carbon pool by folate | 1.376255981 | 0.609139824 | 4 |
| **map00640** | Propanoate metabolism | 1.111513641 | 0.743347134 | 10 |
| **map00860** | Porphyrin and chlorophyll metabolism | 1.085998263 | 0.850117717 | 5 |
| **map00052** | Galactose metabolism | 1.05619645 | 0.851181922 | 9 |
| **map03013** | RNA transport | 0.129317903 | 0.999999982 | 3 |
| **map00965** | Betalain biosynthesis | 0.901119987 | 1 | 1 |
| **map00511** | Other glycan degradation | 0.813914827 | 1 | 2 |
| **map00650** | Butanoate metabolism | 0.820976995 | 1 | 5 |
| **map01040** | Biosynthesis of unsaturated fatty acids | 0.812168229 | 1 | 5 |
| **map00250** | Alanine, aspartate and glutamate metabolism | 0.828263478 | 1 | 9 |
| **map00020** | Citrate cycle (TCA cycle) | 0.826215807 | 1 | 13 |
| **map00270** | Cysteine and methionine metabolism | 0.813517214 | 1 | 11 |
| **map00780** | Biotin metabolism | 0.630783991 | 1 | 1 |
| **map00072** | Synthesis and degradation of ketone bodies | 0.605552632 | 1 | 1 |
| **map00563** | Glycosylphosphatidylinositol (GPI)-anchor biosynthesis | 0.59601637 | 1 | 1 |
| **map00730** | Thiamine metabolism | 0.630783991 | 1 | 2 |
| **map04070** | Phosphatidylinositol signaling system | 0.693645626 | 1 | 4 |
| **map00920** | Sulfur metabolism | 0.644204927 | 1 | 3 |
| **map00480** | Glutathione metabolism | 0.761509849 | 1 | 10 |
| **map03020** | RNA polymerase | 0.601940986 | 1 | 2 |
| **map04141** | Protein processing in endoplasmic reticulum | 0.835156398 | 1 | 26 |
| **map00290** | Valine, leucine and isoleucine biosynthesis | 0.546527646 | 1 | 2 |
| **map00760** | Nicotinate and nicotinamide metabolism | 0.530256245 | 1 | 2 |
| **map00430** | Taurine and hypotaurine metabolism | 0.44922302 | 1 | 1 |
| **map03410** | Base excision repair | 0.409157183 | 1 | 1 |
| **map03440** | Homologous recombination | 0.405866375 | 1 | 1 |
| **map00062** | Fatty acid elongation | 0.385211598 | 1 | 1 |
| **map00220** | Arginine biosynthesis | 0.503508286 | 1 | 3 |
| **map00600** | Sphingolipid metabolism | 0.432537594 | 1 | 2 |
| **map03060** | Protein export | 0.418199331 | 1 | 2 |
| **map00190** | Oxidative phosphorylation | 0.717775228 | 1 | 23 |
| **map00565** | Ether lipid metabolism | 0.303992285 | 1 | 1 |
| **map00450** | Selenocompound metabolism | 0.278799554 | 1 | 1 |
| **map00770** | Pantothenate and CoA biosynthesis | 0.270335996 | 1 | 1 |
| **map00240** | Pyrimidine metabolism | 0.466671264 | 1 | 5 |
| **map04933** | AGE-RAGE signaling pathway in diabetic complications | 0.222957523 | 1 | 1 |
| **map00061** | Fatty acid biosynthesis | 0.188059823 | 1 | 1 |
| **map00230** | Purine metabolism | 0.464025005 | 1 | 8 |
| **map00970** | Aminoacyl-tRNA biosynthesis | 0.212176816 | 1 | 2 |
| **map00564** | Glycerophospholipid metabolism | 0.185071098 | 1 | 2 |
| **map03015** | mRNA surveillance pathway | 0.178523771 | 1 | 2 |
| **map04145** | Phagosome | 0.315830649 | 1 | 6 |
| **map03040** | Spliceosome | 0.326267582 | 1 | 7 |
| **map04120** | Ubiquitin mediated proteolysis | 0.14577579 | 1 | 2 |
| **map03018** | RNA degradation | 0.089684928 | 1 | 1 |
| **map04144** | Endocytosis | 0.260266174 | 1 | 6 |
| **map03010** | Ribosome | 0.524456649 | 1 | 35 |
