# Supplementary material for: Metabolic stimulation-elicited transcriptional responses and biosynthesis of acylated triterpenoids precursors in the medicinal plant Helicteres angustifolia
Source: BMC Plant Biol. 2022 Feb 25;22:86. doi: 10.1186/s12870-022-03429-8 (PMC8876399; doi:10.1186/s12870-022-03429-8)
Supplement: Supplementary file 22 — Additional file 22: Table S11. KEGG enrichment of DEGs from the NC vs MD comparison. [file 12870_2022_3429_MOESM22_ESM.doc]

Table S11 KEGG enrichment of DEGs from the NC vs MD comparison

| **Pathway id** | **Kegg_pathway** | **rich_factor** | **P_value** | **DEGs** |
| --- | --- | --- | --- | --- |
| **map04075** | Plant hormone signal transduction | 5.667042 | 8.45E-36 | 85 |
| **map00195** | Photosynthesis | 5.41127 | 2.89E-22 | 55 |
| **map00940** | Phenylpropanoid biosynthesis | 3.31458 | 2.39E-15 | 66 |
| **map00010** | Glycolysis / Gluconeogenesis | 1.856994 | 7.96E-14 | 183 |
| **map03010** | Ribosome | 1.397427 | 1.51E-09 | 367 |
| **map00196** | Photosynthesis - antenna proteins | 4.595879 | 1.63E-09 | 27 |
| **map04016** | MAPK signaling pathway - plant | 2.249847 | 8.32E-09 | 71 |
| **map00710** | Carbon fixation in photosynthetic organisms | 1.88945 | 3.09E-08 | 100 |
| **map00904** | Diterpenoid biosynthesis | 8.77368 | 3.21E-08 | 13 |
| **map00906** | Carotenoid biosynthesis | 4.127598 | 3.87E-08 | 25 |
| **map00190** | Oxidative phosphorylation | 1.522591 | 1.12E-07 | 192 |
| **map00350** | Tyrosine metabolism | 2.02758 | 1.7E-07 | 74 |
| **map00950** | Isoquinoline alkaloid biosynthesis | 2.858154 | 1.82E-07 | 37 |
| **map00945** | Stilbenoid, diarylheptanoid and gingerol biosynthesis | 6.26243 | 4.43E-07 | 14 |
| **map00620** | Pyruvate metabolism | 1.657179 | 1.11E-06 | 117 |
| **map00073** | Cutin, suberine and wax biosynthesis | 5.332366 | 3.05E-06 | 14 |
| **map00630** | Glyoxylate and dicarboxylate metabolism | 1.638279 | 4.98E-06 | 108 |
| **map00909** | Sesquiterpenoid and triterpenoid biosynthesis | 4.941 | 6.98E-06 | 14 |
| **map00480** | Glutathione metabolism | 1.702862 | 1.04E-05 | 88 |
| **map00941** | Flavonoid biosynthesis | 4.808651 | 2.06E-05 | 13 |
| **map00053** | Ascorbate and aldarate metabolism | 1.971547 | 0.000256 | 41 |
| **map00903** | Limonene and pinene degradation | 2.225492 | 0.000524 | 28 |
| **map00902** | Monoterpenoid biosynthesis | 7.212977 | 0.000853 | 6 |
| **map00908** | Zeatin biosynthesis | 6.994402 | 0.000977 | 6 |
| **map00040** | Pentose and glucuronate interconversions | 1.70358 | 0.003127 | 43 |
| **map00030** | Pentose phosphate pathway | 1.504748 | 0.003136 | 69 |
| **map00130** | Ubiquinone and other terpenoid-quinone biosynthesis | 2.283039 | 0.003154 | 20 |
| **map00960** | Tropane, piperidine and pyridine alkaloid biosynthesis | 2.113693 | 0.007134 | 20 |
| **map00561** | Glycerolipid metabolism | 1.446386 | 0.012884 | 62 |
| **map04626** | Plant-pathogen interaction | 1.532248 | 0.01314 | 47 |
| **map00910** | Nitrogen metabolism | 1.647296 | 0.015134 | 34 |
| **map00905** | Brassinosteroid biosynthesis | 3.912123 | 0.016811 | 6 |
| **map00260** | Glycine, serine and threonine metabolism | 1.407146 | 0.016865 | 65 |
| **map00966** | Glucosinolate biosynthesis | 5.495602 | 0.021012 | 4 |
| **map00410** | beta-Alanine metabolism | 1.445785 | 0.023593 | 51 |
| **map00051** | Fructose and mannose metabolism | 1.383953 | 0.031 | 59 |
| **map00901** | Indole alkaloid biosynthesis | 4.274357 | 0.047165 | 4 |
| **map00360** | Phenylalanine metabolism | 1.474723 | 0.053891 | 35 |
| **map00340** | Histidine metabolism | 1.481366 | 0.063469 | 32 |
| **map00944** | Flavone and flavonol biosynthesis | 5.017723 | 0.066402 | 3 |
| **map00400** | Phenylalanine, tyrosine and tryptophan biosynthesis | 1.508597 | 0.069109 | 28 |
| **map00965** | Betalain biosynthesis | 2.060851 | 0.097061 | 9 |
| **map00943** | Isoflavonoid biosynthesis | 6.411535 | 0.107399 | 2 |
| **map00071** | Fatty acid degradation | 1.248643 | 0.109333 | 68 |
| **map00520** | Amino sugar and nucleotide sugar metabolism | 1.245469 | 0.109414 | 71 |
| **map00591** | Linoleic acid metabolism | 1.840632 | 0.13126 | 10 |
| **map00500** | Starch and sucrose metabolism | 1.201699 | 0.145948 | 81 |
| **map00592** | alpha-Linolenic acid metabolism | 1.458912 | 0.17084 | 19 |
| **map00380** | Tryptophan metabolism | 1.225531 | 0.172965 | 59 |
| **map00330** | Arginine and proline metabolism | 1.250625 | 0.173768 | 50 |
| **map04146** | Peroxisome | 1.159172 | 0.20989 | 91 |
| **map00020** | Citrate cycle (TCA cycle) | 1.178947 | 0.211132 | 73 |
| **map00460** | Cyanoamino acid metabolism | 1.359336 | 0.252098 | 20 |
| **map04712** | Circadian rhythm - plant | 1.501238 | 0.390027 | 8 |
| **map00601** | Glycosphingolipid biosynthesis - lacto and neolacto series | 2.137178 | 0.550518 | 2 |
| **map00310** | Lysine degradation | 1.120197 | 0.56476 | 41 |
| **map00430** | Taurine and hypotaurine metabolism | 1.255672 | 0.58339 | 11 |
| **map00780** | Biotin metabolism | 1.282307 | 0.625541 | 8 |
| **map00750** | Vitamin B6 metabolism | 1.266476 | 0.639085 | 8 |
| **map00220** | Arginine biosynthesis | 1.108869 | 0.668557 | 26 |
| **map00740** | Riboflavin metabolism | 1.235959 | 0.676521 | 8 |
| **map00250** | Alanine, aspartate and glutamate metabolism | 1.052349 | 0.781775 | 45 |
| **map00052** | Galactose metabolism | 1.043738 | 0.844312 | 35 |
| **map00900** | Terpenoid backbone biosynthesis | 1.063052 | 0.860743 | 16 |
| **map02010** | ABC transporters | 1.053951 | 0.88885 | 14 |
| **map00670** | One carbon pool by folate | 1.04916 | 0.890647 | 12 |
| **map00920** | Sulfur metabolism | 1.036759 | 0.894869 | 19 |
| **map00100** | Steroid biosynthesis | 1.007377 | 0.954942 | 16 |
| **map00603** | Glycosphingolipid biosynthesis - globo and isoglobo series | 1.045359 | 0.968399 | 5 |
| **map00860** | Porphyrin and chlorophyll metabolism | 0.993466 | 0.97925 | 18 |
| **map03030** | DNA replication | 0.978031 | 1 | 12 |
| **map00640** | Propanoate metabolism | 0.960318 | 1 | 34 |
| **map00280** | Valine, leucine and isoleucine degradation | 0.949618 | 1 | 49 |
| **map00270** | Cysteine and methionine metabolism | 0.939649 | 1 | 50 |
| **map00562** | Inositol phosphate metabolism | 0.901974 | 1 | 20 |
| **map04130** | SNARE interactions in vesicular transport | 0.844446 | 1 | 9 |
| **map00531** | Glycosaminoglycan degradation | 0.730428 | 1 | 3 |
| **map00770** | Pantothenate and CoA biosynthesis | 0.82434 | 1 | 12 |
| **map00730** | Thiamine metabolism | 0.801442 | 1 | 10 |
| **map00062** | Fatty acid elongation | 0.783088 | 1 | 8 |
| **map00590** | Arachidonic acid metabolism | 0.724126 | 1 | 8 |
| **map00650** | Butanoate metabolism | 0.792749 | 1 | 19 |
| **map01040** | Biosynthesis of unsaturated fatty acids | 0.784244 | 1 | 19 |
| **map00604** | Glycosphingolipid biosynthesis - ganglio series | 0.457967 | 1 | 1 |
| **map00232** | Caffeine metabolism | 0.447316 | 1 | 1 |
| **map04070** | Phosphatidylinositol signaling system | 0.749114 | 1 | 17 |
| **map00300** | Lysine biosynthesis | 0.642684 | 1 | 7 |
| **map03410** | Base excision repair | 0.623825 | 1 | 6 |
| **map03440** | Homologous recombination | 0.618808 | 1 | 6 |
| **map00061** | Fatty acid biosynthesis | 0.716818 | 1 | 15 |
| **map03450** | Non-homologous end-joining | 0.43715 | 1 | 2 |
| **map00440** | Phosphonate and phosphinate metabolism | 0.429824 | 1 | 2 |
| **map00290** | Valine, leucine and isoleucine biosynthesis | 0.624951 | 1 | 9 |
| **map00072** | Synthesis and degradation of ketone bodies | 0.461631 | 1 | 3 |
| **map00563** | Glycosylphosphatidylinositol (GPI)-anchor biosynthesis | 0.454361 | 1 | 3 |
| **map00564** | Glycerophospholipid metabolism | 0.752454 | 1 | 32 |
| **map00511** | Other glycan degradation | 0.517059 | 1 | 5 |
| **map00660** | C5-Branched dibasic acid metabolism | 0.371683 | 1 | 2 |
| **map00565** | Ether lipid metabolism | 0.540732 | 1 | 7 |
| **map00790** | Folate biosynthesis | 0.443449 | 1 | 4 |
| **map03060** | Protein export | 0.584477 | 1 | 11 |
| **map00760** | Nicotinate and nicotinamide metabolism | 0.538973 | 1 | 8 |
| **map00261** | Monobactam biosynthesis | 0.253087 | 1 | 1 |
| **map04145** | Phagosome | 0.775805 | 1 | 58 |
| **map04933** | AGE-RAGE signaling pathway in diabetic complications | 0.566557 | 1 | 10 |
| **map00514** | Other types of O-glycan biosynthesis | 0.207942 | 1 | 1 |
| **map03430** | Mismatch repair | 0.338439 | 1 | 3 |
| **map00450** | Selenocompound metabolism | 0.425074 | 1 | 6 |
| **map03018** | RNA degradation | 0.638115 | 1 | 28 |
| **map03020** | RNA polymerase | 0.382398 | 1 | 5 |
| **map00240** | Pyrimidine metabolism | 0.616646 | 1 | 26 |
| **map04122** | Sulfur relay system | 0.166533 | 1 | 1 |
| **map03022** | Basal transcription factors | 0.325459 | 1 | 5 |
| **map00600** | Sphingolipid metabolism | 0.329736 | 1 | 6 |
| **map00230** | Purine metabolism | 0.619045 | 1 | 42 |
| **map00510** | N-Glycan biosynthesis | 0.273478 | 1 | 6 |
| **map03420** | Nucleotide excision repair | 0.212831 | 1 | 4 |
| **map03050** | Proteasome | 0.408789 | 1 | 19 |
| **map00970** | Aminoacyl-tRNA biosynthesis | 0.350455 | 1 | 13 |
| **map03008** | Ribosome biogenesis in eukaryotes | 0.310444 | 1 | 12 |
| **map03015** | mRNA surveillance pathway | 0.340235 | 1 | 15 |
| **map04120** | Ubiquitin mediated proteolysis | 0.296344 | 1 | 16 |
| **map03040** | Spliceosome | 0.213191 | 1 | 18 |
| **map04141** | Protein processing in endoplasmic reticulum | 0.399956 | 1 | 49 |
| **map03013** | RNA transport | 0.372424 | 1 | 34 |
| **map04144** | Endocytosis | 0.385794 | 1 | 35 |
