# Supplementary material for: Metabolic stimulation-elicited transcriptional responses and biosynthesis of acylated triterpenoids precursors in the medicinal plant Helicteres angustifolia
Source: BMC Plant Biol. 2022 Feb 25;22:86. doi: 10.1186/s12870-022-03429-8 (PMC8876399; doi:10.1186/s12870-022-03429-8)
Supplement: Supplementary file 23 — Additional file 23: Table S12. Relative expression of genes involved in triterpenoids biosynthesis. [file 12870_2022_3429_MOESM23_ESM.doc]

Table S12 Relative expression of genes involved in triterpenoids biosynthesis

| **Gene name** | **Gene ID** | **Relative expression of different treatment group** | | | | |
| --- | --- | --- | --- | --- | --- | --- |
|  |  | SA | MD | MeJA | NC | EtOH |
| **AACT1** | TRINITY_DN128339_c0_g1 | 11.820 | 12.420 | 14.930 | 13.310 | 13.050 |
| **AACT2** | TRINITY_DN130054_c0_g1 | 93.517 | 119.077 | 66.430 | 33.527 | 49.990 |
| **AACT3** | TRINITY_DN133837_c1_g2 | 17.150 | 14.263 | 16.420 | 19.473 | 10.183 |
| **AACT4** | TRINITY_DN115275_c0_g1 | 0.073 | 0.037 | 1.900 | 0.000 | 0.240 |
| **AACT5** | TRINITY_DN142178_c0_g1 | 0.000 | 0.597 | 0.293 | 0.167 | 0.177 |
| **CMK1** | TRINITY_DN132589_c2_g1 | 16.030 | 18.033 | 18.733 | 22.290 | 20.223 |
| **cmk2** | TRINITY_DN198205_c0_g1 | 0.000 | 0.897 | 0.000 | 0.000 | 0.000 |
| **DXR1** | TRINITY_DN100696_c0_g1 | 0.000 | 0.860 | 0.023 | 0.000 | 0.000 |
| **DXR2** | TRINITY_DN18805_c0_g1 | 0.000 | 0.573 | 0.000 | 0.000 | 0.000 |
| **DXR3** | TRINITY_DN127768_c1_g1 | 55.053 | 44.483 | 81.753 | 98.523 | 78.367 |
| **DXS1** | TRINITY_DN115944_c0_g1 | 1.900 | 1.350 | 1.947 | 2.113 | 2.330 |
| **DXS2** | TRINITY_DN129234_c0_g1 | 43.090 | 57.163 | 47.613 | 26.263 | 54.903 |
| **DXS3** | TRINITY_DN132357_c0_g1 | 147.583 | 56.710 | 77.323 | 13.513 | 24.600 |
| **dxs4** | TRINITY_DN132357_c0_g4 | 574.613 | 167.277 | 225.683 | 51.510 | 84.577 |
| **HDR1** | TRINITY_DN3223_c0_g1 | 0.643 | 0.673 | 0.000 | 0.000 | 0.000 |
| **HDR2** | TRINITY_DN332569_c0_g1 | 0.000 | 0.953 | 0.000 | 0.000 | 0.420 |
| **HDR3** | TRINITY_DN132069_c2_g2 | 959.570 | 637.163 | 1109.833 | 1402.097 | 1005.680 |
| **HDS1** | TRINITY_DN92669_c0_g1 | 0.037 | 4.830 | 0.017 | 0.000 | 0.000 |
| **HDS2** | TRINITY_DN128450_c0_g1 | 268.743 | 193.430 | 273.480 | 432.217 | 264.553 |
| **HMGR** | TRINITY_DN117231_c0_g1 | 62.767 | 84.460 | 84.237 | 50.940 | 54.643 |
| **HMGR** | TRINITY_DN144154_c0_g1 | 72.370 | 121.497 | 48.157 | 26.087 | 59.003 |
| **IDI1** | TRINITY_DN247361_c0_g1 | 0.000 | 0.000 | 0.157 | 0.000 | 0.503 |
| **IDI2** | TRINITY_DN123758_c0_g1 | 187.273 | 195.227 | 125.617 | 109.453 | 141.393 |
| **MCT1** | TRINITY_DN125819_c2_g1 | 10.950 | 11.190 | 13.607 | 19.853 | 14.207 |
| **MCT2** | TRINITY_DN125819_c2_g2 | 0.293 | 0.263 | 0.740 | 0.603 | 0.677 |
| **MK1** | TRINITY_DN326031_c0_g1 | 0.103 | 0.133 | 0.000 | 0.000 | 0.063 |
| **MK2** | TRINITY_DN126896_c0_g1 | 12.217 | 15.220 | 11.783 | 7.800 | 12.200 |
| **MVD1** | TRINITY_DN108491_c0_g1 | 0.043 | 2.413 | 0.047 | 0.000 | 0.207 |
| **MVD2** | TRINITY_DN129798_c2_g1 | 64.877 | 96.233 | 52.670 | 29.027 | 42.603 |
| **PMVK1** | TRINITY_DN141292_c0_g1 | 0.000 | 0.000 | 2.057 | 0.000 | 0.000 |
| **PMVK2** | TRINITY_DN191538_c0_g1 | 0.213 | 0.000 | 0.013 | 0.000 | 0.240 |
| **HMGS1** | TRINITY_DN119598_c0_g1 | 48.397 | 73.683 | 42.490 | 22.217 | 32.527 |
| **HMGS2** | TRINITY_DN128672_c5_g1 | 2.647 | 1.710 | 2.567 | 2.110 | 1.743 |
| **HMGS3** | TRINITY_DN83677_c0_g1 | 0.053 | 0.113 | 0.113 | 0.207 | 0.330 |
| **OSC1** | TRINITY_DN133651_c1_g1 | 4.247 | 18.503 | 17.383 | 17.763 | 13.630 |
| **OSC2** | TRINITY_DN131307_c0_g1 | 1.407 | 1.210 | 1.360 | 0.723 | 1.003 |
| **CYP450-1** | TRINITY_DN105670_c0_g1 | 9.140 | 10.383 | 2.710 | 2.220 | 9.940 |
| **CYP450-2** | TRINITY_DN127685_c0_g1 | 1.390 | 1.813 | 0.770 | 1.383 | 2.133 |
| **AT-1** | TRINITY_DN92932_c0_g1 | 6.897 | 9.820 | 6.070 | 3.497 | 10.837 |
| **AT-2** | TRINITY_DN131145_c0_g1 | 39.280 | 35.623 | 6.350 | 9.710 | 15.670 |
| **AT-3** | TRINITY_DN47973_c0_g1 | 7.877 | 27.130 | 5.950 | 1.860 | 6.450 |
