# Supplementary material for: Metabolic stimulation-elicited transcriptional responses and biosynthesis of acylated triterpenoids precursors in the medicinal plant Helicteres angustifolia
Source: BMC Plant Biol. 2022 Feb 25;22:86. doi: 10.1186/s12870-022-03429-8 (PMC8876399; doi:10.1186/s12870-022-03429-8)
Supplement: Supplementary file 24 — Additional file 24: Table S13. The relative expression levels of nine related genes in different treatment groups. [file 12870_2022_3429_MOESM24_ESM.doc]

Table S13 The relative expression levels of nine related genes in different treatment groups

| groups | number | HaAACT  relative expression | HaHMGR  relative expression | HaDXS  relative expression |
| --- | --- | --- | --- | --- |
| NC | 3 | 1.002±0.058 | 1.070±0.428 | 1.002±0.086 |
| EtOH | 3 | 1.506±0.081** | 2.817±0.170** | 2.518±0.059** |
| SA | 3 | 4.218±0.053** | 3.687±0.198** | 1.936±0.200** |
| MeJA | 3 | 3.528±0.046** | 2.665±0.285** | 2.156±0.115** |
| MD | 3 | 5.665±0.117** | 6.366±0.307** | 3.979±0.286** |
| P | - | 0.000 | 0.000 | 0.000 |
|  |  | HaOSC1  relative expression | HaOSC2  relative expression | HaCYPi1  relative expression |
| NC | 3 | 1.001±0.055 | 0.833±0.035 | 1.018±0.231 |
| EtOH | 3 | 0.762±0.193 | 1.638±0.065** | 4.809±0.177** |
| SA | 3 | 0.374±0.104** | 2.293±0.172** | 4.713±0.210** |
| MeJA | 3 | 0.977±0.126 | 2.104±0.067** | 1.248±0.265 |
| MD | 3 | 2.044±0.122** | 1.577±0.173** | 6.100±0.253** |
| P | - | 0.000 | 0.000 | 0.000 |
|  |  | HaTAT1  relative expression | HaTAT2  relative expression | HaTBT  relative expression |
| NC | 3 | 0.909±0.069 | 1.071±0.122 | 1.032±0.199 |
| EtOH | 3 | 3.894±0.073** | 2.558±0.284** | 5.874±0.126** |
| SA | 3 | 2.830±0.222** | 5.767±0.211** | 6.800±0.233** |
| MeJA | 3 | 2.481±0.247** | 0.857±0.182 | 5.442±0.050** |
| MD | 3 | 3.569±0.127** | 4.828±0.196** | 12.044±0.122** |
| P | - | 0.000 | 0.000 | 0.000 |

Bonferroni test：**P<0.01。
