# Supplementary material for: Metabolic stimulation-elicited transcriptional responses and biosynthesis of acylated triterpenoids precursors in the medicinal plant Helicteres angustifolia
Source: BMC Plant Biol. 2022 Feb 25;22:86. doi: 10.1186/s12870-022-03429-8 (PMC8876399; doi:10.1186/s12870-022-03429-8)
Supplement: Supplementary file 25 — Additional file 25: Table S14. Nucleotide sequence of ten candidate genes. [file 12870_2022_3429_MOESM25_ESM.doc]

Table S14 Protein sequence of ten candidate genes

| **Protein name** | **sequence** |
| --- | --- |
| **HaOSC1** | MWKLKIAEDGPLLSTVNNHIGRQHWEFDPNAGSPEERAELEKLRLRFKQNRFRQKQSSDLFMRMQLTKENSCGPILEAVKVEDKGEVTEEAITITLKRAINFYSSLQAHDGHWPAENAGPLFFLPPLVMALYITGALNAVLSPEHQKEIIRYIYNHQNEDGGWGLHIEGHSTMFGTALSYITLRLLGEGLEDGENMAVARGRKWILDHGGLVGIPSWGKFWVTVLGVYEWAGCNPMPPEFWLLPKFLPVHPGKMLCYCRLVYMPMSYLYGKRFVGPITDLIKQLRQELYNQRYQEINWNAARNTVAKEDLYYPHPLLQDLTWGFLHHVMEPILTRWPFSTLRDKALEVAIRHVHYEDENSRYLCIGCVEKVLCLIACWVEDPNSEAYKRHLARLPDYYWIAEDGLKMQTFGCQMWDAAFAIQAILSSNLSEEYAPTLRKANDFVKASQVQENPSGNFSAMYRHISKGAWTFSMQDHGWQVSDCTAEGLKCALLFSQMSNDLVGEKMETERLYDAVNVILSLQSDNGGFPAWEPQRAYSWLEKFNPTEFFEETLIEREYVECTASAIQALALFRKQHPMHRRREIDHCIARGAQFIEDTQNPDGSWYGCWGICYTYGTWFAVEGLAACGRNYNNSPALRKACEFLLSKQLPNGGWGESYLSSQNKIYTNLEGNSANLVQTAWALLSLIDAGQGGVDPTPIHSGIKVLINSQMEDGDFPQQEVTGVFMRNCTLNYSSFRNIFPIWALGEYRRRILLIA* |
| **HaOSC2** | MWKLKIAEGVDGPYLYSTNNYVGRQTWEFHPHAGTPQDRAEVEEARQSFYKNRLHVKLSSDLLWRMQFLKEKNFKQTIGAVRIEEGEEITYEKATAALRRAVHFFSALQASDGHWPAENSGPLFFLPPLVFSMYITGHLNTVFPEEHRREILRYIYHHQNADGGWGLHIEGHSIMFCTALSYICMRILGVEPDGGQDNACARARKWILDHGTVTHIPSWGKTWLSILGVFDWCGSNPMPPEFWILPSFLPMHPAKMWCYCRMVYMPMSYLYGKRFVGPITPLIEQLREELYLEPYNEINWKKVRHLCAPEDIYYRHPWIQDLMWDSLYICTEPLLTRWPLNKLVREKALQVTMKHIHYEDENSRYLTIGCVEKVLCMLACWAEEPNSVYFKRHLARIPDYLWVAEDGMKMQGLSTQEWDTGFAIQALLASNLIDEIGPVLKRGHDFIKKSQVKDNPSGDFKKMYRHISKGSWTLCDQDHGWQVSDCTAEGLKCCLLMSMLPPEIVGEKMEPQQLYDAVNVLLSLQSKNGGVPGWEPAGAQDWLEMLNPTEIFADLVVEHEYVECTASAMTALVLFKKLYPGHKQKEIENFITNAVRYLENEQMPDGSWYGKWGVCFIYGSWFALSGLAAAGKTYSNCLAMRKGVEFLLRTQRENGGWGESYKSCPDKIYVPLEEGRSNLVQTAWAMIGLIHAGQAERDLFPLHRAAKFIINSQLEDGDFPQQEITGAFLKNCMLNYAAYKNIYPLWALAEYRKLLSLSAKMV* |
| **HaOSC3** | MWKLEIAEGNGPWLFSTNNFVGRQVWKFDPDPSDSDAISSEQRAQFQTLQQNFSLHRHLVKASSDQLKNFQLIKGNHVDLSSLPALVRLKDNEEVTSEKVRVALRKAVGFISATQACDGHWPSENSGPLFCLPPLAMVLYLTGTTDAILSPEHKREILRYIYNHQNSDGGWGFHIEGHSTMMNTVLNYIALRFLGEGTEGGEDGAVEKARNWILDHGGATMIPSWGKAYLSVLGLYEWSGCNPMPPELWLLPSCFPFSPGRIWSFMRYFFAPLSYLYGKKFVGPITELILSLRDEIYTQPYHNIDWNKARHLCSKEDIYLPYPMVQILLWDSLYYIAEPIFNCWPFSKLREKALEIAIKLVHYEDENTRYLTQGSIQKVLHMMACWAENPSPTSESLKFHLARVPDFLWLAEDGMKMQLNGGSQLWDAILTTQAIISSNLVDEFGSTLRKAHEFIKKSQLQENPSGDFQSMHRHLSKGAWPFPTPDDGWQVSDCTAEALKTILLLSQMPPEIVGETIQAERLYDAVTVLLSLQSKNGGFTAWEPVRGPQWLQNINPTELFAAAAIELEYVECTSSAIQALVLFSQLYPGYRNKEIEISVAKAVQFVENSQMADGSWYGNWGICYTYGTSFALAGLAAVGKTCHNCQIVRKACQFLLSKQQESGGWGESYLSCPNLEYRHLEGNRSHLVQTSWAMMGLIHAGQADLDPQPLHKAARLLINSQMENGEFPQQEISGASLRTCMIHYAAYRNTFPLWALGEYYKHVLSPS* |
| **HaCYPi1** | MEHFYLPLLLLFVSFVTLSLFILFYKHKSIYSSPNLPPGKPGLPIIGESLEFLSTGWKGHPEKFIFDRITKYSSHLFKTNILGEPAVVFCGAAGNKFLFSNENKLVTAWWPSSVDKIFPSSLQTSSQEESKKMRKLLPQFLKPEALQRYIGIMDTIAKRHFASGWENKDQVLVFPLAKRYTFLLACRLFLSIEDPNHVAKFEAPFHLLAAGIISLPINLPGTPFNRGIKASNFIRKELLKVIKQRKVDLAEGKATPTQDILSHMLLTSDENGEFMKELDIADKILGLLIGGHDTASAACTFIVKYLAELPHIYEQVYNEQIQIANSKGPGEVLNWDDIQRMKYSWNVACEVMRLAPPLQGAFREAISDFIFNGFSIPKGWKLYWSANSTHRNGECFPEPEKFEPSRFEGNGPAPYTFVAFGGGPRMCPGKEYARLEILVFMHNLVTRFKWQKLLPDEKIIVDPMPMPAKGLPVLLYPHHSPNIYK* |
| **HaCYPi2** | MEHFYLPLLLLFVSFVTLSLFILFYKHKSIYSSPNLPPGKPGLPIIGESLEFLSTGWKGHPEKFIFDRITKYSSHLFKTNILGEPAVVFCGAAGNKFLFSNENKLVTAWWPSSVDKIFPSSLQTSSQEESKKMRKLLPQFLKPEALQRYIGIMDTIAKRHFASGWENKDQVLVFPLAKRYTFLLACRLFLSIEDPNHVAKFEAPFHLLAAGIISLPINLPGTPFNRGIKASNFIRKELLKVIKQRKVDLAEGKATPTQDILSHMLLTSDENGEFMKELDIADKILGLLIGGHDTASAACTFIVKYLAELPHIYEQVYNEQIQIANSKGPGEVLNWDDIQRMKYSWNVACEVMRLAPPLQGAFREAISDFIFNGFSIPKGWKVRGFAFLTLLFQLS* |
| **HaCYPi3** | MELSSLCGVTLIIFILFVSFSLFSLLFSTCNTNGDSKPNLPPGRTGLPFIGESLEFLSAGRKGHPEKFIVDRMAKYSSKIFRTSIFGEPVGFLCGAAANKFLFSNENKLVRVWLPKPMHKIFNSSRVQTSAPEEFLKLKKIVLNFLKPDALQRYVGMIDFIAQRHFESGWEGKQEVVVFPLVKNFTFSVACKLFLSIEDPEHIAKFADPFKVILAGVMSIAIDFPGTPFNRAIKASNKIKKELGKIIKQRKIDLANQKAFPRQDILSHMLLTADENGQYMNESEITDKILGVLVAAHDTITASITMIVKYLAELPDIYHKVLAEQTEIAKSKAHGKLLNLEDIRKMKYSWNVACEVLRLSPPSPGAFREAITEFTYSDFTVPKGWKLYWSASSTHRNPEYFPEPEKFDPSRFQGNGPAPYTFIPFGGGPRMCPGQDYARLLILIFMYNIVSRYKWEKLLPDEKTIVNPTPAPAKGLPVRLFPHLVA* |
| **HaCYPi4** | MELFFLCGLTLFILFVSFTLFSLLSSANGDSKPSLPPGRTGLYALICENLSFVWACRNGHPEKFIYERMAKYSSKIFRTSILLRRTSVVCGAAGNKFLFSNENKLVTGWYPRPVRKILNSSMVRQEEWKKMRKLPKFLKPDSLQRYVGMMDHIAQRHFESGWEGKQEVVVFPLAKNFTFWVACKLLLSIEDPEQVAKIAEPFKVMVAGVTSIPIDLPGTTANRAVKASIMIKKELGKILKQRKVDLAEKKASPMQDVISHMLVTTDENGQYMNEWEIAEMIVALLIGGHYAPSSTITMIVKYLAEFPEIYNKVLTEQMEIANSKASGELLNWKDILKMKYSWNVACEVLRLAPPAPGAFREALSEFNYSGFTIPKGSKLFWSANSTHRNPECFPEPEKFDPSRFEGNGPAPYTYVPFGGGPRMCPGVDYSRPLILVFMYNVVRRYKWEKLVPDEKTVVNPSPIPAKGLPVRLIPQTTA* |
| **HaTAT1** | MMKGVRCISSSMVQAVTHEAENQRVEFTPWDLHLLLLGPIQKGLLFPKPQIDQNNLIHHLKASLSHTLNYFQPLAGRLATTDHEDDTISFFIDCNNAGALFIHAEADGVTISDILKPVYIPSVVHSFFPLNGVNNYVAGASNPLLGVQVTELVDGIFVGVTMNHSVADGSSFWHFFNSWSEISRGNSFDLSKSPVFKRPFFDNINYPIRIPQSVVNNFHEELTFPPLKERVFHFSKQSIAKLKAKANAEAGTNNISSLQALLSHLWRSVTCNRKLDPNEEMTYIMLIGARQRLQDLLPEQYFGNAVKTGSITMKAKELQEHGIGDIAREMNKMIASHTKEELKKSLESWIASHKPLTLGSLMTNVMVISSSPRFNMYGNDFGWGRPIAVRSGAGNKFEGKVTLFCGAEEGSIDVEACLSPETLDAMANDHEFMDTVTV* |
| **HaTAT2** | MPSSSVTLVSKCTVVPDRKSEIKSLKLSVSDIPMLSCQYIQKGVLLKSPPFAFDDLVVFLKQSLSTTLSHFPPLAGRLTTEPDGHVYITCNDAGIDFLVVKSPCLSIHDILVPGDVPVCVKEFFTFDKTLSYSGHFKPLAAVQVTELVDGVFIGCTVNHAVTDGTSFWHYFNTFAEITKGASKISKSPDFSRNTVFNSQAVLKFPPGGPTVTFAGDEPLRERILHFSREAILKLKYRANYGCLLTKQTNSEVLGKLSNDSRKTVNGESNGKVKNSNDEISSFQSLCAQLWRSVTRARKLDPTKTTTFRMAVNCRHRLDPKLEPYYFGNAIQSIPTYAQASELLAKDLSWGADMLHKNVVAHDDATVRRGIADWEMKPRLFPLGNADGASITMGSSPRFPMYNNDFGWGLPLAVRSGRANKFDGKISAFPGREGNGSVDLEVVLAPDTMAGLLNDAEIMQYVSEMV* |
| **HaTBT** | MALLPTNTLVFTVRRQEPELVVPAKPTPHECKLLSDVDDQEGHRFQIQNINFYQCNPKMQGEDPAKVIKQALAQTLVFYYPFAGRLREMPNRKLVVDCTGEGVVFIEADADVTLDDFGDMLYPPFPCMEELLYDVPGTSDLLNCPLLLIQVTRLKCGGFIFAYRLNHTMSDAYGLCQLLSAIAEIARGAVTPSIPPIWERHLLTARNPPSITCLHNEFDPIPTNATVILRDNLVCRSFFFGPTQISALRKLVPNNLPFSTFDLISACIWRCRAKAVGYRPDEDVRLICTVNSRSRFNPPLPLGYYGNTLGFPAAKATAGELCKNPLTYAIELVKEAKGRVTEEYMKSAADLLVIRGRPNVNLVGSLIVSDLTRSTLREVDFGWGEAVFAGPATFLEIISFYTPQKNKEGDDGLIVPVCLPAPAMESFIKELDDMFKDEPAAIGRKKLFIRNSL* |
