# Supplementary material for: Metabolic stimulation-elicited transcriptional responses and biosynthesis of acylated triterpenoids precursors in the medicinal plant Helicteres angustifolia
Source: BMC Plant Biol. 2022 Feb 25;22:86. doi: 10.1186/s12870-022-03429-8 (PMC8876399; doi:10.1186/s12870-022-03429-8)
Supplement: Supplementary file 26 — Additional file 26: Table S15. Analysis of physicochemical properties of target gene coding proteins. [file 12870_2022_3429_MOESM26_ESM.doc]

Table S15 Analysis of physicochemical properties of target gene coding proteins

| Protein  name | Protein  length | molecular weight( KD) | Molecular formula | Isoelectric point | instability index | Hydrophilicity coefficient |
| --- | --- | --- | --- | --- | --- | --- |
| HaOSC1 | 756aa | 86.44 | C_3907_H_5939_N_1049_O_1105_S_36_ | 6.03 | 45.70 | -0.302 |
| HaOSC2 | 762aa | 87.79 | C_3990_H_6050_N_1046_O_110_1S_47_ | 6.25 | 50.25 | -0.277 |
| HaOSC3 | 767aa | 86.67 | C_3923_H_5960_N_1032_O_1125_S_34_ | 5.80 | 48.78 | -0.232 |
| HaCYPi1 | 485aa | 55.02 | C_2552_H_3917_N_645_O_678_S_17_ | 8.94 | 41.77 | -0.106 |
| HaCYPi2 | 395aa | 44.69 | C_2078_H_3211_N_521_O_553_S_11_ | 9.03 | 35.77 | -0.030 |
| HaCYPi3 | 486aa | 54.80 | C_2530_H_3934_N_644_O_680_S_17_ | 9.30 | 39.97 | -0.060 |
| HaCYPi4 | 478aa | 54.11 | C_2477_H_3866_N_638_O_670_S_25_ | 9.33 | 45.85 | -0.12 |
