# Supplementary material for: Metabolic stimulation-elicited transcriptional responses and biosynthesis of acylated triterpenoids precursors in the medicinal plant Helicteres angustifolia
Source: BMC Plant Biol. 2022 Feb 25;22:86. doi: 10.1186/s12870-022-03429-8 (PMC8876399; doi:10.1186/s12870-022-03429-8)
Supplement: Supplementary file 27 — Additional file 27: Table S16. Two-dimensional structure information of of target gene coding proteins. [file 12870_2022_3429_MOESM27_ESM.doc]

Table S16 Two-dimensional structure information of of target gene coding proteins.

| Protein  name | Alpha helix (%) | Random coil (%) | Extended  strand (%) | Beta  turn (%) |
| --- | --- | --- | --- | --- |
| HaOSC1 | 43.81 | 36.77 | 12.65 | 6.78 |
| HaOSC2 | 45.93 | 34.65 | 12.86 | 6.56 |
| HaOSC3 | 44.84 | 33.86 | 13.62 | 7.67 |
| HaCYPi1 | 47.42 | 34.43 | 15.05_7_ | 3.09 |
| HaCYPi2 | 54.43 | 27.09 | 14.18 | 4.30 |
| HaCYPi3 | 45.27 | 35.80 | 15.02 | 3.91 |
| HaCYPi4 | 47.49 | 34.52 | 14.02 | 3.97 |
| HaTAT1 | 34.70 | 41.10 | 19.41 | 4.79 |
| HaTAT2 | 36.99 | 41.94 | 16.99 | 4.09 |
| HaTBT | 33.11 | 42.38 | 20.31 | 4.19 |
