# Supplementary material for: Metabolic stimulation-elicited transcriptional responses and biosynthesis of acylated triterpenoids precursors in the medicinal plant Helicteres angustifolia
Source: BMC Plant Biol. 2022 Feb 25;22:86. doi: 10.1186/s12870-022-03429-8 (PMC8876399; doi:10.1186/s12870-022-03429-8)
Supplement: Supplementary file 28 — Additional file 28: Table S17. Summary table of OSC genes. [file 12870_2022_3429_MOESM28_ESM.doc]

Table S17 Summary table of OSC genes

| **Gene name** | **Accession number** | **Species name** |
| --- | --- | --- |
| **AaBAS** | PWA51067.1 | *Artemisia annua* |
| **AaLUS** | AJE29379.1 | *Artemisia annua* |
| **ACX** | BAF93208.1 | *Adiantum capillus-veneris* |
| **AsOXA1** | AAX14716.1 | *Aster sedifolius* |
| **BgbAS** | BAF80443.1 | *Bruguiera gymnorhiza* |
| **BgLUS** | BAF80444.1 | *Bruguiera gymnorhiza* |
| **BPW** | BAB83087.1 | *Betula platyphylla* |
| **BPX** | BAB83086.1 | *Betula platyphylla* |
| **BPX2** | BAB83085.1 | *Betula platyphylla* |
| **BPY** | BAB83088.1 | *Betula platyphylla* |
| **BS** | ABK76265.1 | *Gypsophila vaccaria* |
| **CAS1** | AAC04931.1 | *Arabidopsis thaliana* |
| **CPX** | BAD34644.1 | *Cucurbita pepo* |
| **CqbAS1** | ANY30852.1 | *Chenopodium quinoa* |
| **CrAS** | AFJ19235.1 | *Catharanthus roseus* |
| **CSOSC1** | BAB83253.1 | *Cheilocostus speciosus* |
| **EtAS** | BAE43642.1 | *Euphorbia tirucalli* |
| **GaBAS** | KHG28306.1 | *Gossypium arboreum* |
| **GgbAS1** | BAA89815.1 | *Glycyrrhiza glabra* |
| **GgCAS1** | BAA76902.1 | *Glycyrrhiza glabra* |
| **GgLUS1** | QBC36430.1 | *Glycyrrhiza glabra* |
| **IaAS1** | AIS39793.1 | *Ilex asprella var. asprella* |
| **IaAS2** | AIS39794.1 | *Ilex asprella var. asprella* |
| **IpAS1** | ARS25031.1 | *Ilex pubescens* |
| **KdCAS** | ADK35127.1 | *Kalanchoe daigremontiana* |
| **MlbAS** | AHF49822.1 | *Maesa lanceolata* |
| **MtAMY1** | XP_024634122.1 | *Medicago truncatula* |
| **NsbAS1** | ACH88049.1 | *Nigella sativa* |
| **NsbAS2** | ACH88048.1 | *Nigella sativa* |
| **OEA** | BAF63702.1 | *Olea europaea* |
| **OEW** | BAA86930.1 | *Olea europaea* |
| **LjAMY1** | BAE53429.1 | *Lotus japonicus* |
| **LjOSC3** | BAE53430.1 | *Lotus japonicus* |
| **LjOSC5** | BAE53431.1 | *Lotus japonicus* |
| **LjAMY2** | AAO33580.1 | *Lotus japonicus* |
| **PgOSCPNX** | BAA33460.1 | *Panax ginseng* |
| **PgOSCPNY** | BAA33461.1 | *Panax ginseng* |
| **PSM** | BAA97559.1 | *Pisum sativum* |
| **PsOSC** | BAA23533.1 | *Pisum sativum* |
| **PSY** | BAA97558.1 | *Pisum sativum* |
| **PtBS** | ABL07607.1 | *Polygala tenuifolia* |
| **RcCAS** | NP_001310632.1 | *Ricinus communis* |
| **SITTS1** | NP_001234604.1 | *Solanum lycopersicum* |
| **GvBS** | ABK76265.1 | *Gypsophila vaccaria* |
| **ToTRW** | BAA86932.1 | *Taraxacum officinale* |
| **WsOSC_BS** | AGA17940.1 | *Withania somnifera* |
| **WsOSC_CS** | ADG60271.1 | *Withania somnifera* |
| **WsOSC_LS** | AGA17939.1 | *Withania somnifera* |
| **GubAS** | ACV21067.1 | *Glycyrrhiza uralensis* |
| **AebAS** | ADK12003.1 | *Aralia elata* |
| **GsAS1** | ACO24697.1 | *Gentiana straminea* |
| **MdOSC1** | NP_001280946.1 | *Malus domestica* |
| **EsBAS** | APZ88354.1 | *Eleutherococcus senticosus* |
| **GaOSCBPY** | KHG28306.1 | *Gossypium arboreum* |
| **BcBAS2** | ADM89633.1 | *Bupleurum chinense* |
| **BcBAS1** | ABY90140.2 | *Bupleurum chinense* |
| **PqbAS2** | AGG09939.1 | *Panax quinquefolius* |
| **BvBAS** | AFF27506.1 | *Barbarea vulgaris* |
| **BvBAS1** | AFF27505.1 | *Barbarea vulgaris* |
| **EjAS** | AXG32171.1 | *Eriobotrya japonica* |
| **SsLUS** | TKY60766.1 | *Spatholobus suberectus* |
| **SsLUS1** | TKY44487.1 | *Spatholobus suberectus* |
| **TcOSC5** | QBO24615.1 | *Taraxacum coreanum* |
