# Supplementary material for: Metabolic stimulation-elicited transcriptional responses and biosynthesis of acylated triterpenoids precursors in the medicinal plant Helicteres angustifolia
Source: BMC Plant Biol. 2022 Feb 25;22:86. doi: 10.1186/s12870-022-03429-8 (PMC8876399; doi:10.1186/s12870-022-03429-8)
Supplement: Supplementary file 29 — Additional file 29: Table S18. Summary table of CYP450 genes. [file 12870_2022_3429_MOESM29_ESM.doc]

Table S18 Summary table of CYP450 genes

| **Gene name** | **Accession number** | **Species name** |
| --- | --- | --- |
| **CYP716A75** | KF318733 | *Maesa lanceolata* |
| **CYP51H10** | DQ680852 | *Avena strigosa* |
| **CYP714E19** | KT004520 | *Centella asiatica* |
| **CYP716A1** | EFH44718 | *Arabidopsis thaliana* |
| **CYP716A110** | KU878864 | *Aquilegia coerulea* |
| **CYP716A111** | KU878865 | *Aquilegia coerulea* |
| **CYP716A113V1** | AOG74849 | *Aquilegia coerulea* |
| **CYP716A12** | ABC59076 | *Medicago truncatula* |
| **CYP716A140** | AOG74836 | *Platycodon grandiflorus* |
| **CYP716A141** | AOG74838 | *Platycodon grandiflorus* |
| **CYP716A14v2** | KF309251 | *Artemisia annua* |
| **CYP716A15** | NP_001268115 | *Vitis vinifera* |
| **CYP716A154** | AEX07772 | *Catharanthus roseus* |
| **CYP716A17** | NP_001268076 | *Vitis vinifera* |
| **CYP716A179** | BAW34647 | *Glycyrrhiza uralensis* |
| **CYP716A180** | AHL46848 | *Solanum lycopersicum* |
| **CYP716A2** | NP_198463 | *Arabidopsis thaliana* |
| **CYP716A244** | KX354739 | *Vitis vinifera* |
| **CYP716A249** | KY385302.1 | *Polygala tenuifolia* |
| **CYP716A252** | JQ958967 | *Ocimum basilicim* |
| **CYP716A266** | MG708188 | *Lagerstroemia speciosa* |
| **CYP716A44** | AK329870 | *Solanum lycopersicum* |
| **CYP716A46** | XM_004243858 | *Solanum lycopersicum* |
| **CYP716A47** | AEY75213 | *Panax ginseng* |
| **CYP716A48** | BAP59949 | *Olea europaea* |
| **CYP716A51** | BAP59952 | *Lotus japonicus* |
| **CYP716A52V2** | AFO63032 | *Panax ginseng* |
| **CYP716A78** | KX343075 | *Chenopodium quinoa* |
| **CYP716A79** | KX343076 | *Chenopodium quinoa* |
| **CYP716A80** | ALR73782 | *Barbarea vulgaris* |
| **CYP716A81** | ALR73781 | *Barbarea vulgaris* |
| **CYP716A83** | AOG74832 | *Centella asiatica* |
| **CYP716A86** | AOG74831 | *Centella asiatica* |
| **CYP716A94** | KT150521 | *Kalopanax septemlobus* |
| **CYP716AL1** | JN565975 | *Catharanthus roseus* |
| **CYP716C11** | KU878852 | *Centella asiatica* |
| **CYP716C55** | MG708191 | *Lagerstroemia speciosa* |
| **CYP716E26** | XM_004241773 | *Solanum lycopersicum* |
| **CYP716E41** | KU878851 | *Centella asiatica* |
| **CYP716S5** | KU878856 | *Platycodon grandiflorus* |
| **CYP716Y1** | KC963423 | *Bupleurum falcatum* |
| **CYP71D353** | AHB62239 | *Lotus japonicus* |
| **CYP72A154** | H1A988 | *Glycyrrhiza uralensis* |
| **CYP72A397** | KT150517 | *Kalopanax septemlobus* |
| **CYP72A552** | MH252571 | *Barbarea vulgaris* |
| **CYP72A61** | ABC59088 | *Medicago truncatula* |
| **CYP72A61v2** | AB558145 | *Medicago truncatula* |
| **CYP72A63** | AB558146 | *Medicago truncatula* |
| **CYP72A67** | DQ335780 | *Medicago truncatula* |
| **CYP72A68** | ABC59077 | *Medicago truncatula* |
| **CYP72A68v2** | AB558150 | *Medicago truncatula* |
| **CYP72Q58** | AIT72031 | *Cucumis sativus* |
| **CYP749A63** | MF596155 | *Crataegus pinnatifida* |
| **CYP87D16** | KF318735 | *Maesa lanceolata* |
| **CYP88D6** | BAG68929 | *Medicago truncatula* |
| **CYP88L2** | NP_001295886 | *Cucumis sativus* |
| **CYP93E1** | AF135485 | *Glycine max* |
| **CYP93E2** | DQ335790 | *Medicago truncatula* |
| **CYP93E3** | AB437320 | *Glycyrrhiza uralensis* |
| **CYP93E4** | KF906535 | *Arachis hypogaea* |
| **CYP93E5** | KF906536 | *Cicer arietinum* |
| **CYP93E6** | KF906537 | *Glycyrrhiza glabra* |
| **CYP93E7** | KF906538 | *Lens culinaris* |
| **CYP93E8** | KF906539 | *Pisum sativum* |
| **CYP93E9** | KF906540 | *Phaseolus vulgaris* |
