# Supplementary material for: Metabolic stimulation-elicited transcriptional responses and biosynthesis of acylated triterpenoids precursors in the medicinal plant Helicteres angustifolia
Source: BMC Plant Biol. 2022 Feb 25;22:86. doi: 10.1186/s12870-022-03429-8 (PMC8876399; doi:10.1186/s12870-022-03429-8)
Supplement: Supplementary file 30 — Additional file 30: Table S19. Summary table of BAHD genes. [file 12870_2022_3429_MOESM30_ESM.doc]

Table S19 Summary table of BAHD genes

| **Gene name** | **Accession number** | **Species name** |
| --- | --- | --- |
| **AcAT16** | HO772640 | *Actinidia chinensis* |
| **AdAT9** | HO772637 | *Actinidia deliciosa* |
| **AMAT** | AAW22989 | *Vitis labrusca* |
| **AsHHT1** | BAC78633 | *Avena sativa* |
| **At5MAT** | Q9LJB4 | *Arabidopsis thaliana* |
| **AtACT** | Q9FNP9 | *Arabidopsis thaliana* |
| **AtHCT** | NP_199704 | *Arabidopsis thaliana* |
| **AtHHT1** | ACY78659 | *Arabidopsis thaliana* |
| **AtSCT** | Q8VZU3 | *Arabidopsis thaliana* |
| **AtSHT** | AEC06845 | *Arabidopsis thaliana* |
| **BanAAT** | CAC09063 | *Banana* |
| **TcBAPT** | AAL92459 | *Taxus cuspidata* |
| **CbBEAT** | AAF04787 | *Clarkia breweri* |
| **CbBEBT** | AAN09796 | *Clarkia breweri* |
| **CbRAS** | CAK55166 | *Plectranthus scutellarioides* |
| **CcHCT** | DQ104740 | *Cynara cardunculus* |
| **CcHCT1** | EF137954 | *Coffea canephora* |
| **AtCer2** | AAB17946 | *Arabidopsis thaliana* |
| **AtCHAT** | AAN09797 | *Arabidopsis thaliana* |
| **CmAAT1** | CAA94432 | *Cucumis melo* |
| **CmAAT2** | AAL77060 | *Cucumis melo* |
| **CmAAT3** | AAW51125 | *Cucumis melo* |
| **CmAAT4** | AAW51126 | *Cucumis melo* |
| **CsHCT** | AEJ88365 | *Cucumis sativus* |
| **CsHQT** | DQ915589 | *Cynara scolymus* |
| **CrDAT** | AAC99311 | *Catharanthus roseus* |
| **TcDBAT** | AAF27621 | *Taxus cuspidata* |
| **TcDBBT** | Q9FPW3 | *Taxus cuspidata* |
| **TcDBNTBT** | AAM75818 | *Taxus cuspidata* |
| **Cm3MAT1** | AAQ63615 | *Chrysanthemum morifolium* |
| **Cm3MAT2** | AAQ63616 | *Chrysanthemum morifolium* |
| **Cm3MAT3** | BAF50706 | *Chrysanthemum morifolium* |
| **Dp3MAT** | Q8GSN8 | *Dahlia pinnata* |
| **EcHQT** | AFF19202 | *Erythroxylum coca* |
| **Zmglossy2** | DAA36076 | *Zea mays* |
| **DcBT** | CAB06430 | *Dianthus caryophyllus* |
| **HcHCT** | JQ779021 | *Hibiscus cannabinus* |
| **LaHMT** | BAD89275 | *Lupinus albus* |
| **HvACT** | AAO73071 | *Hordeum vulgare* |
| **LaAT** | AB581532 | *Lupinus angustifolius* |
| **LaAT1** | DQ886904 | *Lavandula angustifolia* |
| **LaAT2** | DQ886905 | *Lavandula angustifolia* |
| **Lp3MAT1** | AAS77404 | *Lamium purpureum* |
| **MdAAT1** | AAU14879 | *Malus domestica* |
| **NtBEBT** | AAN09798 | *Nicotiana tabacum* |
| **NtHQT** | CAE46932 | *Nicotiana tabacum* |
| **NtMAT1** | 2XR7 | *Nicotiana tabacum* |
| **Pf3AT** | Q9MBC1 | *Perilla frutescens* |
| **Pf5MaT** | Q9LJB4 | *Perilla frutescens* |
| **Cfpun1** | ADN97116 | *Capsicum frutescens* |
| **Gt5AT** | Q9ZWR8 | *Gentiana triflora* |
| **RhAAT1** | AAW31948 | *Rosa hybrid* |
| **FaSAAT** | AAG13130 | *Fragaria ananassa* |
| **SalAT** | Q94FT4 | *Papaver somniferum* |
| **Pc3MAT** | AAO38058 | *Pericallis cruenta* |
| **Ss5MaT1** | Q8W1W9 | *Salvia splendens* |
| **Ss5MaT2** | AAR26385 | *Salvia splendens* |
| **FvVAAT** | AX025504 | *Fragaria vesca* |
| **Gh3MAT1** | AAS77402 | *Glandularia hybrida* |
| **vinorine synthase** | CAD89104 | *Rauvolfia serpentina* |
